# Three-component reactions of aromatic amines, 1,3-dicarbonyl compounds, and α-bromoacetaldehyde acetal to access *N*-(hetero)aryl-4,5-unsubstituted pyrroles

**DOI:** 10.3762/bjoc.16.241

**Published:** 2020-11-30

**Authors:** Wenbo Huang, Kaimei Wang, Ping Liu, Minghao Li, Shaoyong Ke, Yanlong Gu

**Affiliations:** 1 National Biopesticide Engineering Research Centre, Hubei Biopesticide Engineering Research Centre, Hubei Academy of Agricultural Sciences, 8 Nanhu Avenue, Hongshan District, Wuhan 430064, China; 2 School of Chemistry and Chemical Engineering, The Key Laboratory for Green Processing of Chemical Engineering of Xinjiang Bingtuan, Shihezi University, Shihezi City 832004, China; 3 Key laboratory of Material Chemistry for Energy Conversion and Storage, Ministry of Education, Hubei Key Laboratory of Material Chemistry and Service Failure, School of Chemistry and Chemical Engineering, Huazhong University of Science and Technology, 1037 Luoyu road, Hongshan District, Wuhan 430074, China

**Keywords:** acid catalyst, [1 + 2 + 2] annulation, KI, pyrazolo[3,4-*b*]pyridine, pyrroles

## Abstract

*N*-(Hetero)aryl-4,5-unsubstituted pyrroles were synthesized from (hetero)arylamines, 1,3-dicarbonyl compounds, and α-bromoacetaldehyde acetal by using aluminum(III) chloride as a Lewis acid catalyst through [1 + 2 + 2] annulation. This new versatile methodology provides a wide scope for the synthesis of different functional *N*-(hetero)aryl-4,5-unsubstituted pyrrole scaffolds, which can be further derived to access multisubstituted pyrrole-3-carboxamides. In the presence of 1.2 equiv of KI, a polysubstituted pyrazolo[3,4-*b*]pyridine derivative was also successfully synthesized.

## Introduction

Among nitrogen-containing heterocycles, pyrroles have garnered significant attention in the literature because of their presence in various natural products [1–4] and pharmaceutically relevant drugs [5–6]. Accordingly, numerous synthetic methods to construct pyrrole skeletons were reported, including the classical Hantzsch [7–8] and the Paal–Knorr pyrrole syntheses [9–11], which have been developed to harvest the pyrrole frameworks. In the past few years, the interest in developing new methods to synthesize this heterocyclic motif has rapidly grown; transition metal-catalyzed cyclization [12–14] and multicomponent reactions [15–18] are some of the commonly used approaches for the construction of pyrrole scaffolds. Additionally, the biocatalytic synthesis of substituted pyrroles was also developed [19]. Though sustained efforts have been achieved to develop efficient synthetic methods for the preparation of this structural motif [20–23], the development of cost-effective methods to access functionalized pyrrole skeletons has remained an ongoing challenge.

**Figure 1:**
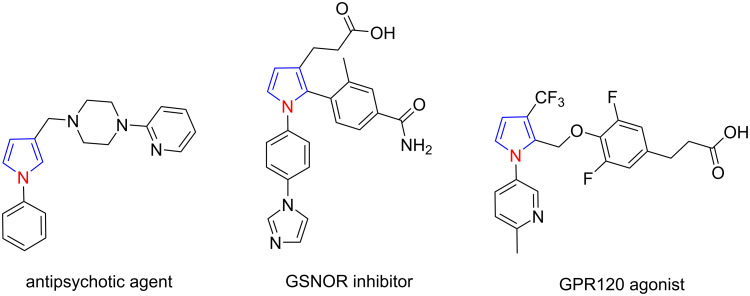
Representative biologically active *N*-(hetero)aryl-4,5-unsubstituted pyrrole scaffolds.

**Scheme 1:**
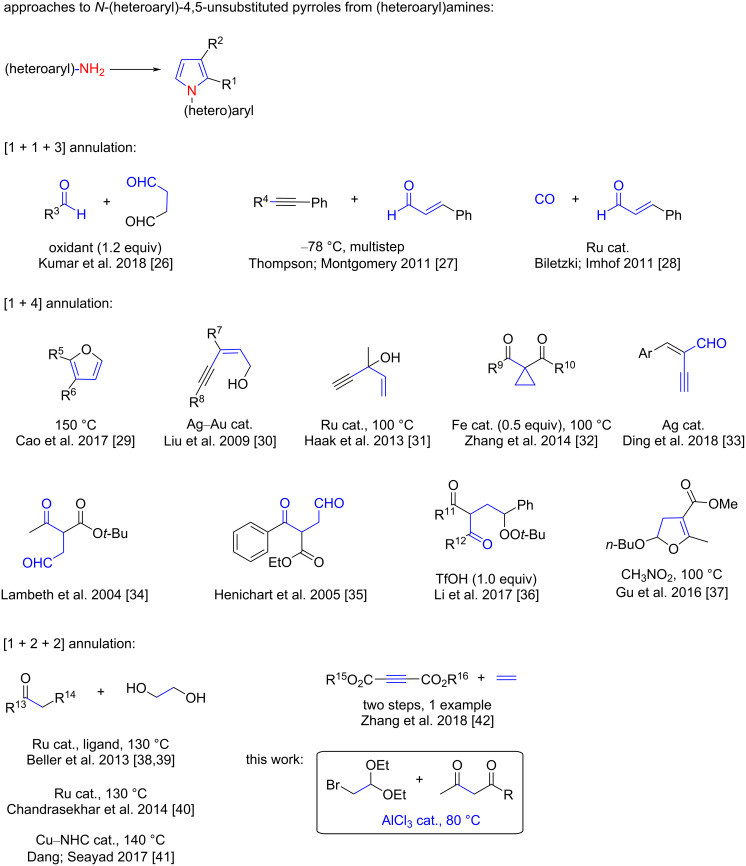
Typical routes to *N*-(heteroaryl)-4,5-unsubstituted pyrroles.

*N*-(Hetero)aryl-4,5-unsubstituted pyrroles are one of the most important types of pyrroles, which are frequently used as a core scaffold in pharmaceuticals (Figure 1) [24–25]. Therefore, many efforts have been paid to the synthesis of these privileged pyrroles. (Hetero)arylamines are readily available chemicals. The direct conversion of (hetero)arylamines into *N*-(hetero)aryl-4,5-unsubstituted pyrroles has a high intrinsic synthetic potential. At present, the transformations can generally be realized through the following three approaches (Scheme 1): (i) [1 + 1 + 3] annulation, in which (hetero)arylamines are reacted with a C_3_ donor and a C_1_ donor to construct pyrrole scaffolds. Kumar et al. [26] developed a proline-catalyzed Mannich reaction–cyclization sequence of succinaldehyde and an in situ*-*generated arylimine, in which the succinaldehyde contributes three carbon atoms to the pyrrole ring. α,β-Unsaturated aldehydes have also been used as the C_3_ donor to construct pyrrole scaffolds [27–28]; (ii) [1 + 4] annulation, in which (hetero)arylamines are reacted with a C_4_ donor to form the pyrrole ring; many functional molecules, such as bioderived furans [29], (*Z*)-enynols [30], 1-vinylpropargyl alcohols [31], doubly activated cyclopropanes [32], and enynals [33], can be used as C_4_ counter reagents. The carbon-based 1,4-biselectrophiles, such as the 1,4-dicarbonyl compounds [34–35], γ-carbonyl *tert*-butyl peroxides [36], and dihydrofurans [37] have also been reported to construct the pyrrole skeletons through this type of annulation; and (iii) [1 + 2 + 2] annulation, in which (hetero)arylamines are reacted with two different molecules, and each of them contributes two carbon atoms to construct a pyrrole ring [38–42]. Among these three approaches, the third is considered the most attractive route for *N*-(hetero)aryl-4,5-unsubstituted pyrrole synthesis. The reason is twofold: (i) the strategy uses easily available substrates and (ii) permits to synthesize pyrroles with a high potential of molecular diversity and complexity. However, to date, the productivity for creating molecular diversity and complexity has yet to be fully displayed. In addition, some of the reported approaches were established on the basis of using expensive and nonrecyclable homogeneous metal catalysts. To alleviate all these problems, herein, we used easily available α-bromoacetaldehyde acetal (**2a**) and a simple 1,3-dicarbonyl compound as a reagent couple to react with (hetero)arylamines. The established [1 + 2 + 2] annulation reaction provided a straightforward approach for accessing various *N*-(hetero)aryl-4,5-unsubstituted pyrroles, and some of the pyrrole products are not accessible with the methods reported hitherto.

## Results and Discussion

Initially, a mixture of aniline (**1a**), α-bromoacetaldehyde acetal (**2a**), and ethyl acetoacetate (**3a**) was treated under the conditions; the obtained results are listed in Table 1. The mixture was heated in 1,4-dioxane at 80 °C. No reaction occurred in the absence of the catalyst (Table 1, entry 1); however, in the presence of the strong Lewis acid Bi(OTf)_3_, the expected product **4a** was obtained in 36% yield after 6 h of reaction (Table 1, entry 2). To our surprise, the *N*-aryl-4,5-unsubstituted pyrrole derivative **4a** was isolated in 80% yield when 10 mol % of AlCl_3_ was used as the catalyst (Table 1, entry 3). FeCl_3_ and NiCl_2_ were also proven to catalyze this reaction, but the yield of **4a** was inferior to those obtained with AlCl_3_ (Table 1, entries 4 and 5). *p*-Toluenesulfonic acid (PTSA), a strong Brønsted acid, also exhibited a promising catalytic ability, and the yield of **4a** reached 73% (Table 1, entry 6). When HOAc was used, only unreacted starting materials were recovered (Table, entry 7). The effect of the solvent on the model reaction was also examined. Anhydrous ethanol, acetonitrile, toluene, and DMSO did not bring any improvement with respect to the yield of **4a** (Table 1, entries 8–11). The decrease of the catalyst loading from 10 to 5 mol % resulted in a slight decrease of the reaction yield (Table 1, entry 12). Further investigations revealed that the reaction was also affected by the temperature and time; a yield of only 51% was obtained at 50 °C (Table 1, entries 13 and 14). Therefore, the optimized reaction conditions were confirmed as the following: 10 mol % of AlCl_3_ as a catalyst, 1,4-dioxane as a solvent, 6 h, and 80 °C. It is worth noting that the reaction can be effectively scaled up with similar efficiency. In a gram-scale synthesis of **4a**, the corresponding pyrrole product was obtained in 72% yield (Table 1, entry 15).

**Table 1:** Optimization of the conditions for the reaction between **1a**, **2a**, and **3a**.^a^

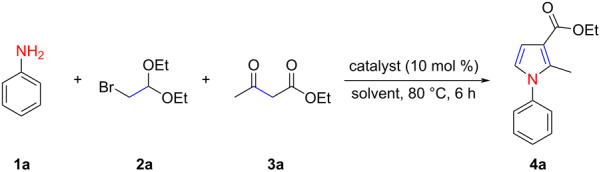			

entry	catalyst	solvent	yield (%)^b^

1	—	1,4-dioxane	0
2	Bi(OTf)_3_	1,4-dioxane	36
3	AlCl_3_	1,4-dioxane	80
4	FeCl_3_	1,4-dioxane	44
5	NiCl_2_	1,4-dioxane	21
6	PTSA	1,4-dioxane	73
7	HOAc	1,4-dioxane	trace
8	AlCl_3_	EtOH	31
9	AlCl_3_	MeCN	50
10	AlCl_3_	PhMe	27
11	AlCl_3_	DMSO	62
12^c^	AlCl_3_	1,4-dioxane	55
13^d^	AlCl_3_	1,4-dioxane	51
14^e^	AlCl_3_	1,4-dioxane	40
15^f^	AlCl_3_	1,4-dioxane	72

^a^**1a**: 0.5 mmol, **2a**: 0.6 mmol, **3a**: 0.6 mmol, catalyst: 0.05 mmol, solvent: 1 mL, 80 °C, 6 h. ^b^Isolated yield, calculated with respect to **1a**. ^c^AlCl_3_: 0.025 mmol. ^d^50 °C. ^e^2 h. ^f^10 mmol-scale reaction.

**Scheme 2:**
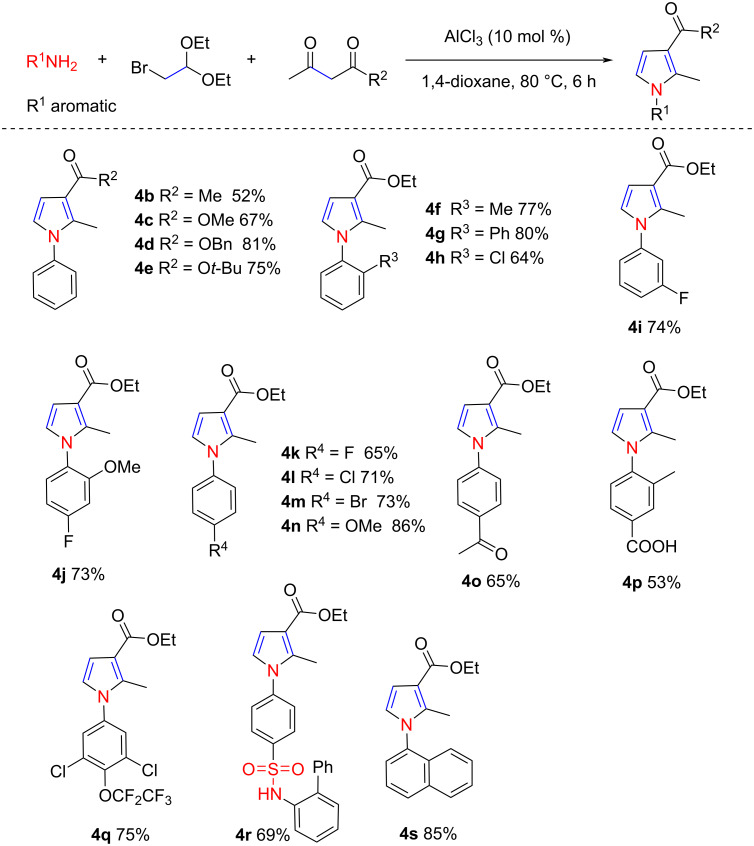
Substrate scope of the pyrrole synthesis.

The scope of this synthetic protocol for pyrroles was then investigated under the optimized reaction conditions. The substrate scope of the 1,3-dicarbonyl component was first examined (Scheme 2). Acetylacetone reacted smoothly with **1a** and **2a** to form **4b** in 52% yield. 1,3-Dicarbonyl compounds bearing an ester group readily participated in this reaction, affording the desired pyrroles **4c**–**e** in moderate to good yield. Notably, methyl 2-methyl-1-phenyl-1*H*-pyrrole-3-carboxylate (**4c**) is a key intermediate in the synthesis of a TRPM8 antagonist [43]. The substrate scope of the aromatic amine component was then examined, and the remarkable efficiency of our pyrrole synthesis was reflected by the tolerance of a broad range of functional groups attached to the aromatic amine. For example, anilines bearing methyl (in **4f**), phenyl (in **4g**), and halo functionalities (in **4h**–**m**) were readily compatible with the AlCl_3_ and 1,4-dioxane system. In these cases, the pyrrole products were isolated in moderate to good yield. The presence of an electron-donating group in the phenyl ring facilitated the progress of the reaction to some extent, resulting in **4n**. Anilines with electron-withdrawing groups, such as an acetyl or carboxy group, can also be used in the reaction, but the yields obtained for **4o** and **4p** were slightly inferior. Gratifyingly, a 3,5-dichloro-4-(1,1,2,2-tetrafluoroethoxy)aniline also participated smoothly in this reaction, and the expected product **4q** can be obtained in 75% yield. This fluorinated substituent on the aniline ring has been identified as the key precursor to access the insect-growth regulators [44]. It is noteworthy that the sulfonamide group persisted during this transformation, and the desired pyrrole product **4r** could be obtained in 69% yield. A high yield was also obtained when the phenyl group was replaced with a naphthyl group in **4s**. Subsequently, aliphatic primary amines and ammonia, such as benzylamine and *N*-butylamine, were also examined as nitrogen donors; however, no desired product was detected.

**Scheme 3:**
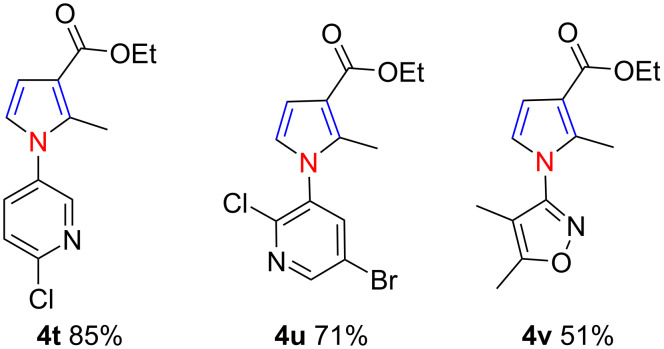
Synthesis of N-heterocyclic pyrroles.

Then, we attempted to synthesize pharmaceutically active N-heterocyclic pyrrole derivatives with the aid of this three-component reaction. To our great delight, the N-heterocyclic pyrrole skeletons **4t**–**v** were successfully synthesized in 51–85% yield by using our protocol (Scheme 3). It should be mentioned that the obtained *N*-pyridylpyrroles **4t** and **4u** are a class of very important intermediates for synthesizing the soluble guanylyl cyclase (sGC) activator. A reported method for accessing these similar scaffolds suffers from a low product yield and harsh reaction conditions (32%, 130 °C) [45–46].

**Scheme 4:**
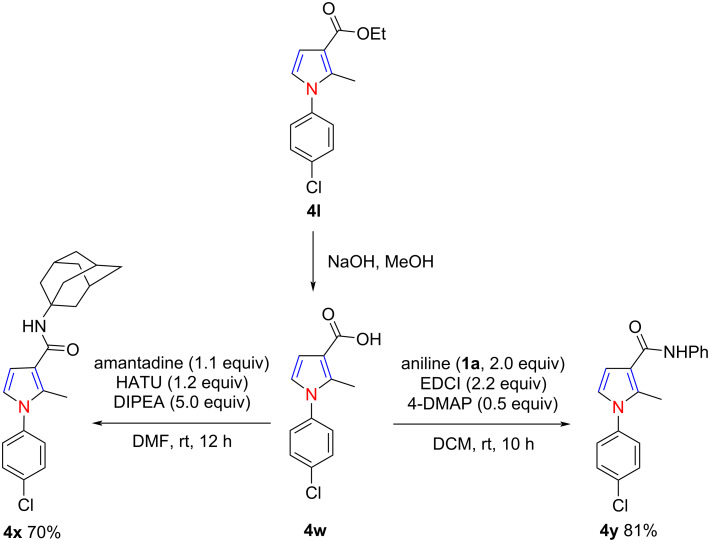
Direct synthesis of pyrrole-3-carboxamide derivatives.

One of the obtained *N*-aryl-4,5-unsubstituted pyrroles, **4l**, could undergo hydrolysis to form the (pyrrol-3-yl)carboxylic acid **4w**. The latter can react readily with aniline (**1a**) or amantadine in the presence of HATU or EDCI to form the multisubstituted pyrrole-3-carboxamide derivatives **4x** and **4y** (Scheme 4). These skeletons have been proven to be promising inhibitors for the production of cytokines [47].

**Scheme 5:**
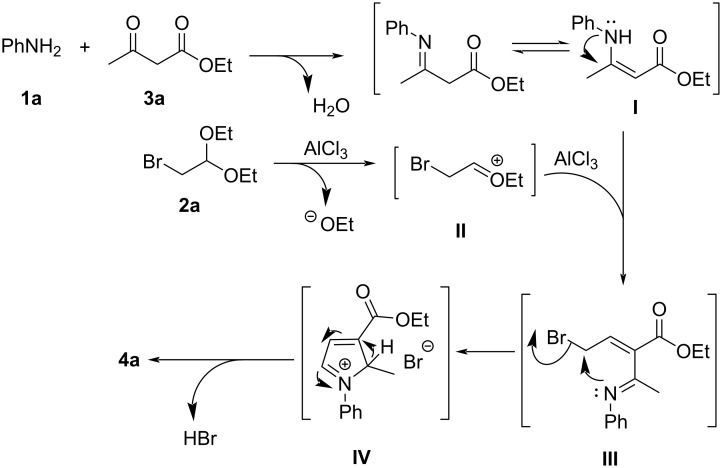
Plausible mechanism of the three-component reaction.

A plausible mechanism for the model reaction was proposed and is depicted in Scheme 5. Initially, a reaction of **1a** and **3a** occurred, providing an imine intermediate **I**, which tautomerized to the corresponding enamine form. Meanwhile, the activation of **2a** with AlCl_3_ allowed the formation of a carbocation intermediate **II**, which was then trapped by the enamine intermediate **I** to generate another intermediate **III**. Subsequently, **III** underwent an intramolecular electrophilic substitution to form the intermediate **IV**. Finally, **IV** underwent an elimination of HBr and a spontaneous aromatization to afford the pyrrole product **4a** [48–49].

**Scheme 6:**
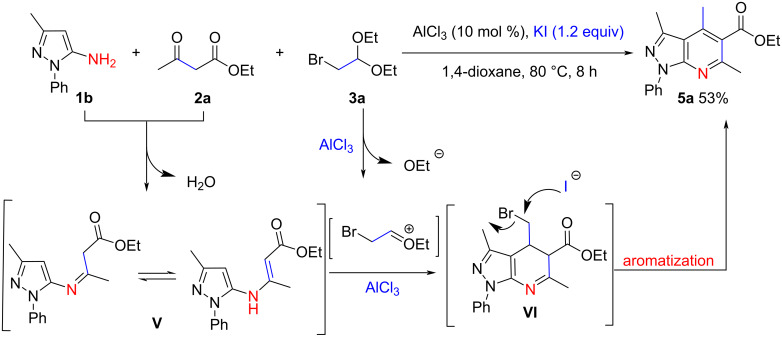
Synthesis of polysubstituted pyrazolo[3,4-b]pyridine derivatives.

Apart from the pyrrole synthesis, we also observed an unexpected reaction in which the pyrazolo[3,4-*b*]pyridine scaffold was constructed under analogous conditions. As shown in Scheme 6, In the presence of a catalytic amount of AlCl_3_ and 1.2 equiv of KI, 3-methyl-1-phenyl-1*H*-pyrazol-5-amine (**1b**) reacted smoothly with **2a** and **3a**, affording the polysubstituted pyrazolo[3,4-*b*]pyridine **5a** in 53% yield. The formation of an imine intermediate **V** may be involved in the reaction mechanism, and the enamine form of **V** contained two nucleophilic sites to react with a molecule of **3a**, which generated the intermediate **VI**. Finally, **VI** underwent an aromatization to produce **5a**. KI may play a key role in the last step, and we suspected that it can promote the removal of bromide. Although **5a** can theoretically be synthesized through a three-component reaction of **1b**, **2a**, and an appropriate aldehyde, for example, acetaldehyde [50–51] owing to the insufficient reactivity of aliphatic aldehydes, the reaction in Scheme 6 should be a wise choice for the synthesis of products similar in type to **5a**. This point deserves further investigation.

## Conclusion

In summary, an efficient and practical one-pot multicomponent reaction of (hetero)arylamines with α-bromoacetaldehyde acetal (**2a**) and 1,3-dicarbonyl compounds was developed by using AlCl_3_ as a catalyst. The developed chemistry is also successful for the synthesis of functionalized pyrazolo[3,4-*b*]pyridine derivatives. This study offered a complementary method to construct pyrrole scaffolds through [1 + 2 + 2] annulation, and thus enriching the product diversity of the pyrrole derivatives.

## Supporting Information

File 1 Experimental procedures and copies of NMR spectra.
