# Supplementary material for: Three-component reactions of aromatic amines, 1,3-dicarbonyl compounds, and α-bromoacetaldehyde acetal to access N-(hetero)aryl-4,5-unsubstituted pyrroles
Source: Beilstein J Org Chem. 2020 Nov 30;16:2920–8. doi: 10.3762/bjoc.16.241 (PMC7722624; doi:10.3762/bjoc.16.241)
Supplement: File 1 — Experimental procedures and copies of NMR spectra. [file Beilstein_J_Org_Chem-16-2920-s001.pdf]

BEILSTEIN JOURNAL OF ORGANIC CHEMISTRY

Supporting Information

for

Three-component reactions of aromatic amines, 1,3-dicarbonyl compounds, and α-bromoacetaldehyde acetal to access *N*-(hetero)aryl-4,5-unsubstituted pyrroles

Wenbo Huang, Kaimei Wang, Ping Liu, Minghao Li, Shaoyong Ke and Yanlong Gu

Beilstein J. Org. Chem. **2020,** *16,* 2920–2928. doi:10.3762/bjoc.16.241

Experimental procedures and copies of NMR spectra

License and Terms: This is a supporting information file under the terms of the Creative Commons Attribution License (https://creativecommons.org/licenses/by/4.0). Please note that the reuse, redistribution and reproduction in particular requires that the author(s) and source are credited and that individual graphics may be subject to special legal provisions.
The license is subject to the *Beilstein Journal of Organic Chemistry* terms and conditions: (https://www.beilstein-journals.org/bjoc/terms)

Table of contents

1. General remarks S1

2. Experimental section..S1

3. Characterization data of the compounds S3

4. References..S11

5. ^{1}H NMR and ^{13}C NMR spectra..S12

1. General remarks

Methyl acetoacetate, ethyl acetoacetate, and acetylacetone were purchased from Sinopharm Chemical Reagent Co., Ltd. Aniline (**1a**), bromoacetaldehyde diethyl acetal, and benzyl acetoacetate were purchased from Accela Chem Bio Co., Ltd. 2-Aminodiphenyl, 1-amino-2-methylbenzene, *p*-anisidine, and 2-chloroaniline were purchased from Accela Chem Bio Co., Ltd. 3-Fluoroaniline, 4-fluoro-2-methoxyaniline, 4-fluoroaniline, 4-chloroaniline, 4-bromoaniline, 5-amino-2-chloropyridine, and 5-bromo-2-chloropyridine were purchased from Energy Chemical Company. 4-Aminoacetophenone, 4-amino-3-methylbenzoic acid, 1-naphthylamine, and 3-amino-4,5-dimethylisoxazole were purchased from Alfa Aesar Chemical Company. ^{1}H and ^{13}C NMR spectra were recorded on a Bruker AV-400 or Bruker AV-600 device. Chemical shifts are expressed in ppm relative to Me_4Si in $CDCl_3$ or DMSO-d_6. High-resolution mass spectra were obtained on a Bruker Compass Data Analysis 4.0 spectrometer.

2. Experimental section

Typical procedure for the synthesis of *N*-(hetero)aryl-4,5-unsubstituted pyrrole derivatives

The reactions were conducted in a 10 mL V-type flask equipped with magnetic stirring. In a typical reaction, aniline (**1a**, 0.5 mmol) was mixed with **2a** (0.6 mmol), **3a** (0.6 mmol) and $AlCl_3$ (10 mol %) in acetonitrile (1.0 mL). The mixture was then stirred at 80 °C for 6 h. After the reaction (detected by TLC), the product was obtained by isolation with silica column chromatography (eluting solution:

petroleum ether/ethyl acetate 20:1, v/v). The tests for the substrate scope were all performed with an analogous procedure.

Procedure for the synthesis of 7a [1,2].

To a solution of [1,1'-biphenyl]-2-amine (1.0 equiv) in dichloromethane, pyridine (1.1 equiv) was added at 0 °C. Subsequently, a solution of 4-acetamidobenzene sulfonyl chloride (1.1 equiv) in dichloromethane was added dropwise at 0 °C and further stirred at room temperature overnight. The completion of the reaction was monitored by TLC, and the reaction mixture was quenched with saturated $NaHCO_3$, followed by extraction with DCM. The separated organic layer was dried over Na_2SO_4. Then, the mixture was concentrated under reduced pressure, and the residual mixture was subjected to an isolation by silica column chromatography (eluting solution: petroleum ether/ethyl acetate 10:1, v/v). The product **6a** was obtained in 80% yield. The obtained product **6a** was dissolved in 20% NaOH solution and the reaction mixtures were heated to reflux in an oil bath for 2 h. Then, the reaction solution was quenched with 2 M hydrochloric acid to pH 7.4, resulting in a large amount of a white solid forming. The crude product was obtained and washed with 5 × 10 mL water, then dried overnight in a vacuum desiccator, and the product **7a** was obtained in 82% yield.

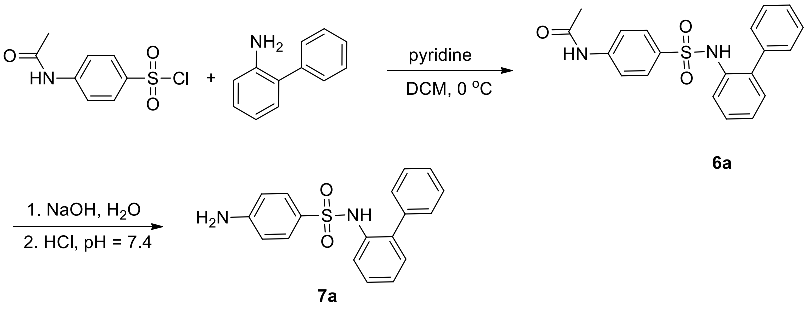

3. Characterization data of the compounds

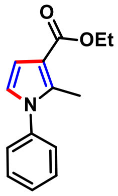

Ethyl 2-methyl-1-phenyl-1*H*-pyrrole-3-carboxylate (**4a**) [3]: yellow oil, ^{1}H NMR (600 MHz, $CDCl_3$, TMS, 25 °C) δ = 7.36 (t, J = 7.5 Hz, 2H), 7.32 – 7.27 (m, 1H), 7.16 (d, J = 7.7 Hz, 2H), 6.57 (q, J = 3.0 Hz, 2H), 4.20 (q, J = 7.1 Hz, 2H), 2.35 (s, 3H), 1.26 (t, J = 7.1 Hz, 3H) ppm. ^{13}C NMR (150 MHz, $CDCl_3$, 25 °C) δ = 165.6, 139.2, 136.0, 129.3, 128.0, 126.3, 121.3, 113.3, 110.2, 59.4, 14.6, 12.2 ppm. HRMS (TOF, ESI): m/z calcd for $C_{14}H_{16}NO_2$, [M + H]$^+$ 230.1181, found 230.1185.

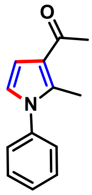

1-(2-Methyl-1-phenyl-1*H*-pyrrol-3-yl)ethanone (**4b**) [4]: yellow oil, ^{1}H NMR (600 MHz, $CDCl_3$, TMS, 25 °C) δ = 7.50 – 7.46 (m, 2H), 7.44 – 7.41 (m, 1H), 7.28 – 7.26 (m, 2H), 6.68 (d, J = 3.1 Hz, 1H), 6.62 (d, J = 3.1 Hz, 1H), 2.46 (s, 3H), 2.46 (s, 3H) ppm. ^{13}C NMR (150 MHz, $CDCl_3$, 25 °C) δ = 195.4, 139.0, 135.7, 129.5, 128.3, 126.4, 122.1, 121.3, 110.6, 28.8, 12.9 ppm. HRMS (TOF, ESI): m/z calcd for $C_{13}H_{14}NO$, [M + H]$^+$ 200.1075, found 200.1077.

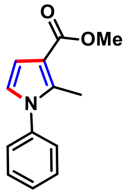

Methyl 2-methyl-1-phenyl-1*H*-pyrrole-3-carboxylate (**4c**): yellow oil, ^{1}H NMR (600 MHz, $CDCl_3$, TMS, 25 °C) δ = 7.49 – 7.46 (m, 2H), 7.43 –7.40 (m, 1H), 7.28 – 7.26 (m, 2H), 6.67 (d, J = 3.1 Hz, 1H), 6.65 (d, J = 3.1 Hz, 1H), 3.83 (s, 3H), 2.44 (s, 3H) ppm. ^{13}C NMR (150 MHz, $CDCl_3$, 25 °C) δ = 166.0, 139.2, 136.2, 129.3, 128.0, 126.3, 121.4, 113.0, 110.1, 50.9, 12.2 ppm. HRMS (TOF, ESI): m/z calcd for $C_{13}H_{14}NO_2$, [M + H]$^+$ 216.1025, found 216.1028.

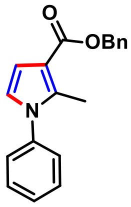

Benzyl 2-methyl-1-phenyl-1*H*-pyrrole-3-carboxylate (**4d**): yellow oil, ^{1}H NMR (600 MHz, $CDCl_3$, TMS, 25 °C) δ = 7.47 – 7.43 (m, 4H), 7.40 (t, J = 7.4 Hz, 1H), 7.36 (t, J = 7.5 Hz,

2H), 7.30 (t, J = 7.3 Hz, 1H), 7.25 (dd, J = 12.7, 5.2 Hz, 2H), 6.71 (d, J = 3.1 Hz, 1H), 6.66 (d, J = 3.1 Hz, 1H), 5.31 (s, 2H), 2.45 (s, 3H) ppm. ^{13}C NMR (150 MHz, $CDCl_3$, 25 °C) δ = 165.3, 139.2, 137.1, 136.5, 129.3, 128.5, 128.1, 128.0, 127.9, 126.3, 121.5, 113.0, 110.4, 65.3, 12.3 ppm. HRMS (TOF, ESI): m/z calcd for $C_{19}H_{18}NO_2$, $[M + H]^+$ 292.1338, found 292.1340.

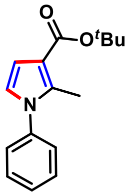

tert-Butyl 2-methyl-1-phenyl-1*H*-pyrrole-3-carboxylate (**4e**) [5]: yellow oil, ^{1}H NMR (600 MHz, $CDCl_3$, TMS, 25 °C) δ = 7.48 – 7.44 (m, 2H), 7.42 – 7.38 (m, 1H), 7.27 – 7.25 (m, 2H), 6.65 (d, J = 3.1 Hz, 1H), 6.63 (d, J = 3.1 Hz, 1H), 2.42 (s, 3H), 1.58 (s, 9H) ppm. ^{13}C NMR (150 MHz, $CDCl_3$, 25 °C) δ = 165.1, 139.3, 135.4, 129.3, 127.9, 126.3, 121.0, 114.9, 110.4, 79.4, 28.5, 12.3 ppm. HRMS (TOF, ESI): m/z calcd for $C_{16}H_{20}NO_2$, $[M + H]^+$ 258.1494, found 258.1495.

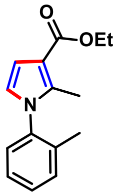

Ethyl 2-methyl-1-(*o*-tolyl)-1*H*-pyrrole-3-carboxylate (**4f**) [3]: yellow oil, ^{1}H NMR (600 MHz, $CDCl_3$, TMS, 25 °C) δ = 7.37 – 7.32 (m, 2H), 7.28 (t, J = 7.3 Hz, 1H), 7.16 (d, J = 7.6 Hz, 1H), 6.67 (t, J = 7.9 Hz, 1H), 6.52 (d, J = 2.9 Hz, 1H), 4.30 (q, J = 7.0 Hz, 2H), 2.01 (s, 3H), 1.37 (t, J = 7.1 Hz, 3H) ppm. ^{13}C NMR (150 MHz, $CDCl_3$, 25 °C) δ = 165.7, 138.2, 136.5, 136.0, 130.9, 128.9, 127.9, 126.7, 120.9, 112.5, 109.9, 59.4, 17.2, 14.6, 11.6 ppm. HRMS (TOF, ESI): m/z calcd for $C_{15}H_{18}NO_2$, $[M + H]^+$ 244.1332, found 244.1335.

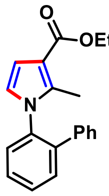

Ethyl 1-([1,1'-biphenyl]-2-yl)-2-methyl-1*H*-pyrrole-3-carboxylate (**4g**): yellow oil ^{1}H NMR (600 MHz, $CDCl_3$, TMS, 25 °C) δ = 7.50 (dtd, J = 8.9, 7.7, 1.5 Hz, 2H), 7.42 (td, J = 7.5, 1.8 Hz, 1H), 7.27 (dd, J = 7.8, 1.0 Hz, 1H), 7.25 – 7.20 (m, 3H), 7.06 – 7.02 (m, 2H), 6.56 (d, J = 3.1 Hz, 1H), 6.49 (d, J = 3.1 Hz, 1H), 4.22 (qd, J = 7.1, 3.2 Hz, 2H), 2.10 (s, 3H), 1.30 (t, J = 7.1 Hz, 3H) ppm. ^{13}C NMR (150 MHz, $CDCl_3$, 25 °C) δ = 165.6, 139.6, 138.0, 136.8, 136.6, 131.0, 129.0, 128.6, 128.5, 128.2, 127.6, 121.7, 112.5, 110.1, 59.3, 14.5, 11.8 ppm. HRMS (TOF, ESI): m/z calcd for $C_{20}H_{20}NO_2$, $[M + H]^+$ 306.1494, found 306.1498.

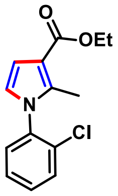

Ethyl 1-(2-chlorophenyl)-2-methyl-1*H*-pyrrole-3-carboxylate (**4h**) [3]: yellow oil, ^{1}H NMR (600 MHz, $CDCl_3$, TMS, 25 °C) δ = 7.53 (dd, J = 7.8, 1.1 Hz, 1H), 7.42 – 7.36 (m, 2H), 7.30 (dd, J = 7.6, 1.6 Hz, 1H), 6.70 (d, J = 3.1 Hz, 1H), 6.55 (d, J = 3.1 Hz, 1H), 4.30 (q, J = 7.1 Hz, 2H), 2.31 (s, 3H), 1.36 (t, J = 7.1 Hz, 3H) ppm. ^{13}C NMR (150 MHz, $CDCl_3$, 25 °C) δ = 165.5, 137.0, 136.8, 132.7, 130.4, 130.2, 129.6, 127.7, 121.1, 112.9, 110.3, 59.5, 14.6, 11.6 ppm. HRMS (TOF, ESI): m/z calcd for $C_{14}H_{15}ClNO_2$, $[M + H]^+$ 264.0791, found 264.0792.

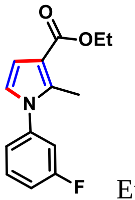

Ethyl 1-(3-fluorophenyl)-2-methyl-1*H*-pyrrole-3-carboxylate (**4i**): yellow oil, ^{1}H NMR (600 MHz, $CDCl_3$, TMS, 25 °C) δ = 7.44 (td, J = 8.1, 6.3 Hz, 1H), 7.12 (tdd, J = 8.4, 2.4, 0.7 Hz, 1H), 7.08 (dd, J = 7.9, 1.0 Hz, 1H), 7.01 (dt, J = 9.3, 2.2 Hz, 1H), 6.67 (dd, J = 7.3, 3.1 Hz, 2H), 4.30 (q, J = 7.1 Hz, 2H), 2.47 (s, 3H), 1.36 (t, J = 7.1 Hz, 3H) ppm. ^{13}C NMR (150 MHz, $CDCl_3$, 25 °C) δ = 165.4, 162.7 (d, J = 247.5 Hz), 140.6 (d, J = 9.8 Hz), 135.8, 130.5 (d, J = 9.2 Hz), 122.0 (d, J = 3.2 Hz), 121.1, 115.0 (d, J = 21.0 Hz), 113.8 (d, J = 24.0 Hz), 113.7, 110.6, 59.5, 14.5, 12.2 ppm. ^{19}F NMR (565 MHz, $CDCl_3$, 25 °C) δ = -110.8 ppm. HRMS (TOF, ESI): m/z calcd for $C_{14}H_{15}FNO_2$, $[M + H]^+$ 248.1087, found 248.1085.

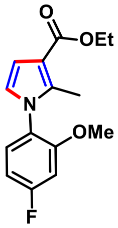

Ethyl 1-(4-fluoro-2-methoxyphenyl)-2-methyl-1*H*-pyrrole-3-carboxylate (**4j**): yellow oil, ^{1}H NMR (600 MHz, $CDCl_3$, TMS, 25 °C) δ = 7.15 (dd, J = 8.5, 6.2 Hz, 1H), 6.78 – 6.70 (m, 2H), 6.66 (d, J = 3.0 Hz, 1H), 6.52 (d, J = 3.0 Hz, 1H), 4.29 (q, J = 7.1 Hz, 2H), 3.78 (s, 3H), 2.29 (s, 3H), 1.35 (t, J = 7.1 Hz, 3H) ppm. ^{13}C NMR (150 MHz, $CDCl_3$, 25 °C) δ = 165.6, 163.3 (d, J = 246.0 Hz), 156.2 (d, J = 10.6 Hz), 137.6, 129.7 (d, J = 10.4 Hz), 123.9 (d, J = 3.4 Hz), 121.6, 112.5, 109.9, 107.1 (d, J = 22.7 Hz), 100.3 (d, J = 27.2 Hz), 59.4, 56.0, 14.6, 11.6 ppm. ^{19}F NMR (565 MHz, $CDCl_3$, 25 °C) δ = -109.0 ppm. HRMS (TOF, ESI): m/z calcd for $C_{15}H_{17}FNO_3$, $[M + H]^+$ 278.1192, found 278.1195.

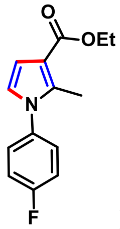

Ethyl 1-(4-fluorophenyl)-2-methyl-1*H*-pyrrole-3-carboxylate (**4k**): yellow oil, ^{1}H NMR (600 MHz, $CDCl_3$, TMS, 25 °C) δ = 7.27 – 7.23 (m, 2H) , 7.18 – 7.14 (m, 2H), 6.66 (d, *J* = 3.1 Hz, 1H), 6.63 (d, *J* = 3.1 Hz, 1H), 4.30 (q, *J* = 7.1 Hz, 2H), 2.42 (s, 3H), 1.36 (t, *J* = 7.1 Hz, 3H) ppm. ^{13}C NMR (150 MHz, $CDCl_3$, 25 °C) δ = 165.5, 162.5 (d, *J* = 246.0 Hz), 136.1, 135.2 (d, *J* = 3.1 Hz), 128.1(d, *J* = 8.7 Hz), 121.4, 116.2 (d, *J* = 22.5 Hz), 113.4, 110.3, 59.5, 14.6, 12.1 ppm. ^{19}F NMR (565 MHz, $CDCl_3$, 25 °C) δ = -113.2 ppm. HRMS (TOF, ESI): m/z calcd for $C_{14}H_{15}FNO_2$, [M + H]$^+$ 248.1087, found 248.1084.

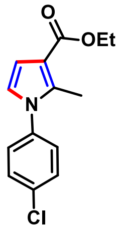

Ethyl 1-(4-chlorophenyl)-2-methyl-1*H*-pyrrole-3-carboxylate (**4l**) [3]: white solid, mp: 57 – 59 °C, ^{1}H NMR (600 MHz, $CDCl_3$, TMS, 25 °C) δ = 7.46 – 7.44 (m, 1H), 7.44 – 7.43 (m, 1H), 7.23 – 7.22 (m, 1H), 7.21 – 7.19 (m, 1H), 6.67 (d, *J* = 3.1 Hz, 1H), 6.64 (d, *J* = 3.1 Hz, 1H), 4.30 (q, *J* = 7.1 Hz, 2H), 2.43 (s, 3H), 1.36 (t, *J* = 7.1 Hz, 3H) ppm. ^{13}C NMR (150 MHz, $CDCl_3$, 25 °C) δ = 165.5, 137.7, 135.9, 133.9, 129.5, 127.5, 121.2, 113.7, 110.5, 77.3, 77.1, 59.5,14.6, 12.2 ppm. HRMS (TOF, ESI): m/z calcd for $C_{14}H_{15}ClNO_2$, [M + H]$^+$ 264.0791, found 264.0792.

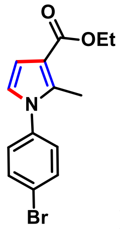

Ethyl 1-(4-bromophenyl)-2-methyl-1*H*-pyrrole-3-carboxylate (**4m**): yellow solid, 59 – 61 °C, ^{1}H NMR (600 MHz, $CDCl_3$, TMS, 25 °C) δ = 7.59 (d, *J* = 8.5 Hz, 2H), 7.15 (d, *J* = 8.5 Hz, 2H), 6.67 (d, *J* = 3.1 Hz, 1H), 6.63 (d, *J* = 3.1 Hz, 1H), 4.29 (q, *J* = 7.1 Hz, 2H), 2.43 (s, 3H), 1.36 (t, *J* = 7.1 Hz, 3H) ppm. ^{13}C NMR (150 MHz, $CDCl_3$, 25 °C) δ = 165.4, 138.2, 135.8, 132.5, 127.8, 121.8, 121.1, 113.7, 110.6, 59.5, 14.6, 12.2 ppm. HRMS (TOF, ESI): m/z calcd for $C_{14}H_{15}BrNO_2$, [M + H]$^+$ 308.0286, found 308.0289.

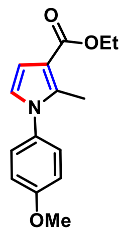

Ethyl 1-(4-methoxyphenyl)-2-methyl-1*H*-pyrrole-3-carboxylate (**4n**): yellow oil, ^{1}H NMR (600 MHz, $CDCl_3$, TMS, 25 °C) δ = 7.20 – 7.16 (m, 2H), 6.98 – 6.95 (m, 2H), 6.64 (d, J = 3.0 Hz, 1H), 6.62 (d, J = 3.1 Hz, 1H), 4.29 (q, J = 7.1 Hz, 2H), 3.85 (s, 3H), 2.40 (s, 3H), 1.36 (t, J = 7.1 Hz, 3H) ppm. ^{13}C NMR (150 MHz, $CDCl_3$, 25 °C) δ = 165.6, 159.2, 136.3, 132.1, 127.5, 121.5, 114.4, 112.9, 109.8, 59.4, 55.6, 14.6, 12.1 ppm. HRMS (TOF, ESI): m/z calcd for $C_{15}H_{18}NO_3$, $[M + H]^+$ 260.1287, found 260.1285.

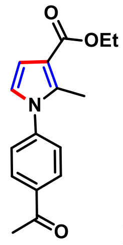

Ethyl 1-(4-acetylphenyl)-2-methyl-1*H*-pyrrole-3-carboxylate (**4o**): yellow solid, 55 – 57 °C, ^{1}H NMR (600 MHz, $CDCl_3$, TMS, 25 °C) δ = 8.08 (d, J = 8.5 Hz, 2H), 7.39 (d, J = 8.5 Hz, 2H), 6.72 – 6.69 (m, 2H), 4.30 (q, J = 7.1 Hz, 2H), 2.66 (s, 3H), 2.49 (s, 3H), 1.37 (t, J = 7.1 Hz, 3H) ppm. ^{13}C NMR (150 MHz, $CDCl_3$, 25 °C) δ = 196.8, 165.4, 143.0, 136.2, 135.7, 129.5, 126.0, 121.0, 114.3, 110.9, 59.6, 26.7, 14.5, 12.4 ppm. HRMS (TOF, ESI): m/z calcd for $C_{16}H_{18}NO_3$, $[M + H]^+$ 272.1287, found 272.1288.

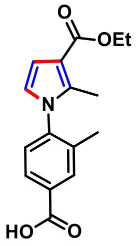

4-(3-(Ethoxycarbonyl)-2-methyl-1*H*-pyrrol-1-yl)-3-methylbenzoic acid (**4p**): yellow solid, 102 – 104 °C, ^{1}H NMR (600 MHz, $CDCl_3$, TMS, 25 °C) δ = 10.44 (s, 1H), 8.10 (s, 1H), 8.04 (dd, J = 8.1, 1.5 Hz, 1H), 7.29 (d, J = 8.1 Hz, 1H), 6.73 (d, J = 3.1 Hz, 1H), 6.54 (d, J = 3.1 Hz, 1H), 4.32 (q, J = 7.1 Hz, 2H), 2.28 (s, 3H), 2.10 (s, 3H), 1.38 (t, J = 7.1 Hz, 3H) ppm. ^{13}C NMR (150 MHz, $CDCl_3$, 25 °C) δ = 171.1, 165.8, 142.9, 136.5, 136.3, 132.9, 129.9, 128.7, 128.3, 120.6, 113.0, 110.6, 59.7, 20.9, 14.5, 11.7 ppm. HRMS (TOF, ESI): m/z calcd for $C_{16}H_{18}NO_4$, $[M + H]^+$ 288.1236, found 288.1239.

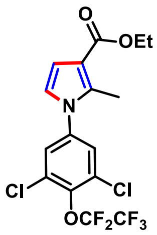

Ethyl 1-(3,5-dichloro-4-(perfluoroethoxy)phenyl)-2-methyl-1*H*-pyrrole-3-carboxylate (**4q**): yellow oil, ^{1}H NMR (600 MHz, $CDCl_3$, TMS, 25 °C) δ = 7.36 (s, 2H), 6.69 (d, *J* = 3.1 Hz, 1H), 6.65 (d, *J* = 3.1 Hz, 1H), 4.30 (q, *J* = 7.1 Hz, 2H), 2.50 (s, 3H), 1.36 (t, *J* = 7.1 Hz, 3H) ppm. ^{13}C NMR (150 MHz, $CDCl_3$, 25 °C) δ = 165.1, 141.5, 138.5, 135.7, 131.4, 126.9, 120.9, 117.1 (tt, *J* = 279.0, 28.8 Hz), 114.6, 111.3, 107.6 (tt, *J* = 252.7, 39.7 Hz), 59.7, 14.5, 12.2. ^{19}F NMR (565 MHz, $CDCl_3$, 25 °C) δ = -86.15 (t, *J* = 6.0 Hz), -136.18 (t, *J* = 5.8 Hz) ppm. HRMS (TOF, ESI): m/z calcd for $C_{16}H_{13}Cl_2F_5NO_3$, $[M + H]^+$ 432.0193, found 432.0196.

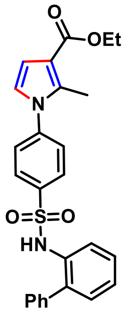

Ethyl 1-(4-(*N*-([1,1'-biphenyl]-2-yl)sulfamoyl)phenyl)-2-methyl-1*H*-pyrrole-3-carboxylate (**4r**): yellow oil, ^{1}H NMR (600 MHz, $CDCl_3$, TMS, 25 °C) δ = 7.74 (d, *J* = 8.1 Hz, 1H), 7.63 (d, *J* = 8.4 Hz, 2H), 7.37 (dd, *J* = 17.8, 7.0 Hz, 4H), 7.29 (d, *J* = 8.4 Hz, 2H), 7.21 (t, *J* = 7.4 Hz, 1H), 7.14 (dd, *J* = 7.4, 1.0 Hz, 1H), 6.91 – 6.86 (m, 2H), 6.75 (s, 1H), 6.71 (d, *J* = 3.1 Hz, 1H), 6.67 (d, *J* = 3.1 Hz, 1H), 4.30 (q, *J* = 7.1 Hz, 2H), 2.47 (s, 3H), 1.36 (t, *J* = 7.1 Hz, 3H) ppm. ^{13}C NMR (150 MHz, $CDCl_3$, 25 °C) δ = 165.3, 142.9, 138.4, 137.2, 135.5, 134.6, 133.1, 130.4, 129.2, 128.9, 128.7, 128.4, 128.3, 126.3, 125.7, 122.4, 120.9, 114.7, 111.3, 59.7, 14.5, 12.4 ppm. HRMS (TOF, ESI): m/z calcd for $C_{26}H_{25}N_2O_4S$, $[M + H]^+$ 461.1535, found 461.1537.

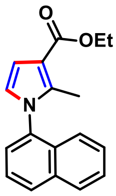

Ethyl 2-methyl-1-(naphthalen-1-yl)-1*H*-pyrrole-3-carboxylate (**4s**): yellow oil, ^{1}H NMR (600 MHz, $CDCl_3$, TMS, 25 °C) δ = 7.83 (dd, *J* = 11.4, 8.3 Hz, 2H), 7.45 – 7.41 (m, 2H), 7.35 (ddd, *J* = 8.1, 6.9, 1.2 Hz, 1H), 7.30 (dd, *J* = 7.2, 1.0 Hz, 1H), 7.15 (d, *J* = 8.2 Hz, 1H), 6.68 (d, *J* = 3.0 Hz, 1H), 6.59 (d, *J* = 3.0 Hz, 1H), 4.24 (q, *J* = 7.1 Hz, 2H), 2.15 (s, 3H), 1.28 (t, *J* = 7.1 Hz, 3H) ppm. ^{13}C NMR (150 MHz, $CDCl_3$, 25 °C) δ = 165.8, 137.7, 135.7, 134.1, 130.8, 129.3, 128.2, 127.5,

126.9, 125.3, 125.2, 122.9, 122.4, 112.8, 110.0, 59.5, 14.6, 11.8 ppm. HRMS (TOF, ESI): m/z calcd for $C_{18}H_{18}NO_2$, $[M + H]^+$ 280.1338, found 280.1336.

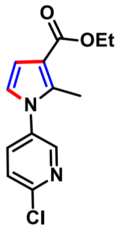

Ethyl 1-(6-chloropyridin-3-yl)-2-methyl-1*H*-pyrrole-3-carboxylate (**4t**): white solid, 65 – 67 °C, ^{1}H NMR (600 MHz, $CDCl_3$, TMS, 25 °C) δ = 8.38 (d, J = 2.6 Hz, 1H), 7.62 (dd, J = 8.4, 2.8 Hz, 1H), 7.53 – 7.47 (m, 1H), 6.71 (d, J = 3.1 Hz, 1H), 6.66 (d, J = 3.1 Hz, 1H), 4.30 (q, J = 7.1 Hz, 2H), 2.46 (s, 3H), 1.36 (t, J = 7.1 Hz, 3H) ppm. ^{13}C NMR (150 MHz, $CDCl_3$, 25 °C) δ = 165.1, 150.6, 147.3, 146.9, 136.2, 128.6, 124.8, 121.1, 114.6, 111.3, 59.7, 14.5, 12.0 ppm. HRMS (TOF, ESI): m/z calcd for $C_{13}H_{14}ClN_2O_2$, $[M + H]^+$ 265.0744, found 265.0747.

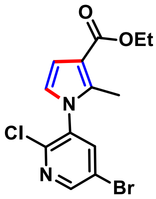

Ethyl 1-(5-bromo-2-chloropyridin-3-yl)-2-methyl-1*H*-pyrrole-3-carboxylate (**4u**): yellow oil, ^{1}H NMR (600 MHz, $CDCl_3$, TMS, 25 °C) δ = 8.57 (d, J = 2.3 Hz, 1H), 7.89 (d, J = 2.3 Hz, 1H), 6.71 (d, J = 3.2 Hz, 1H), 6.58 (d, J = 3.2 Hz, 1H), 4.30 (q, J = 7.0 Hz, 2H), 2.35 (s, 3H), 1.37 (t, J = 7.1 Hz, 3H) ppm. ^{13}C NMR (150 MHz, $CDCl_3$, 25 °C) δ = 165.0, 150.7, 148.5, 140.5, 136.7, 134.4, 120.9, 118.8, 114.0, 111.4, 59.7, 14.5, 11.7 ppm. HRMS (TOF, ESI): m/z calcd for $C_{13}H_{13}BrClN_2O_2$, $[M + H]^+$ 342.9849, found 343.9851.

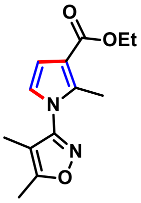

Ethyl 1-(4,5-dimethylisoxazol-3-yl)-2-methyl-1*H*-pyrrole-3-carboxylate (**4v**): yellow oil, ^{1}H NMR (600 MHz, $CDCl_3$, TMS, 25 °C) δ = 6.61 (d, J = 2.8 Hz, 1H), 6.56 (d, J = 2.8 Hz, 1H), 4.21 (q, J = 7.0 Hz, 2H), 2.40 (s, 3H), 2.34 (s, 3H), 1.77 (s, 3H), 1.28 (t, J = 7.1 Hz, 3H) ppm. ^{13}C NMR (150 MHz, $CDCl_3$, 25 °C) δ = 168.1, 165.2, 159.2, 136.8, 120.2, 114.3, 111.3, 107.0, 59.6, 14.5, 11.8, 11.5, 6.4 ppm. HRMS (TOF, ESI): m/z calcd for $C_{13}H_{17}N_2O_3$, $[M + H]^+$ 249.1239, found 249.1238.

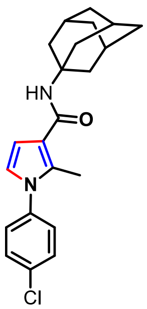

N-((3s,5s,7s)-Adamantan-1-yl)-1-(4-chlorophenyl)-2-methyl-1*H*-pyrrole-3-carboxamide (**4x**): yellow solid, 163 – 165 °C, ^{1}H NMR (600 MHz, $CDCl_3$, TMS, 25 °C) δ = 7.43 (d, *J* = 8.5 Hz, 2H), 7.20 (d, *J* = 8.5 Hz, 2H), 6.63 (d, *J* = 3.0 Hz, 1H), 6.31 (d, *J* = 3.0 Hz, 1H), 5.53 (s, 1H), 2.43 (s, 3H), 2.14 – 2.10 (m, 9H), 1.75 – 1.68 (m, 6H) ppm. ^{13}C NMR (150 MHz, $CDCl_3$, 25 °C) δ = 165.2, 137.8, 133.7, 133.4, 129.5, 127.5, 120.7, 117.4, 107.2, 51.8, 42.0, 36.5, 29.6, 12.0 ppm. HRMS (TOF, ESI): m/z calcd for $C_{22}H_{26}ClN_2O$, $[M + H]^+$ 369.1734, found 369.1738.

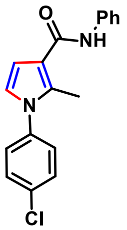

1-(4-Chlorophenyl)-2-methyl-*N*-phenyl-1*H*-pyrrole-3-carboxamide (**4y**): yellow solid, 152 – 154 °C, ^{1}H NMR (600 MHz, $CDCl_3$, TMS, 25 °C) δ = 7.61 (d, *J* = 7.8 Hz, 2H), 7.55 (s, 1H), 7.46 (d, *J* = 8.6 Hz, 2H), 7.35 (t, *J* = 7.9 Hz, 2H), 7.24 (d, *J* = 8.6 Hz, 2H), 7.11 (t, *J* = 7.4 Hz, 1H), 6.71 (d, *J* = 3.1 Hz, 1H), 6.48 (d, *J* = 2.1 Hz, 1H), 2.49 (s, 3H) ppm. ^{13}C NMR (150 MHz, $CDCl_3$, 25 °C) δ = 163.8, 138.4, 137.5, 134.9, 134.0, 129.6, 129.0, 127.5, 123.9, 121.3, 120.0, 116.4, 107.0, 12.1 ppm. HRMS (TOF, ESI): m/z calcd for $C_{18}H_{16}ClN_2O$, $[M + H]^+$ 311.0951, found 311.0955.

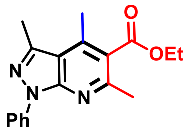

Ethyl 3,4,6-trimethyl-1-phenyl-1*H*-pyrazolo[3,4-b]pyridine-5-carboxylate (**5a**): colourless oil, ^{1}H NMR (600 MHz, $CDCl_3$, TMS, 25 °C) δ = 8.25 (d, *J* = 8.0 Hz, 2H), 7.49 (t, *J* = 7.9 Hz, 2H), 7.26 (t, *J* = 7.4 Hz, 1H), 4.45 (q, *J* = 7.1 Hz, 2H), 2.74 (s, 3H), 2.68 (s, 3H), 2.66 (s, 3H), 1.43 (t, *J* = 7.1 Hz, 3H) ppm. ^{13}C NMR (150 MHz, $CDCl_3$, 25 °C) δ = 169.3, 155.8, 150.1, 143.2, 140.9, 139.4, 129.0, 125.6, 124.2, 121.1, 114.2, 61.5, 24.0, 16.5, 15.7, 14.3 ppm. HRMS (TOF, ESI): m/z calcd for $C_{18}H_{20}N_3O_2$, $[M + H]^+$ 310.1556, found 310.1558.

4. References

(1) Kalsi, D.; Sundararaju, B., *Org. Lett.* **2015,** *17*, 6118–6121.

(2) Wang, L.; Li, L.; Zhou, Z. -H.; Jiang, Z. -Y.; You, Q. -D.; Xu, X. -L., *Eur. J. Med. Chem.* **2018,** *143*, 724–731.

(3) Zhang, Z.; Zhang, W.; Li, J.; Liu, Q.; Liu, T.; Zhang, G., *J. Org. Chem.* **2014**, *79*, 11226–11233.

(4) Yeh, J. -Y.; Coumar, M. S.; Shiao, H. -Y.; Lin, T. -J.; Lee, Y. -C.; Hung, H. -C.; Ko, S.; Kuo, F. -M.; Fang, M. -Y.; Huang, Y. -L.; Hsu, J. T. A.; Yeh, T. -K.; Shih, S. -R.; Chao, Y. -S.; Horng, J. -T.; Hsieh, H. -P., *ChemMedChem*. **2012**, *7*, 1546–1550.

(5) Tracey, M. R.; Hsung, R. P.; Lambeth, R. H., *Synthesis*. **2004**, 918–922.

5. ^{1}H NMR and ^{13}C NMR spectra

4a

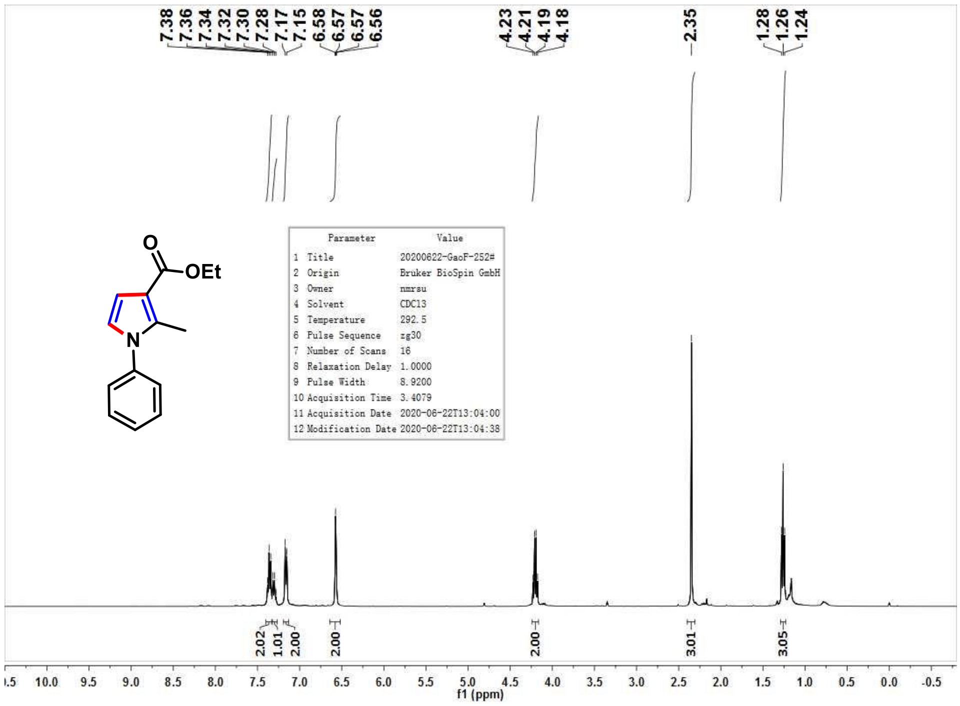

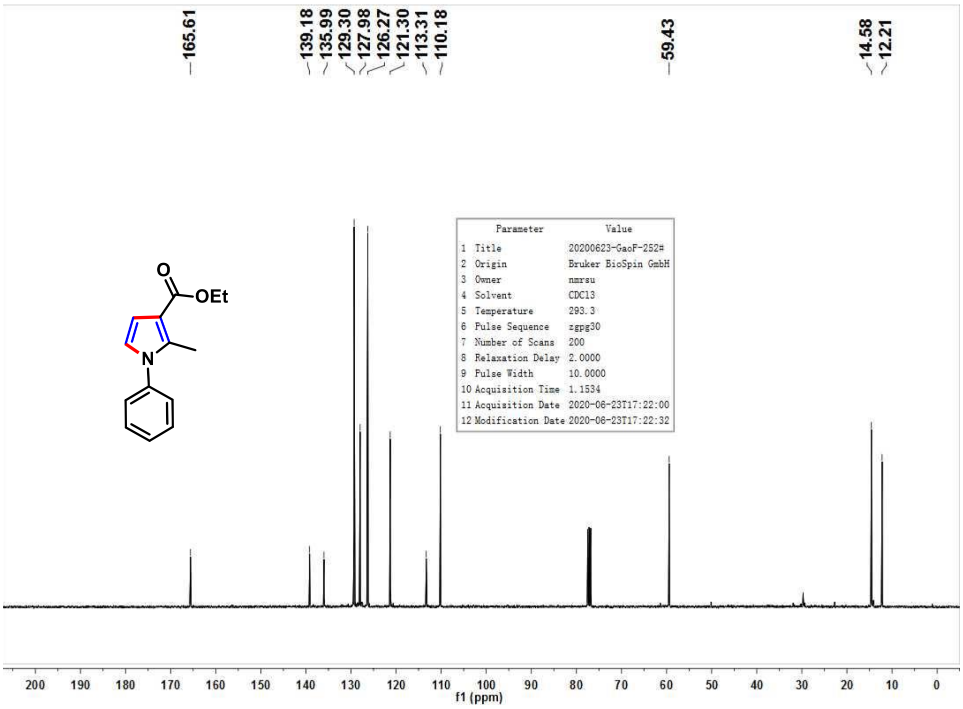

4b

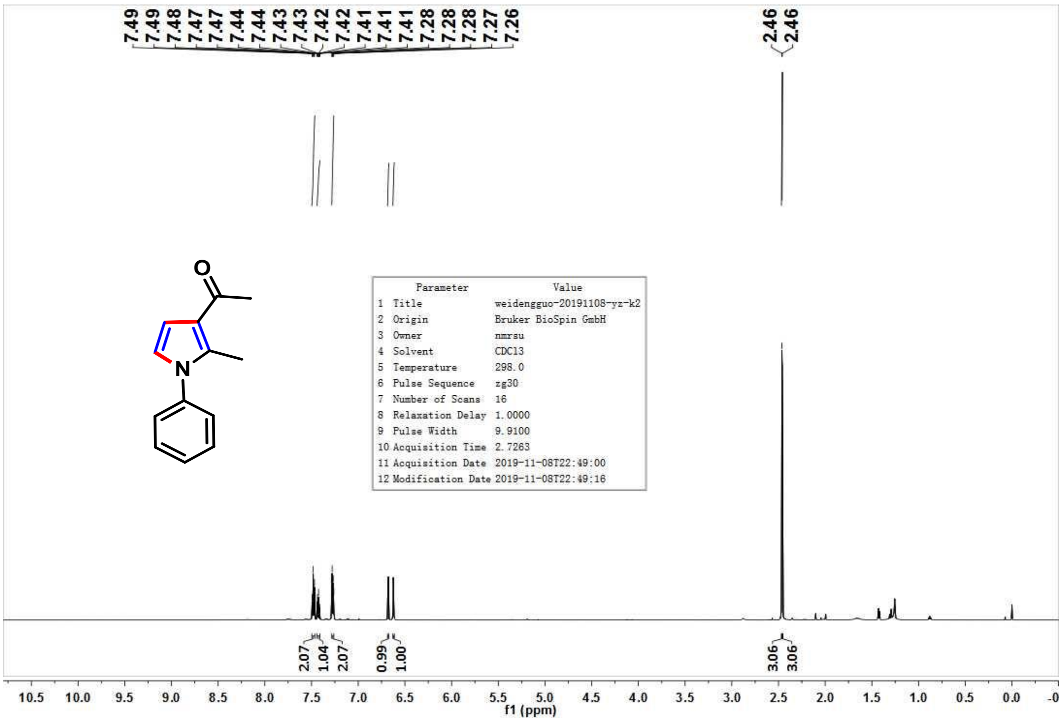
7.49
7.49
7.48
7.47
7.47
7.44
7.44
7.43
7.43
7.42
7.42
7.41
7.41
7.41
7.28
7.28
7.28
7.27
7.26
2.46
2.46
O
N
Parameter Value
1 Title weidengguo-20191108-yz-k2
2 Origin Bruker BioSpin GmbH
3 Owner nmrsu
4 Solvent CDCl3
5 Temperature 298.0
6 Pulse Sequence zg30
7 Number of Scans 16
8 Relaxation Delay 1.0000
9 Pulse Width 9.9100
10 Acquisition Time 2.7263
11 Acquisition Date 2019-11-08T22:49:00
12 Modification Date 2019-11-08T22:49:16
2.07
1.04
2.07
0.99
1.00
3.06
3.06
f1 (ppm)

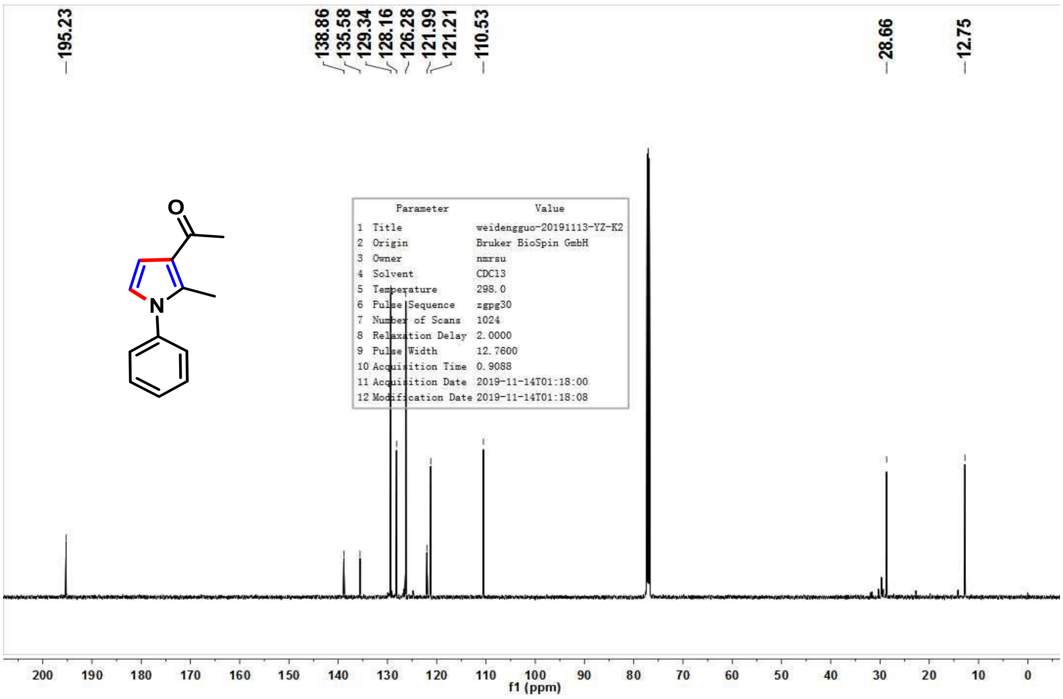
195.23
138.86
135.58
129.34
128.16
126.28
121.99
121.21
110.53
28.66
12.75
O
N
Parameter Value
1 Title weidengguo-20191113-YZ-K2
2 Origin Bruker BioSpin GmbH
3 Owner nmrsu
4 Solvent CDCl3
5 Temperature 298.0
6 Pulse Sequence zgpg30
7 Number of Scans 1024
8 Relaxation Delay 2.0000
9 Pulse Width 12.7600
10 Acquisition Time 0.9088
11 Acquisition Date 2019-11-14T01:18:00
12 Modification Date 2019-11-14T01:18:08
f1 (ppm)

4c

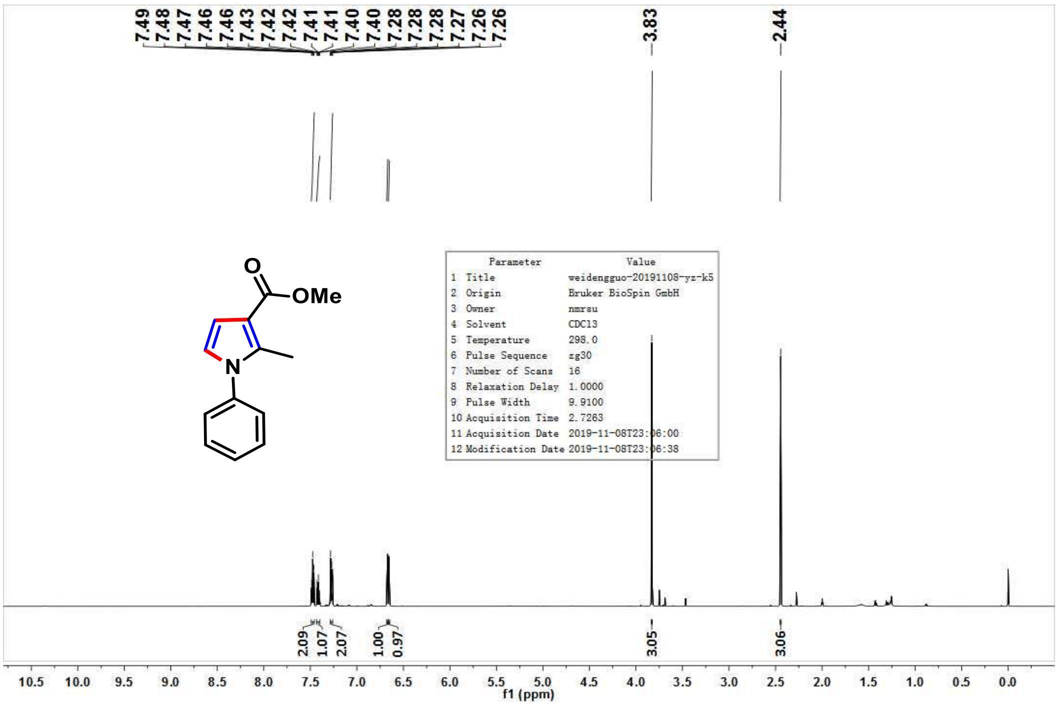

7.49 7.48 7.47 7.46 7.46 7.43 7.42 7.42 7.41 7.41 7.40 7.40 7.28 7.28 7.28 7.27 7.26 7.26
3.83
2.44
OMe
O
N
Parameter Value
1 Title weidengguo-20191108-yz-k5
2 Origin Bruker BioSpin GmbH
3 Owner nmrsu
4 Solvent CDCl3
5 Temperature 298.0
6 Pulse Sequence zg30
7 Number of Scans 16
8 Relaxation Delay 1.0000
9 Pulse Width 9.9100
10 Acquisition Time 2.7263
11 Acquisition Date 2019-11-08T23:06:00
12 Modification Date 2019-11-08T23:06:38
2.09 1.07 2.07 1.00 0.97 3.05 3.06
10.5 10.0 9.5 9.0 8.5 8.0 7.5 7.0 6.5 6.0 5.5 5.0 4.5 4.0 3.5 3.0 2.5 2.0 1.5 1.0 0.5 0.0
f1 (ppm)

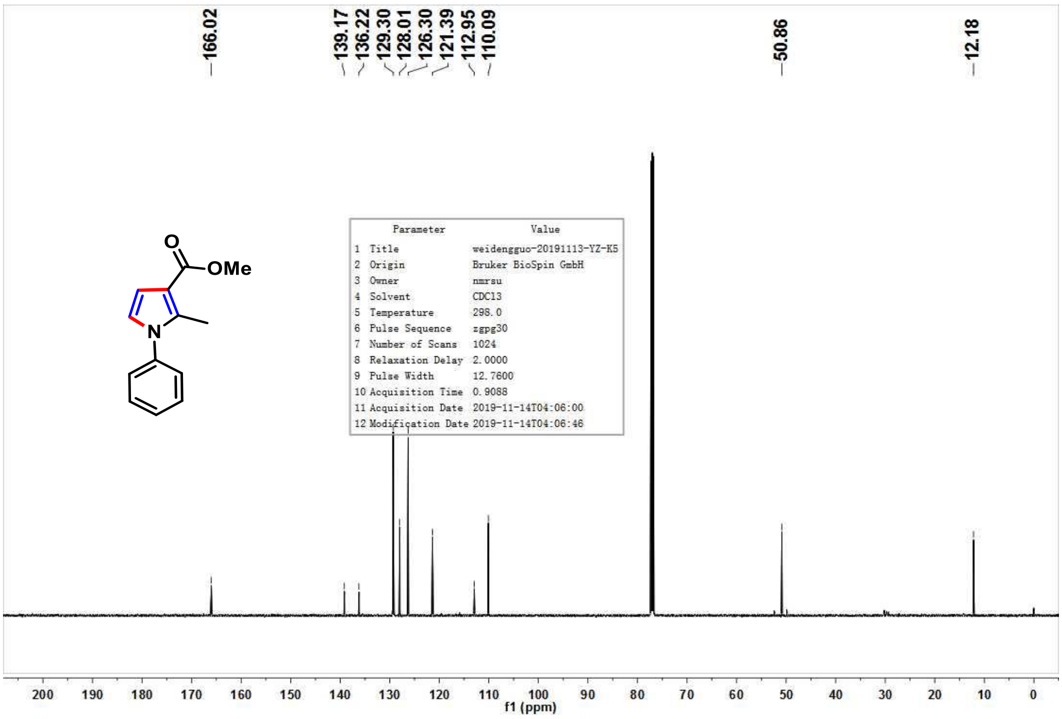

166.02
139.17 136.22 129.30 128.01 126.30 121.39 112.95 110.09
50.86
12.18
OMe
O
N
Parameter Value
1 Title weidengguo-20191113-YZ-K5
2 Origin Bruker BioSpin GmbH
3 Owner nmrsu
4 Solvent CDCl3
5 Temperature 298.0
6 Pulse Sequence zgpg30
7 Number of Scans 1024
8 Relaxation Delay 2.0000
9 Pulse Width 12.7600
10 Acquisition Time 0.9088
11 Acquisition Date 2019-11-14T04:06:00
12 Modification Date 2019-11-14T04:06:46
200 190 180 170 160 150 140 130 120 110 100 90 80 70 60 50 40 30 20 10 0
f1 (ppm)

4d

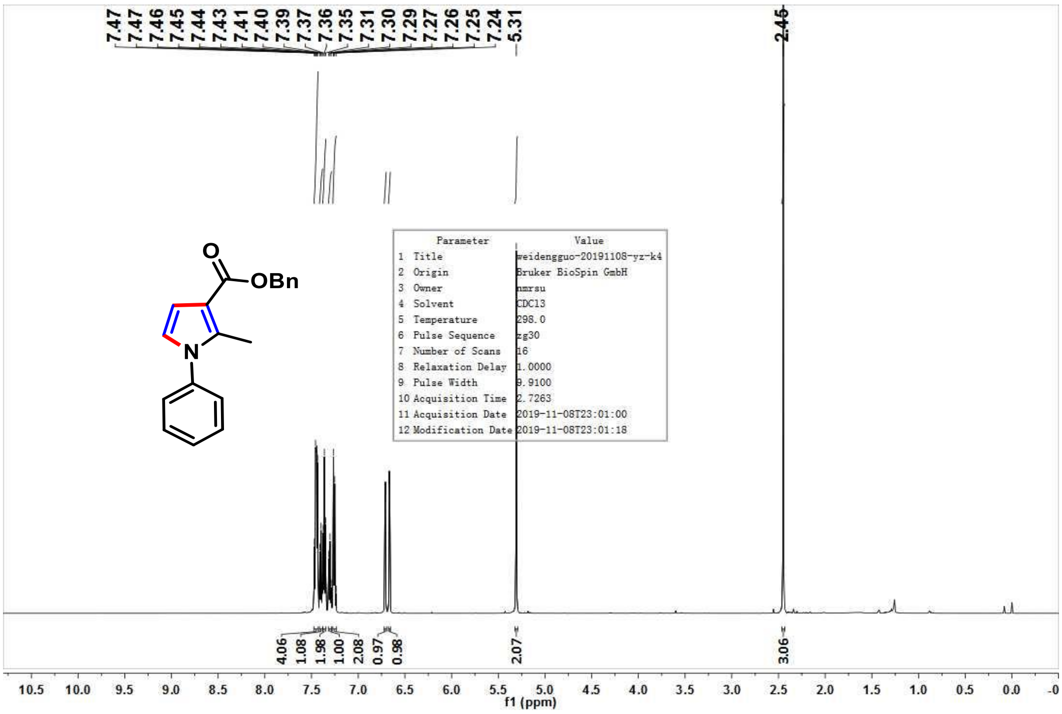

7.47 7.47 7.46 7.45 7.44 7.43 7.41 7.40 7.39 7.37 7.36 7.35 7.31 7.30 7.29 7.27 7.26 7.25 7.24 5.31
2.45
OBn
N
Parameter Value
1 Title weidengguo-20191108-yz-k4
2 Origin Bruker BioSpin GmbH
3 Owner nmrsu
4 Solvent CDCl3
5 Temperature 298.0
6 Pulse Sequence zg30
7 Number of Scans 16
8 Relaxation Delay 1.0000
9 Pulse Width 9.9100
10 Acquisition Time 2.7263
11 Acquisition Date 2019-11-08T23:01:00
12 Modification Date 2019-11-08T23:01:18
4.06 1.08 1.98 1.00 2.08 0.97 0.98
2.07
3.06
f1 (ppm)

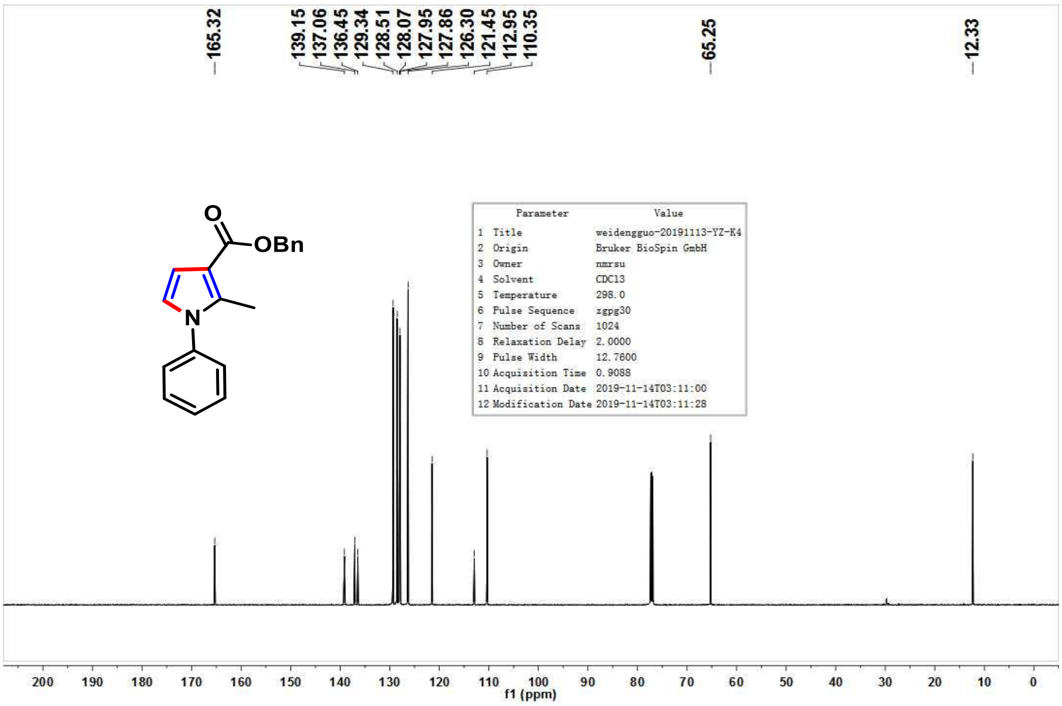

165.32
139.15 137.06 136.45 129.34 128.51 128.07 127.95 127.86 126.30 121.45 112.95 110.35
65.25
12.33
OBn
N
Parameter Value
1 Title weidengguo-20191113-YZ-K4
2 Origin Bruker BioSpin GmbH
3 Owner nmrsu
4 Solvent CDCl3
5 Temperature 298.0
6 Pulse Sequence zgpg30
7 Number of Scans 1024
8 Relaxation Delay 2.0000
9 Pulse Width 12.7600
10 Acquisition Time 0.9088
11 Acquisition Date 2019-11-14T03:11:00
12 Modification Date 2019-11-14T03:11:28
f1 (ppm)

4e

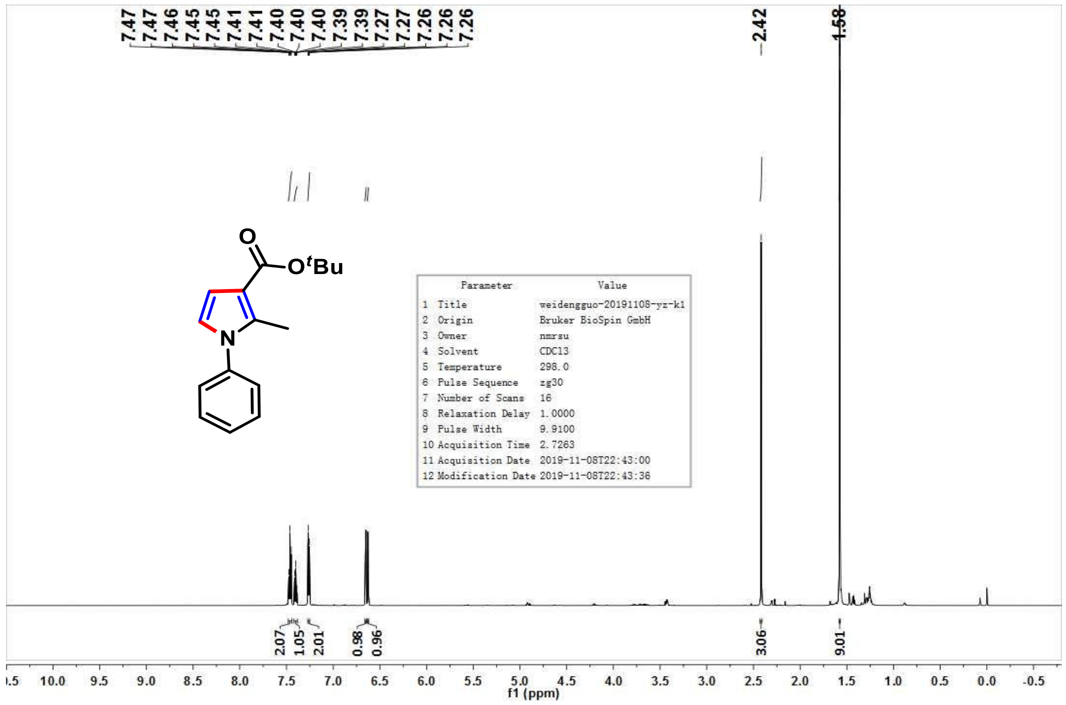
7.47
7.47
7.46
7.45
7.45
7.41
7.41
7.40
7.40
7.40
7.39
7.39
7.27
7.27
7.26
7.26
7.26
2.42
1.58
O
O^{t}Bu
N
Parameter Value
1 Title weidengguo-20191108-yz-k1
2 Origin Bruker BioSpin GmbH
3 Owner nmrsu
4 Solvent CDCl3
5 Temperature 298.0
6 Pulse Sequence zg30
7 Number of Scans 16
8 Relaxation Delay 1.0000
9 Pulse Width 9.9100
10 Acquisition Time 2.7263
11 Acquisition Date 2019-11-08T22:43:00
12 Modification Date 2019-11-08T22:43:36
2.07
1.05
2.01
0.98
0.96
3.06
9.01
f1 (ppm)

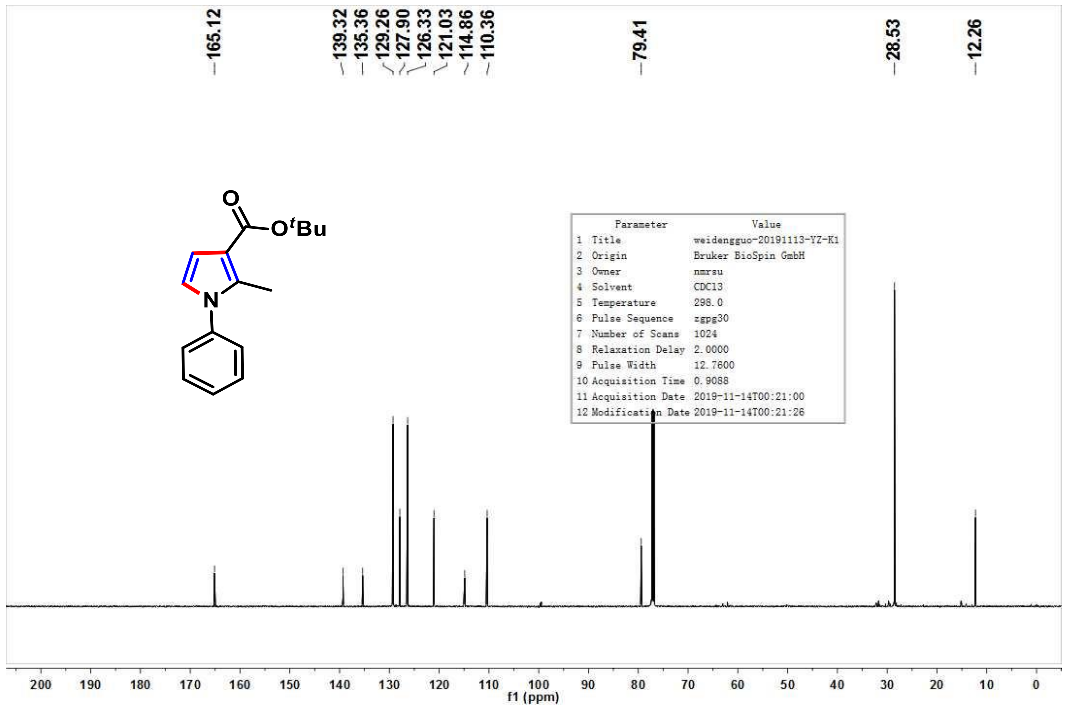
165.12
139.32
135.36
129.26
127.90
126.33
121.03
114.86
110.36
79.41
28.53
12.26
O
O^{t}Bu
N
Parameter Value
1 Title weidengguo-20191113-YZ-K1
2 Origin Bruker BioSpin GmbH
3 Owner nmrsu
4 Solvent CDCl3
5 Temperature 298.0
6 Pulse Sequence zgpg30
7 Number of Scans 1024
8 Relaxation Delay 2.0000
9 Pulse Width 12.7600
10 Acquisition Time 0.9088
11 Acquisition Date 2019-11-14T00:21:00
12 Modification Date 2019-11-14T00:21:26
f1 (ppm)

4f

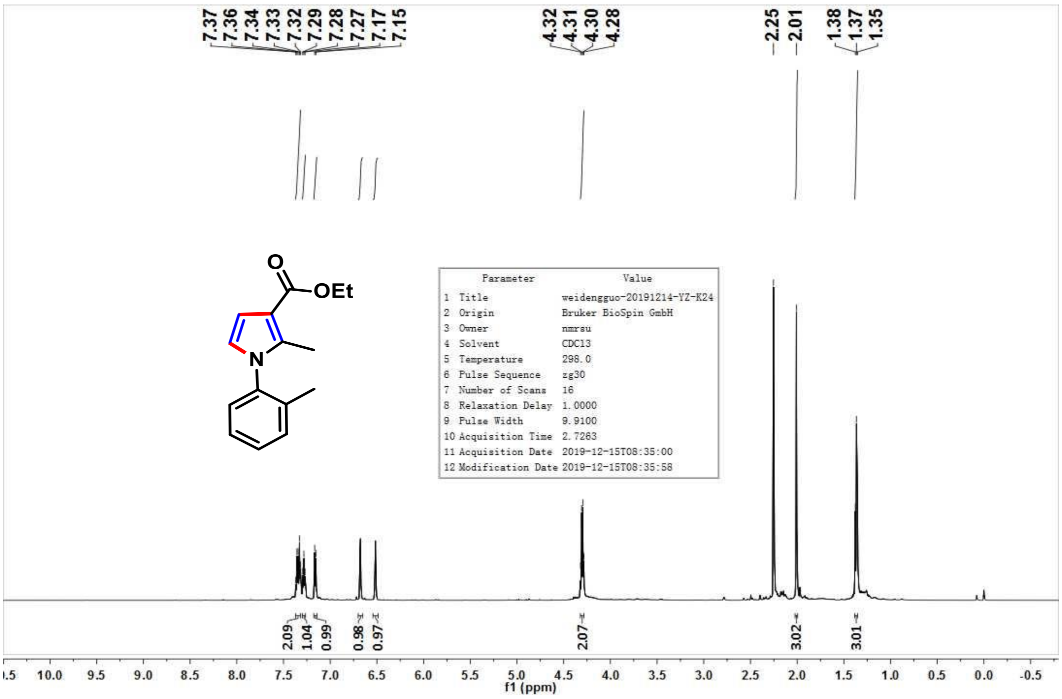
7.37
7.36
7.34
7.33
7.32
7.29
7.28
7.27
7.17
7.15
4.32
4.31
4.30
4.28
2.25
2.01
1.38
1.37
1.35
O
OEt
N
Parameter Value
1 Title weidengguo-20191214-YZ-K24
2 Origin Bruker BioSpin GmbH
3 Owner nmrsu
4 Solvent CDCl3
5 Temperature 298.0
6 Pulse Sequence zg30
7 Number of Scans 16
8 Relaxation Delay 1.0000
9 Pulse Width 9.9100
10 Acquisition Time 2.7263
11 Acquisition Date 2019-12-15T08:35:00
12 Modification Date 2019-12-15T08:35:58
2.09
1.04
0.99
0.98
0.97
2.07
3.02
3.01
f1 (ppm)

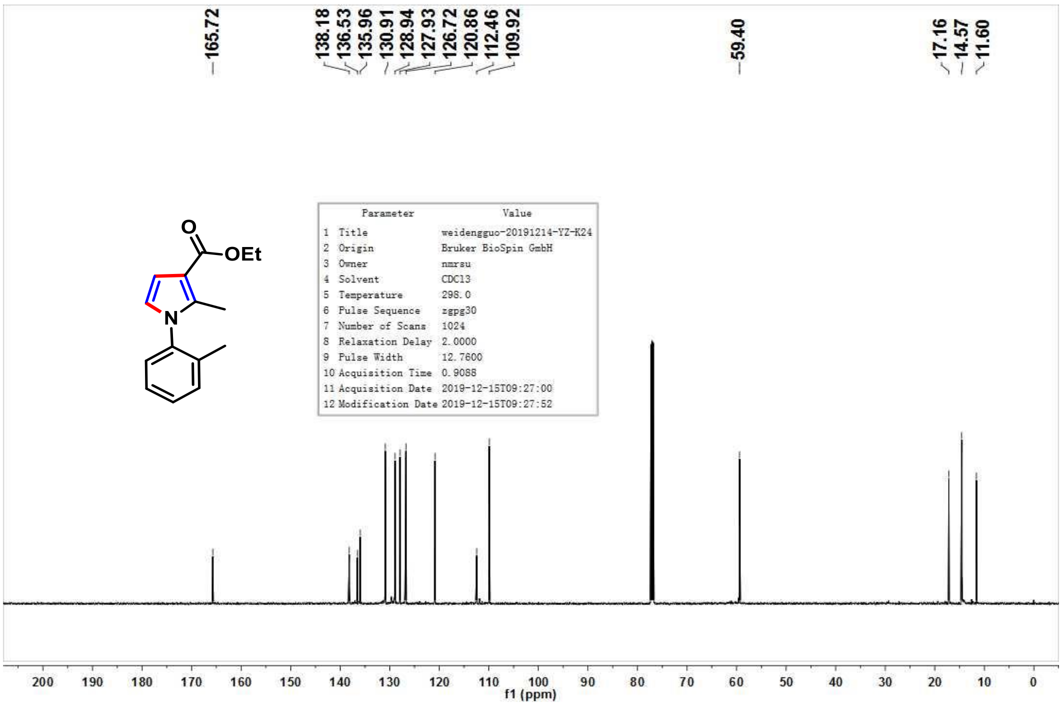
165.72
138.18
136.53
135.96
130.91
128.94
127.93
126.72
120.86
112.46
109.92
59.40
17.16
14.57
11.60
O
OEt
N
Parameter Value
1 Title weidengguo-20191214-YZ-K24
2 Origin Bruker BioSpin GmbH
3 Owner nmrsu
4 Solvent CDCl3
5 Temperature 298.0
6 Pulse Sequence zgpg30
7 Number of Scans 1024
8 Relaxation Delay 2.0000
9 Pulse Width 12.7600
10 Acquisition Time 0.9088
11 Acquisition Date 2019-12-15T09:27:00
12 Modification Date 2019-12-15T09:27:52
f1 (ppm)

4g

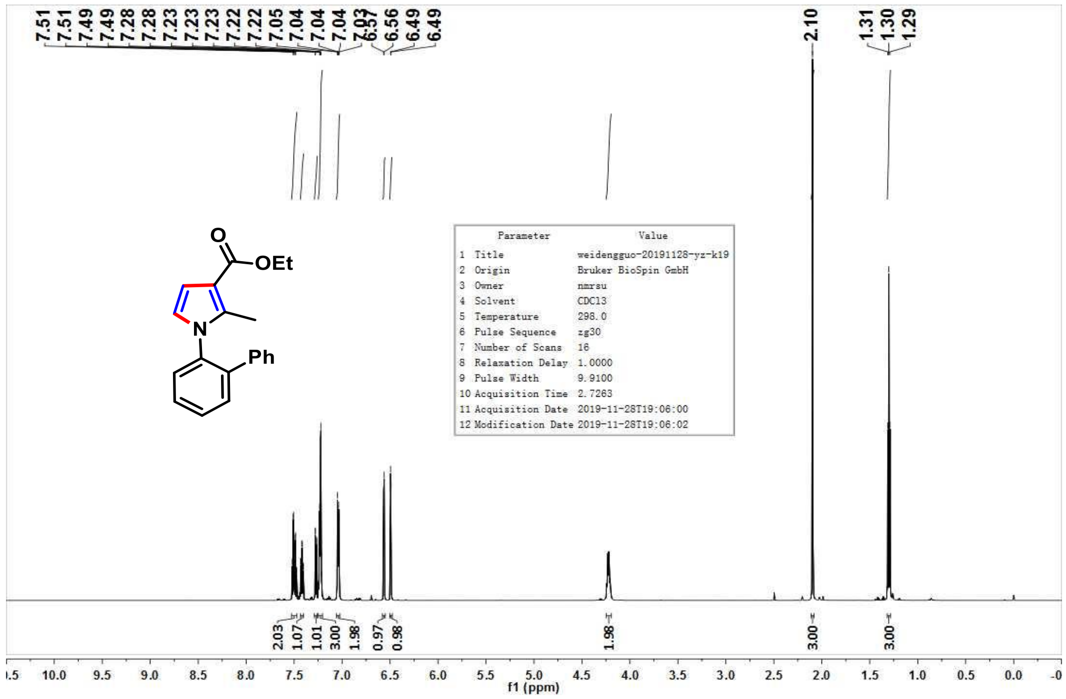

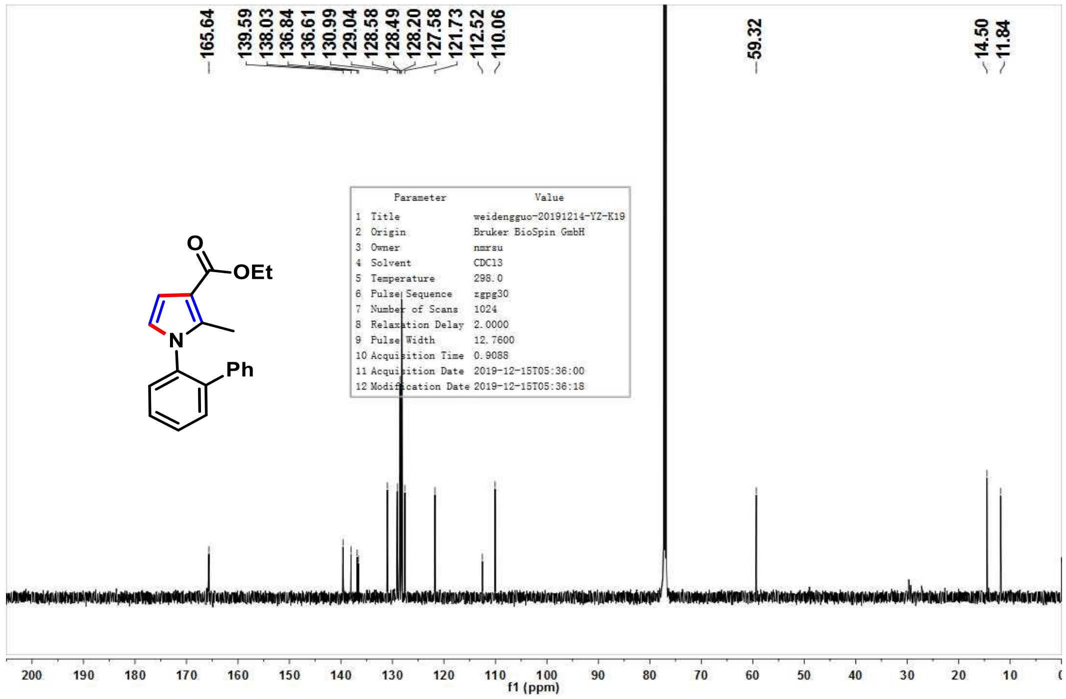

4h

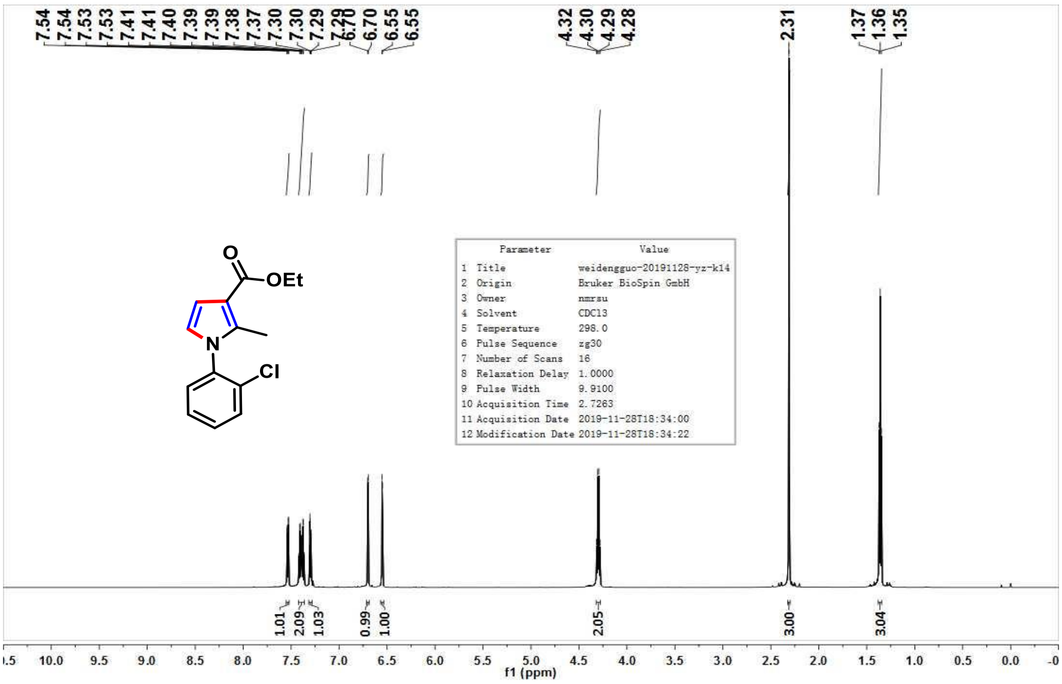

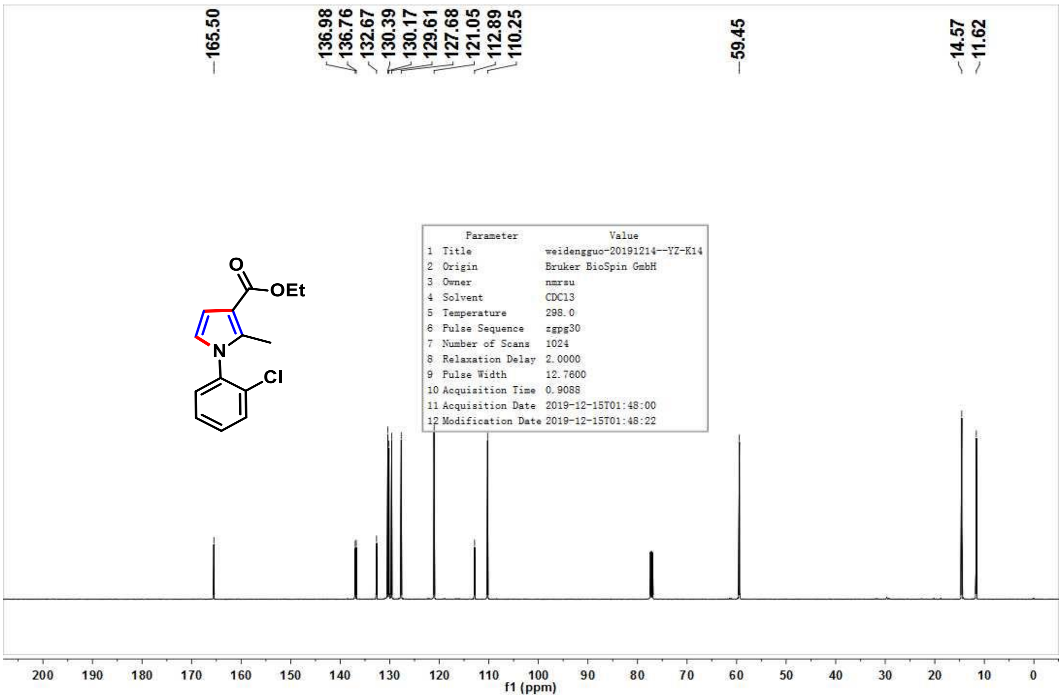

4i

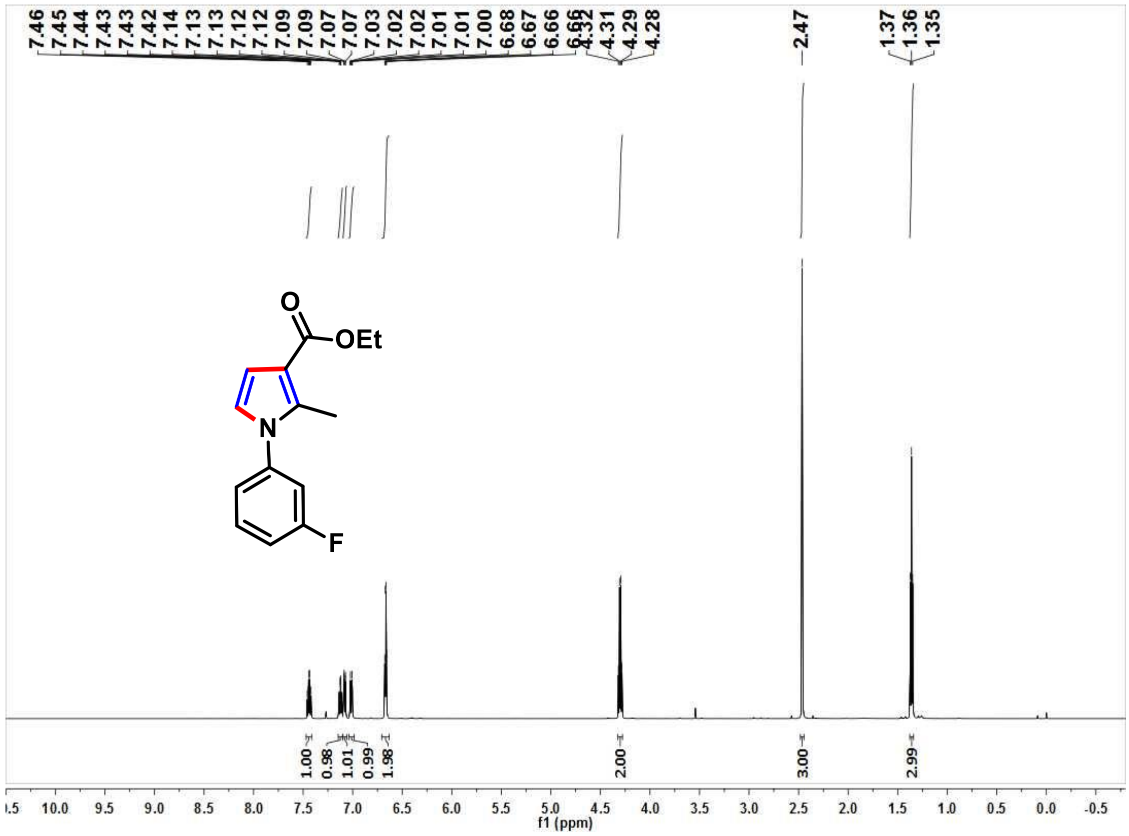

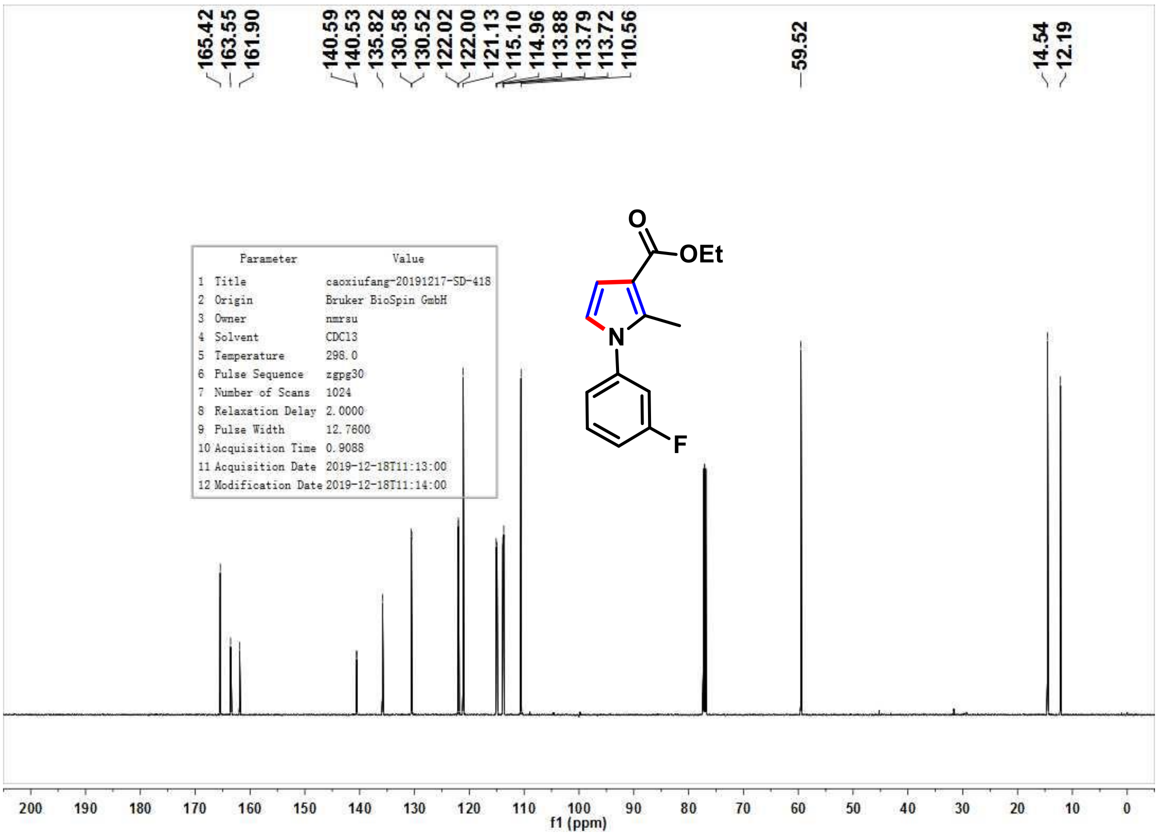

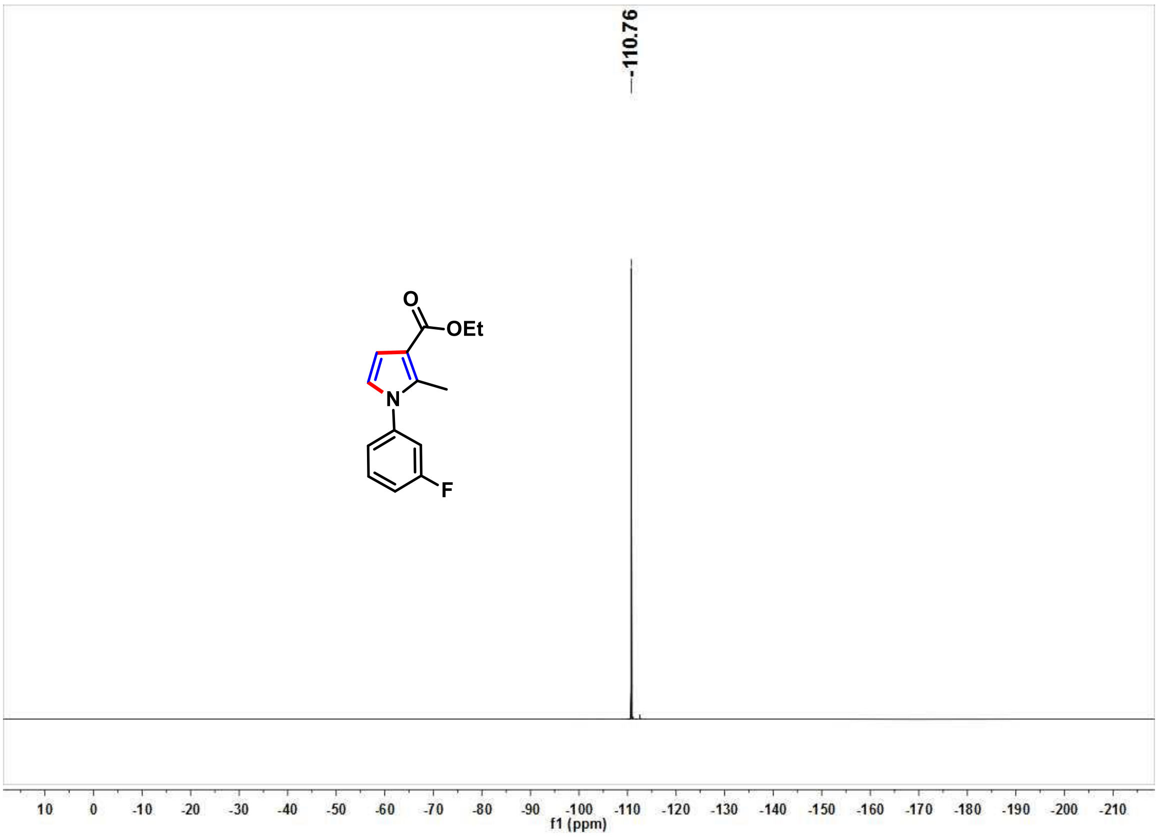

-110.76
OEt
O
N
F
10
0
-10
-20
-30
-40
-50
-60
-70
-80
-90
-100
-110
-120
-130
-140
-150
-160
-170
-180
-190
-200
-210
f1 (ppm)

4j

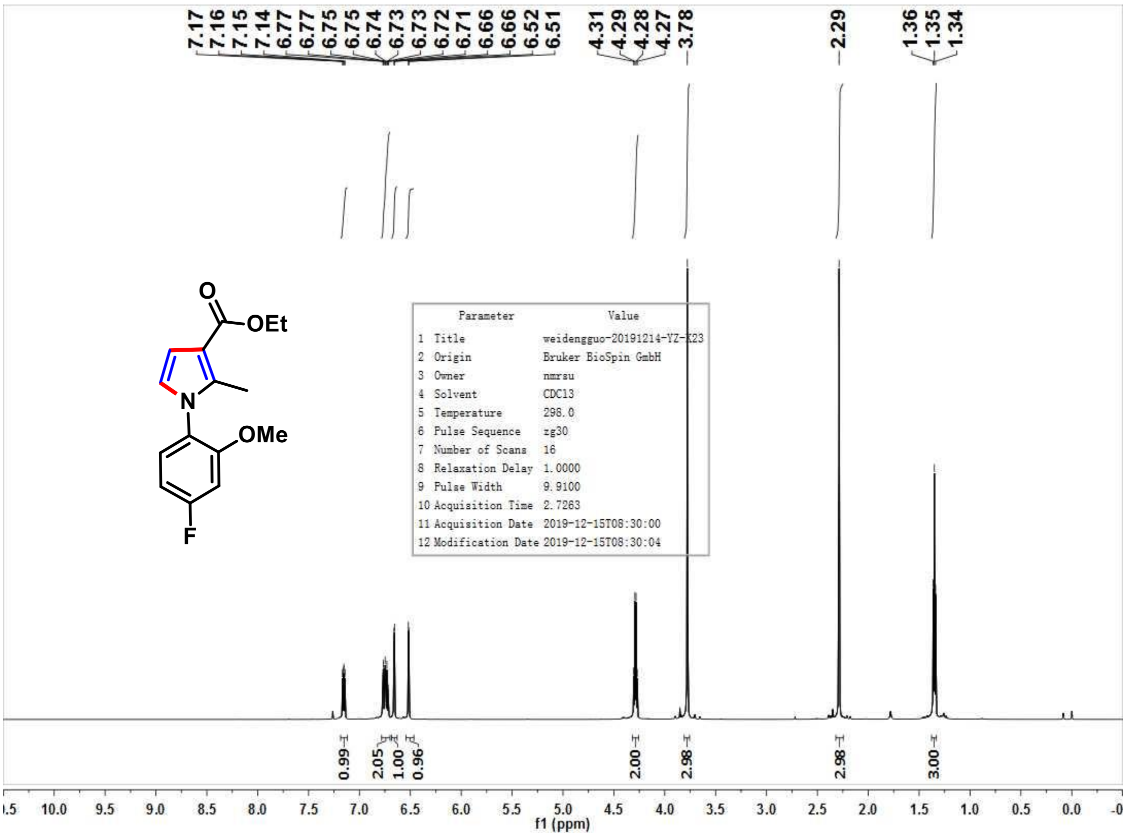
7.17
7.16
7.15
7.14
6.77
6.77
6.75
6.75
6.74
6.73
6.73
6.72
6.71
6.66
6.66
6.52
6.51
4.31
4.29
4.28
4.27
3.78
2.29
1.36
1.35
1.34
O
OEt
N
OMe
F
Parameter
Value
1 Title weidengguo-20191214-YZ-K23
2 Origin Bruker BioSpin GmbH
3 Owner nmrsu
4 Solvent CDCl3
5 Temperature 298.0
6 Pulse Sequence zg30
7 Number of Scans 16
8 Relaxation Delay 1.0000
9 Pulse Width 9.9100
10 Acquisition Time 2.7263
11 Acquisition Date 2019-12-15T08:30:00
12 Modification Date 2019-12-15T08:30:04
0.99
2.05
1.00
0.96
2.00
2.98
2.98
3.00
f1 (ppm)

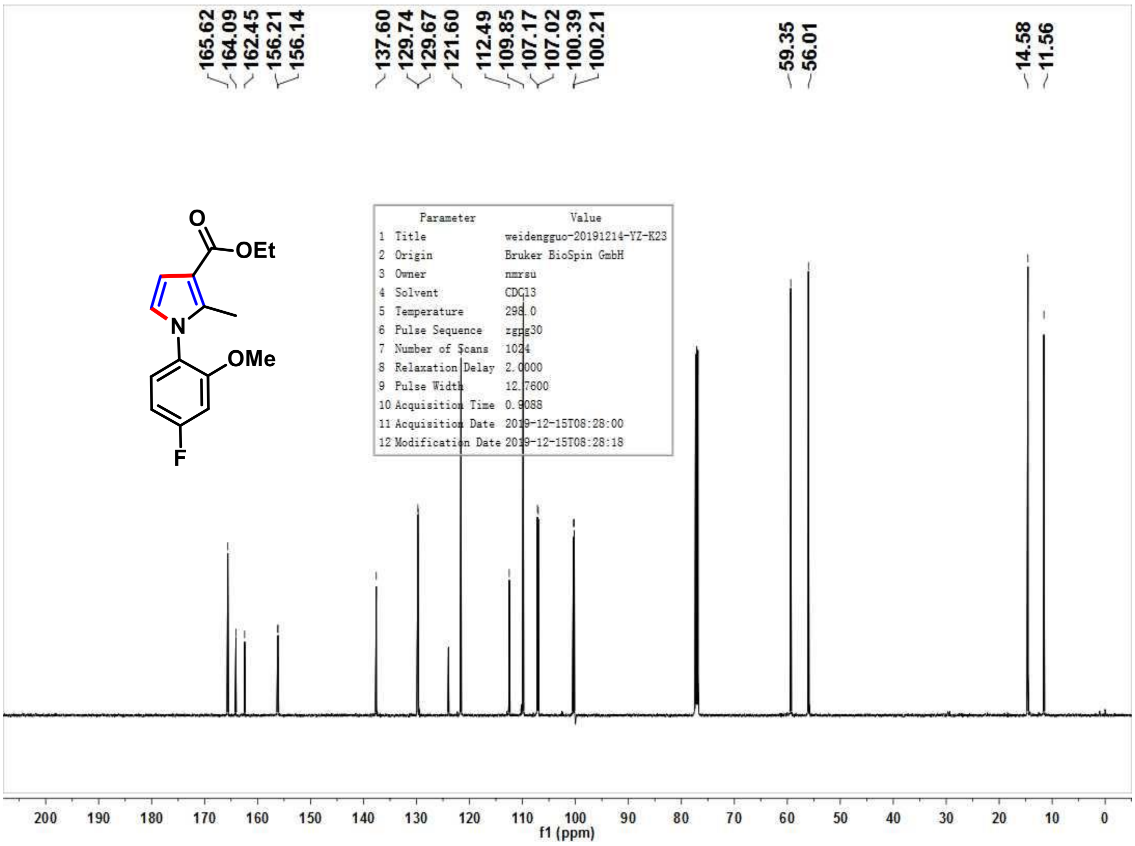
165.62
164.09
162.45
156.21
156.14
137.60
129.74
129.67
121.60
112.49
109.85
107.17
107.02
100.39
100.21
59.35
56.01
14.58
11.56
O
OEt
N
OMe
F
Parameter
Value
1 Title weidengguo-20191214-YZ-K23
2 Origin Bruker BioSpin GmbH
3 Owner nmrsu
4 Solvent CDCl3
5 Temperature 298.0
6 Pulse Sequence zgpg30
7 Number of Scans 1024
8 Relaxation Delay 2.0000
9 Pulse Width 12.7600
10 Acquisition Time 0.9088
11 Acquisition Date 2019-12-15T08:28:00
12 Modification Date 2019-12-15T08:28:18
f1 (ppm)

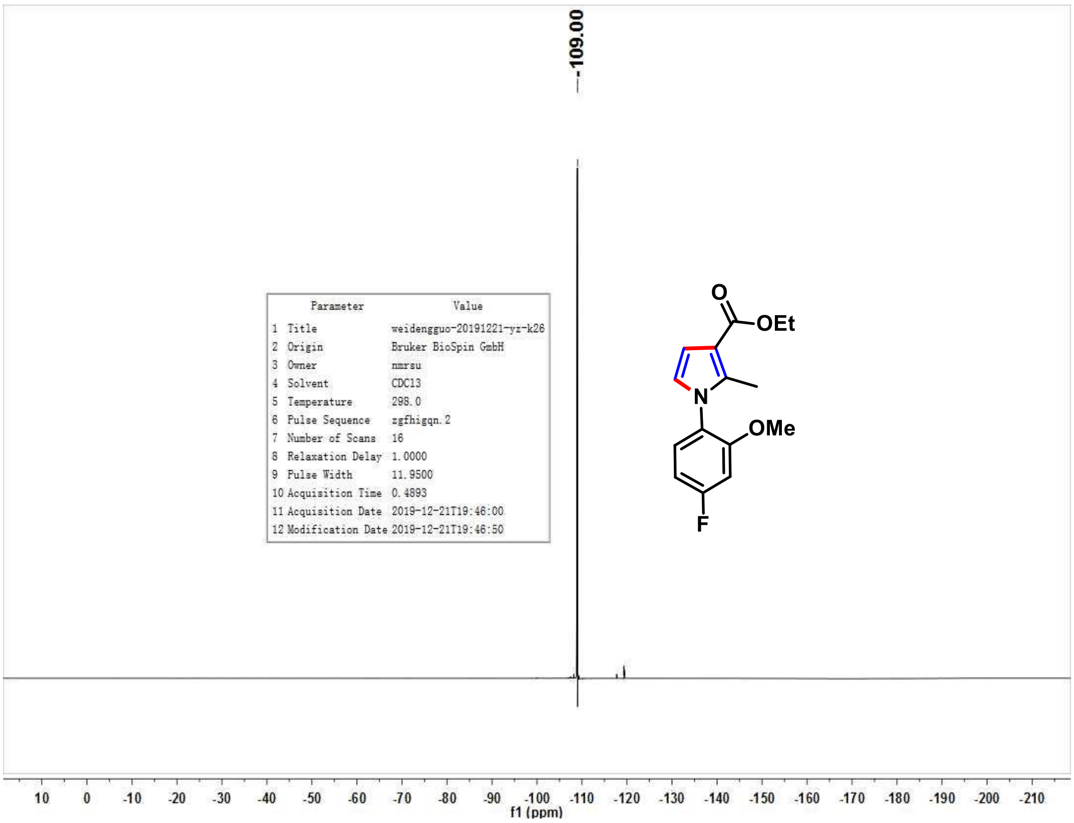
-109.00
Parameter
Value
1 Title
weidengguo-20191221-yz-k26
2 Origin
Bruker BioSpin GmbH
3 Owner
nmrsu
4 Solvent
CDCl3
5 Temperature
298.0
6 Pulse Sequence
zgfhigqn.2
7 Number of Scans
16
8 Relaxation Delay
1.0000
9 Pulse Width
11.9500
10 Acquisition Time
0.4893
11 Acquisition Date
2019-12-21T19:46:00
12 Modification Date
2019-12-21T19:46:50
O
OEt
N
OMe
F
10 0 -10 -20 -30 -40 -50 -60 -70 -80 -90 -100 -110 -120 -130 -140 -150 -160 -170 -180 -190 -200 -210
f1 (ppm)

4k

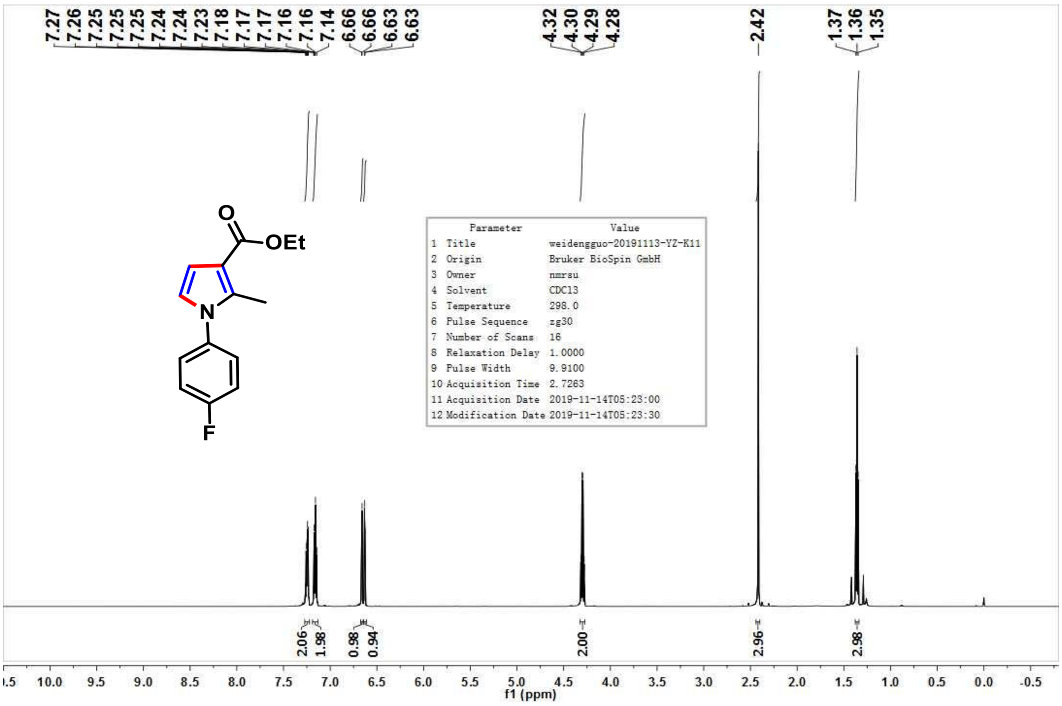
7.27
7.26
7.25
7.25
7.25
7.24
7.24
7.23
7.18
7.17
7.17
7.16
7.16
7.14
6.66
6.66
6.63
6.63
4.32
4.30
4.29
4.28
2.42
1.37
1.36
1.35
O
OEt
N
F
Parameter Value
1 Title weidengguo-20191113-YZ-K11
2 Origin Bruker BioSpin GmbH
3 Owner nmrsu
4 Solvent CDCl3
5 Temperature 298.0
6 Pulse Sequence zg30
7 Number of Scans 16
8 Relaxation Delay 1.0000
9 Pulse Width 9.9100
10 Acquisition Time 2.7263
11 Acquisition Date 2019-11-14T05:23:00
12 Modification Date 2019-11-14T05:23:30
2.06
1.98
0.98
0.94
2.00
2.96
2.98
f1 (ppm)

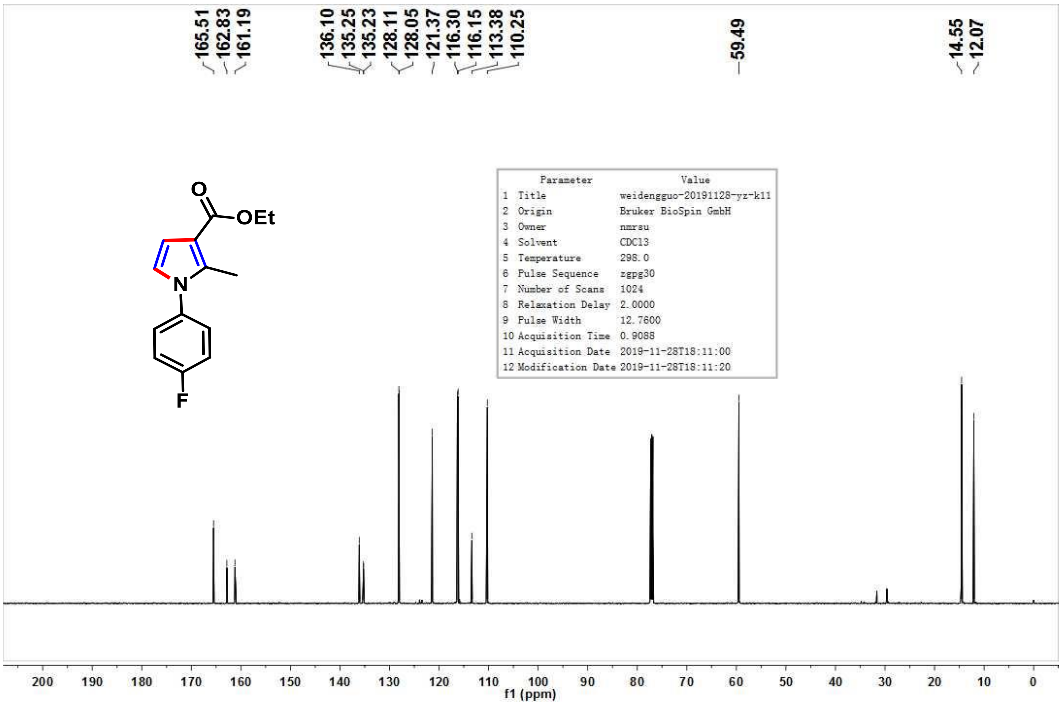
165.51
162.83
161.19
136.10
135.25
135.23
128.11
128.05
121.37
116.30
116.15
113.38
110.25
59.49
14.55
12.07
O
OEt
N
F
Parameter Value
1 Title weidengguo-20191128-yz-k11
2 Origin Bruker BioSpin GmbH
3 Owner nmrsu
4 Solvent CDCl3
5 Temperature 298.0
6 Pulse Sequence zgpg30
7 Number of Scans 1024
8 Relaxation Delay 2.0000
9 Pulse Width 12.7600
10 Acquisition Time 0.9088
11 Acquisition Date 2019-11-28T18:11:00
12 Modification Date 2019-11-28T18:11:20
f1 (ppm)

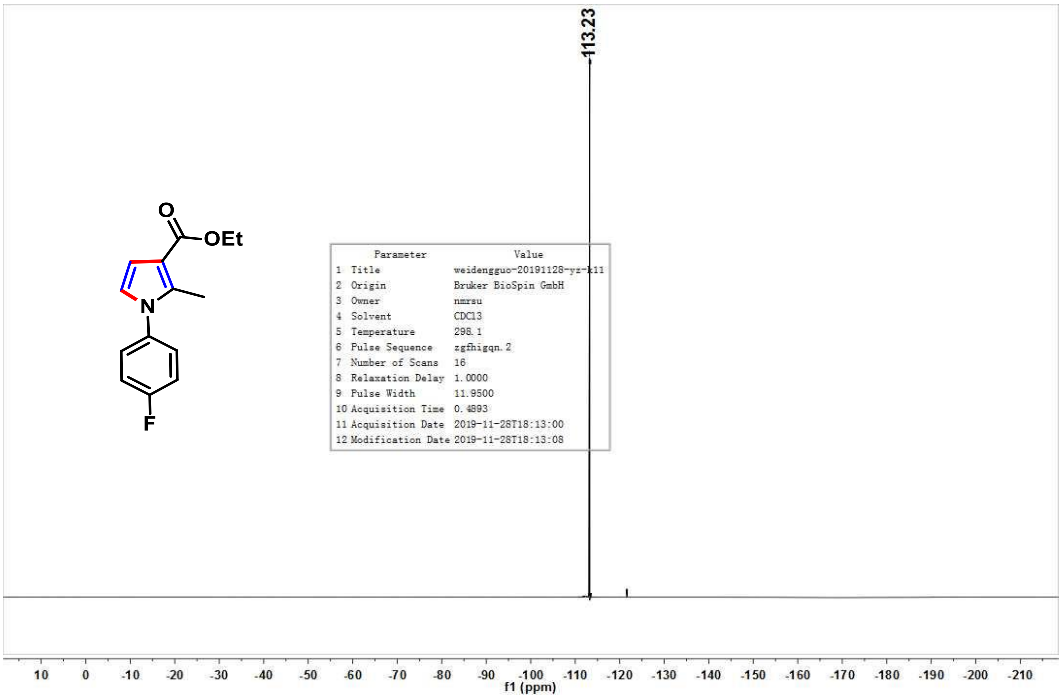

-113.23
O
OEt
N
F
Parameter Value
1 Title weidengguo-20191128-yz-k11
2 Origin Bruker BioSpin GmbH
3 Owner nmrsu
4 Solvent CDCl3
5 Temperature 298.1
6 Pulse Sequence zgfhigqn.2
7 Number of Scans 16
8 Relaxation Delay 1.0000
9 Pulse Width 11.9500
10 Acquisition Time 0.4893
11 Acquisition Date 2019-11-28T18:13:00
12 Modification Date 2019-11-28T18:13:08
10 0 -10 -20 -30 -40 -50 -60 -70 -80 -90 -100 -110 -120 -130 -140 -150 -160 -170 -180 -190 -200 -210
f1 (ppm)

4l

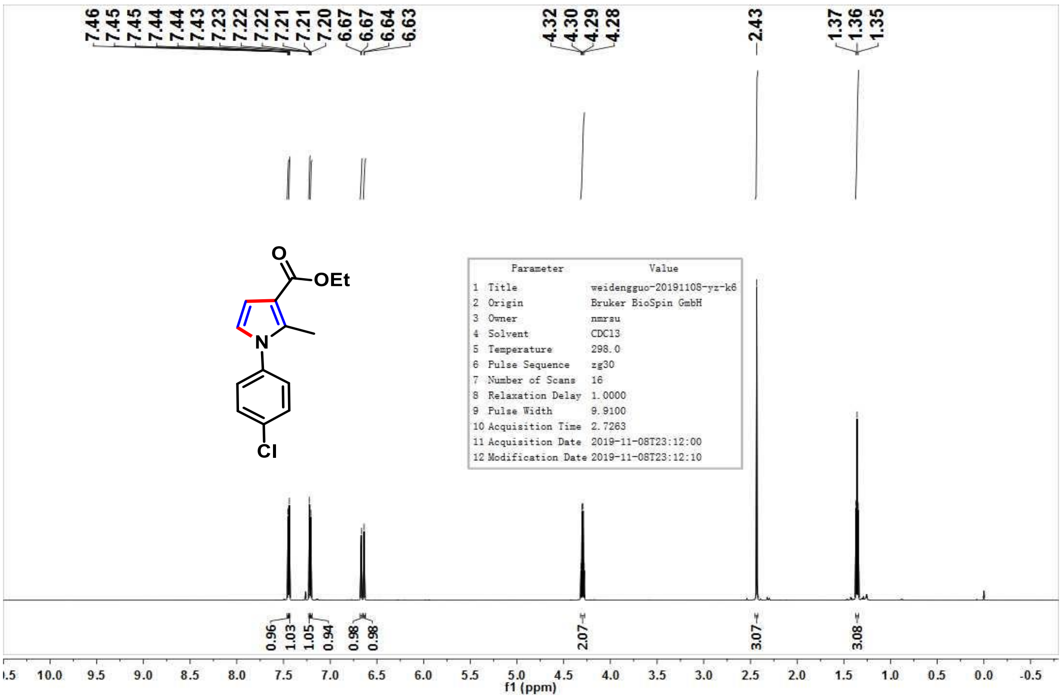
7.46
7.45
7.45
7.44
7.44
7.43
7.23
7.22
7.22
7.21
7.21
7.20
6.67
6.67
6.64
6.63
4.32
4.30
4.29
4.28
2.43
1.37
1.36
1.35
O
OEt
N
Cl
Parameter Value
1 Title weidengguo-20191108-yz-k6
2 Origin Bruker BioSpin GmbH
3 Owner nmrsu
4 Solvent CDCl3
5 Temperature 298.0
6 Pulse Sequence zg30
7 Number of Scans 16
8 Relaxation Delay 1.0000
9 Pulse Width 9.9100
10 Acquisition Time 2.7263
11 Acquisition Date 2019-11-08T23:12:00
12 Modification Date 2019-11-08T23:12:10
0.96
1.03
1.05
0.94
0.98
0.98
2.07
3.07
3.08
f1 (ppm)

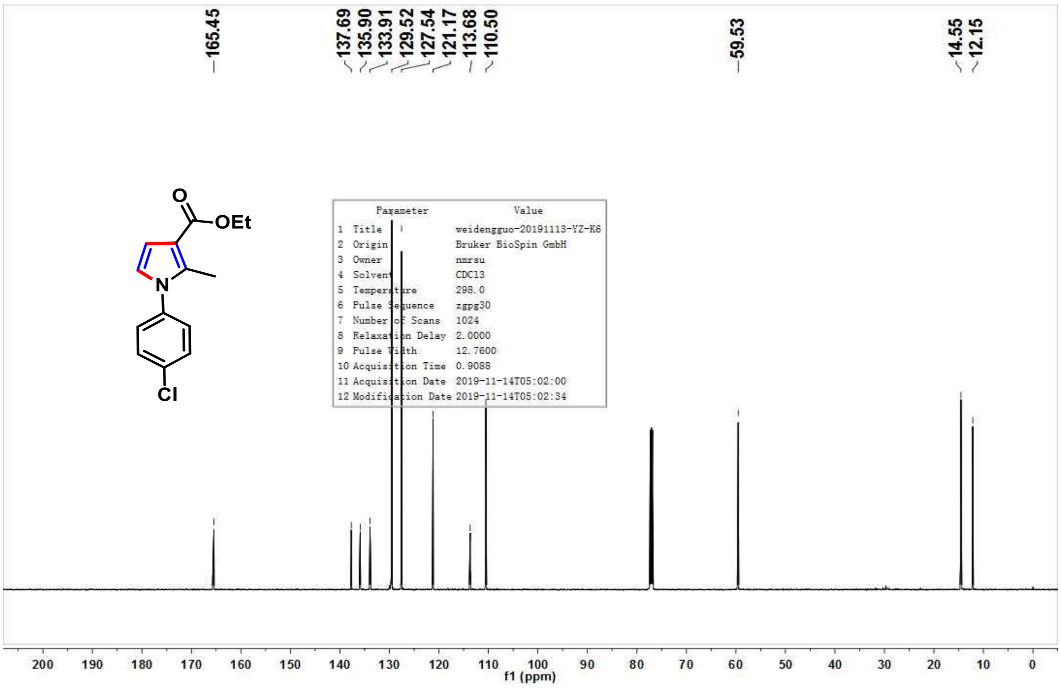
165.45
137.69
135.90
133.91
129.52
127.54
121.17
113.68
110.50
59.53
14.55
12.15
O
OEt
N
Cl
Parameter Value
1 Title weidengguo-20191113-YZ-K6
2 Origin Bruker BioSpin GmbH
3 Owner nmrsu
4 Solvent CDCl3
5 Temperature 298.0
6 Pulse Sequence zgpg30
7 Number of Scans 1024
8 Relaxation Delay 2.0000
9 Pulse Width 12.7600
10 Acquisition Time 0.9088
11 Acquisition Date 2019-11-14T05:02:00
12 Modification Date 2019-11-14T05:02:34
f1 (ppm)

4m

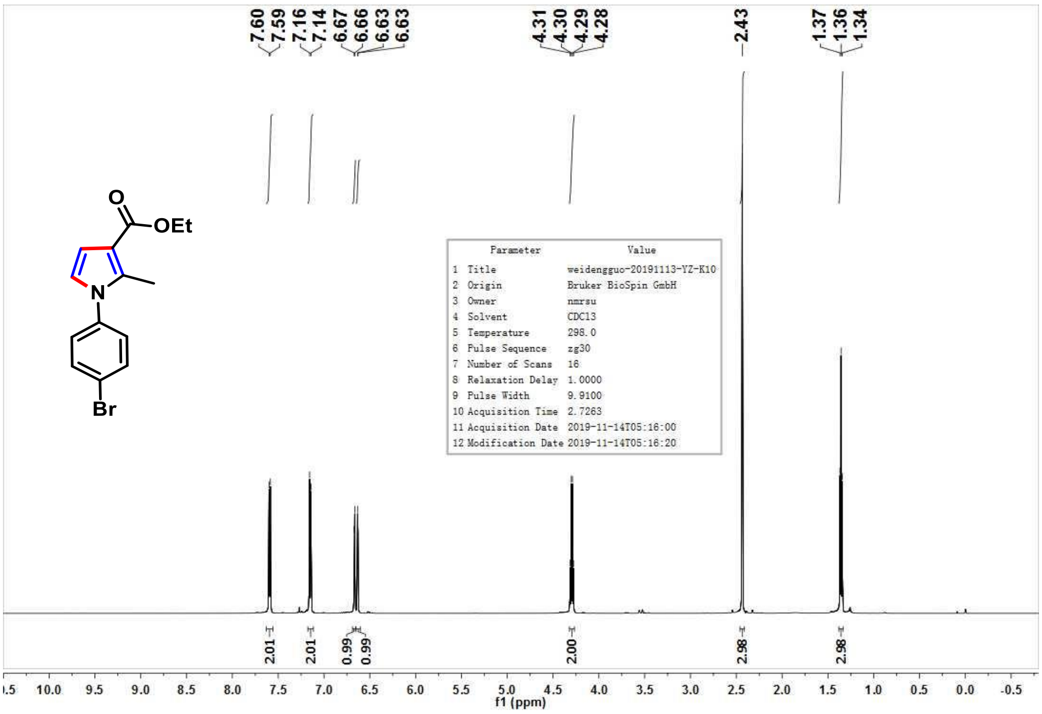

7.60
7.59
7.16
7.14
6.67
6.66
6.63
6.63
4.31
4.30
4.29
4.28
2.43
1.37
1.36
1.34
O
OEt
N
Br
Parameter Value
1 Title weidengguo-20191113-YZ-K10
2 Origin Bruker BioSpin GmbH
3 Owner nmrsu
4 Solvent CDCl3
5 Temperature 298.0
6 Pulse Sequence zg30
7 Number of Scans 16
8 Relaxation Delay 1.0000
9 Pulse Width 9.9100
10 Acquisition Time 2.7263
11 Acquisition Date 2019-11-14T05:16:00
12 Modification Date 2019-11-14T05:16:20
2.01
2.01
0.99
0.99
2.00
2.98
2.98
f1 (ppm)

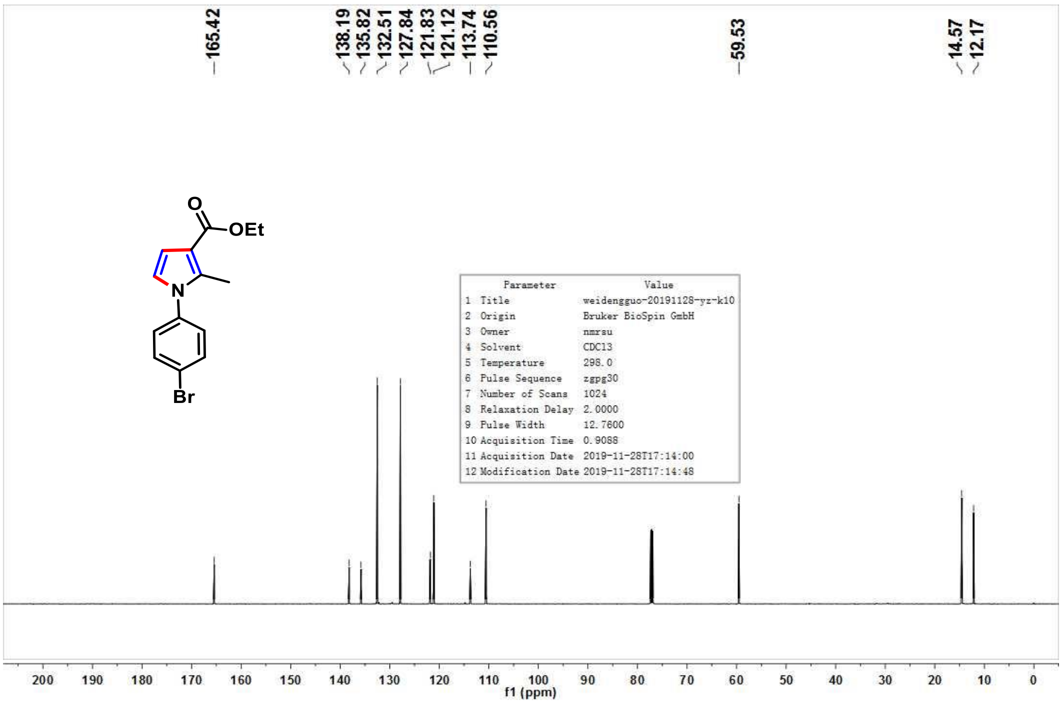

165.42
138.19
135.82
132.51
127.84
121.83
121.12
113.74
110.56
59.53
14.57
12.17
O
OEt
N
Br
Parameter Value
1 Title weidengguo-20191128-yz-k10
2 Origin Bruker BioSpin GmbH
3 Owner nmrsu
4 Solvent CDCl3
5 Temperature 298.0
6 Pulse Sequence zgpg30
7 Number of Scans 1024
8 Relaxation Delay 2.0000
9 Pulse Width 12.7600
10 Acquisition Time 0.9088
11 Acquisition Date 2019-11-28T17:14:00
12 Modification Date 2019-11-28T17:14:48
f1 (ppm)

4n

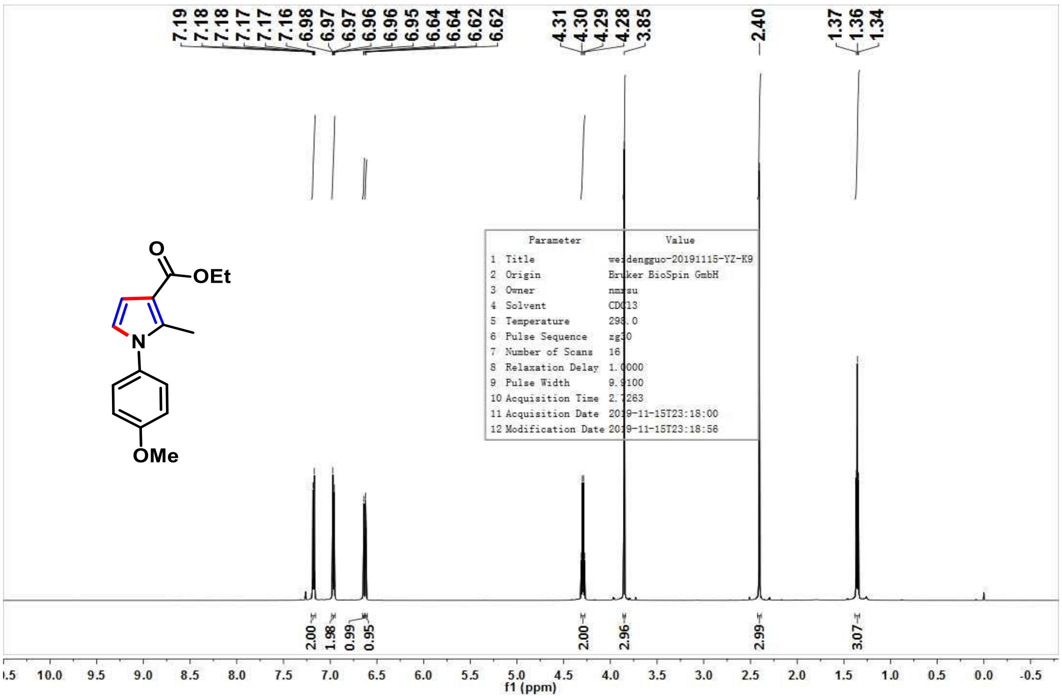

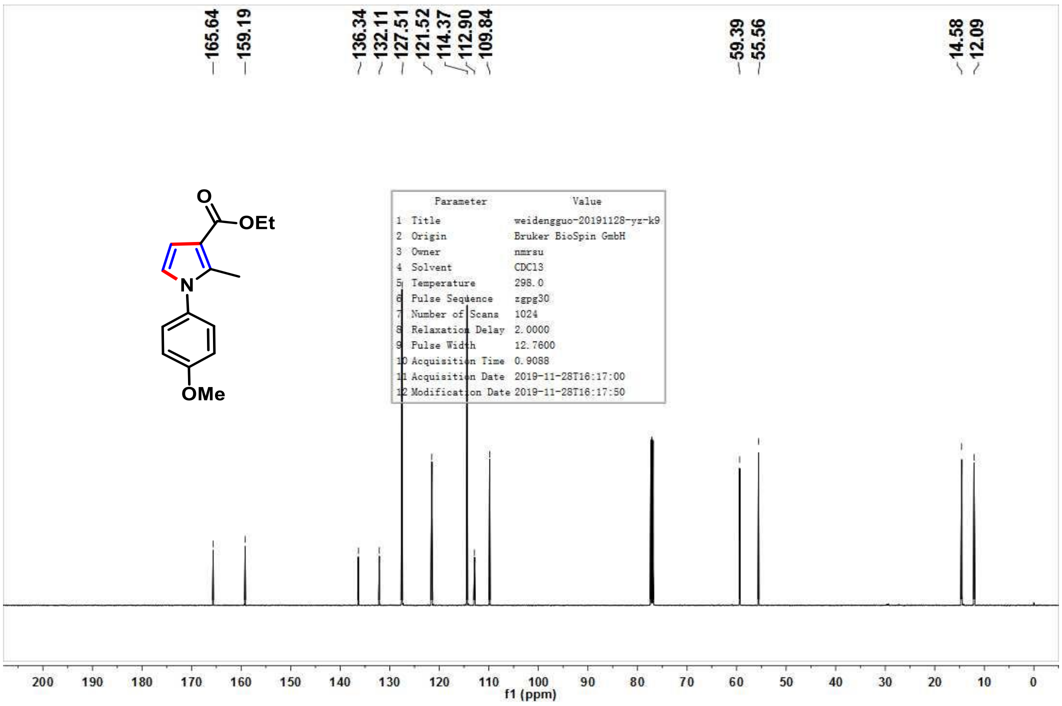

4o

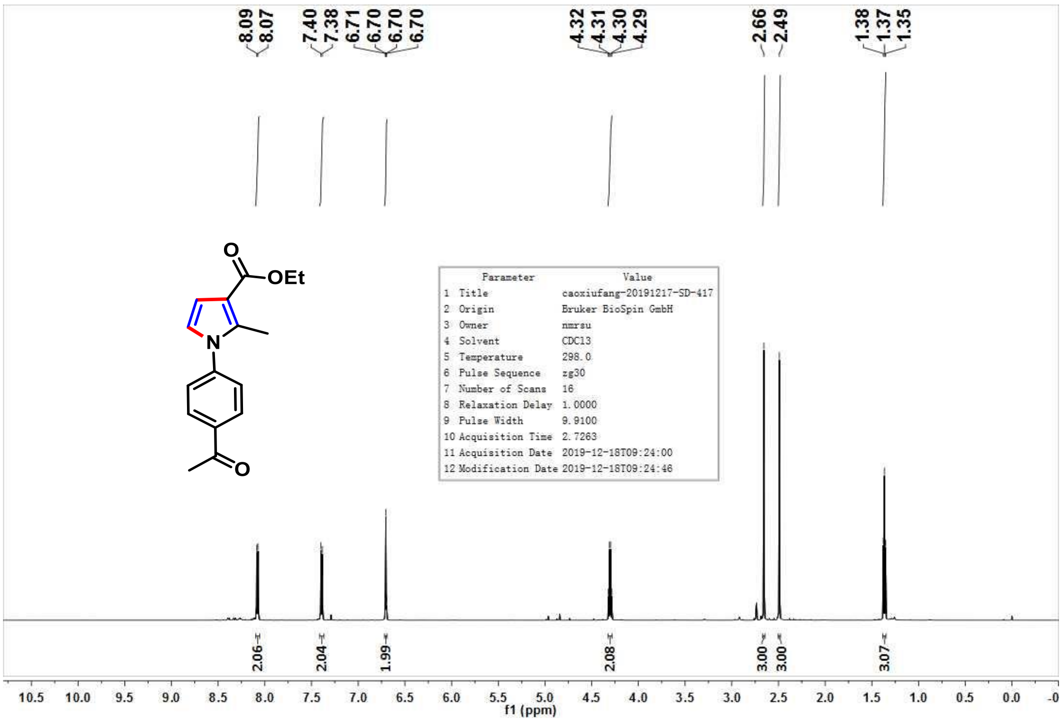

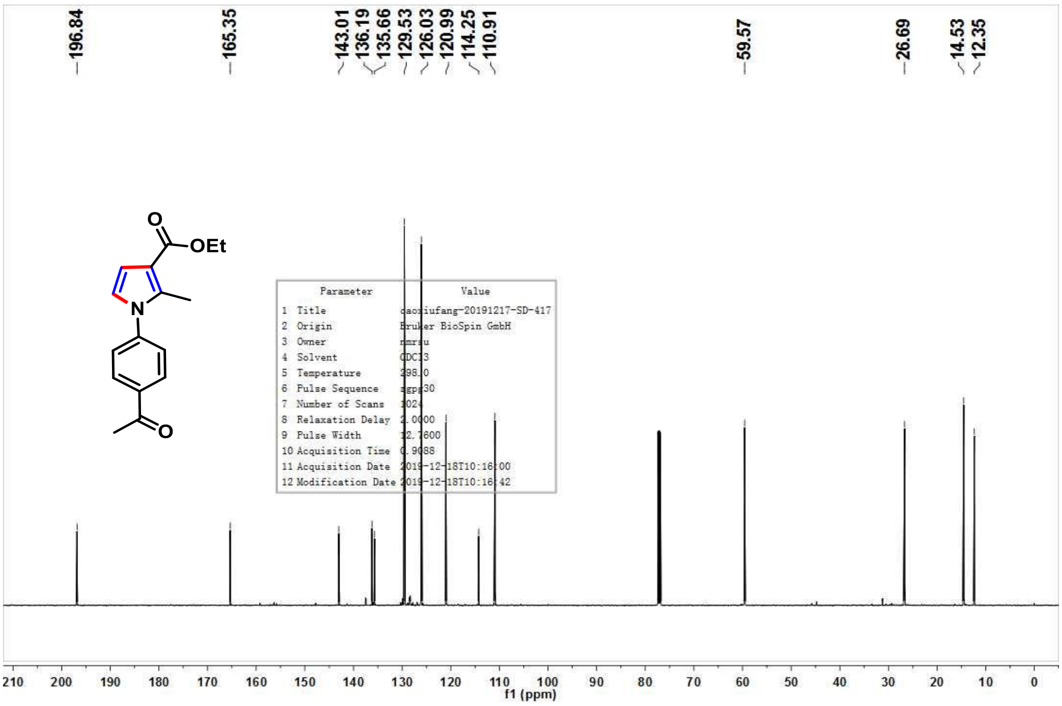

4p

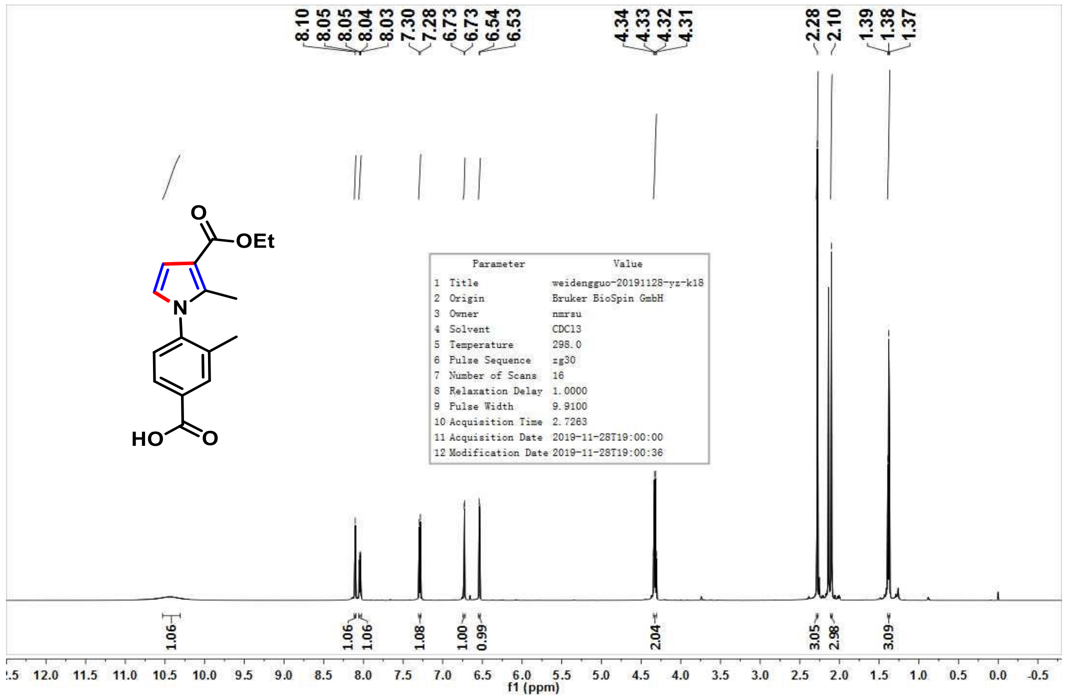
8.10
8.05
8.05
8.04
8.03
7.30
7.28
6.73
6.73
6.54
6.53
4.34
4.33
4.32
4.31
2.28
2.10
1.39
1.38
1.37
OEt
N
HO
Parameter Value
1 Title weidengguo-20191128-yz-k18
2 Origin Bruker BioSpin GmbH
3 Owner nmrsu
4 Solvent CDCl3
5 Temperature 298.0
6 Pulse Sequence zg30
7 Number of Scans 16
8 Relaxation Delay 1.0000
9 Pulse Width 9.9100
10 Acquisition Time 2.7263
11 Acquisition Date 2019-11-28T19:00:00
12 Modification Date 2019-11-28T19:00:36
1.06
1.06
1.06
1.08
1.00
0.99
2.04
3.05
2.98
3.09
f1 (ppm)

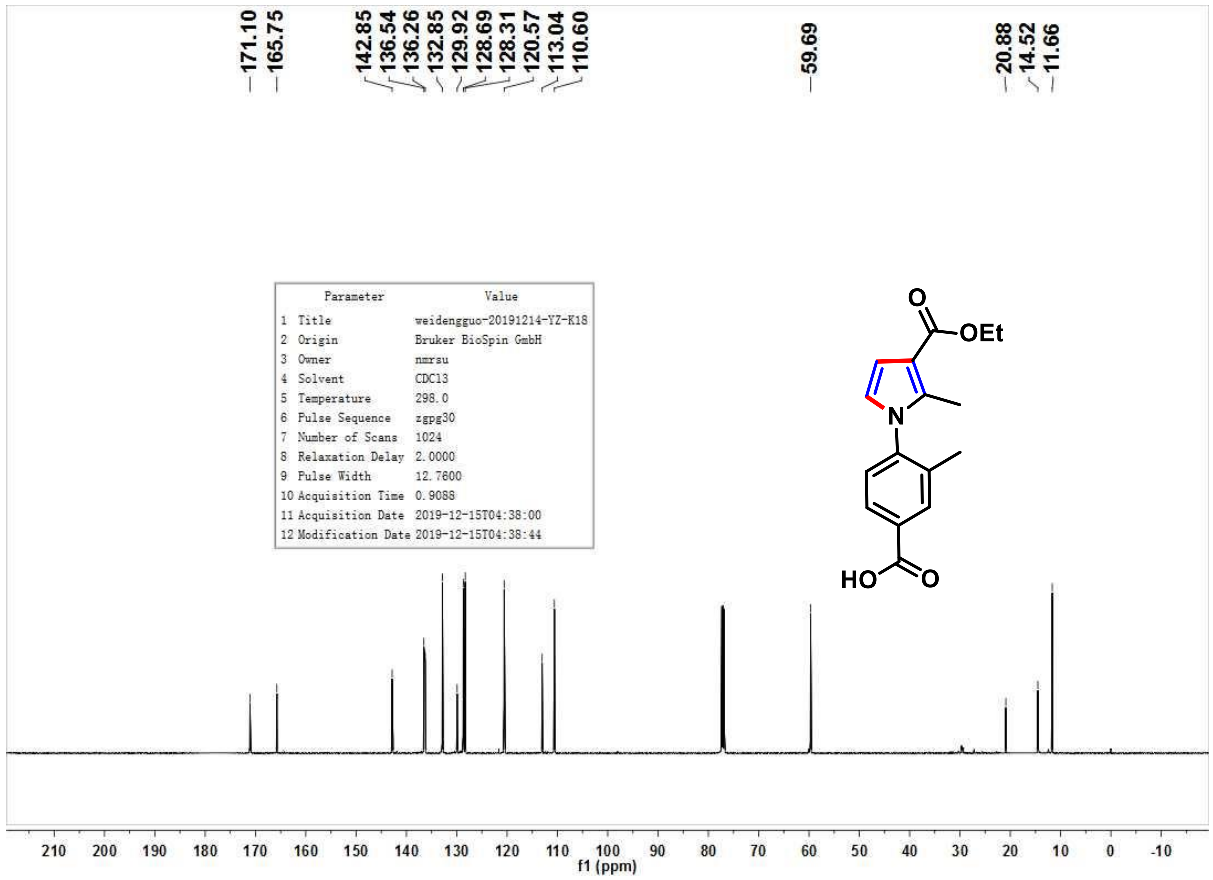
171.10
165.75
142.85
136.54
136.26
132.85
129.92
128.69
128.31
120.57
113.04
110.60
59.69
20.88
14.52
11.66
Parameter Value
1 Title weidengguo-20191214-YZ-K18
2 Origin Bruker BioSpin GmbH
3 Owner nmrsu
4 Solvent CDCl3
5 Temperature 298.0
6 Pulse Sequence zgpg30
7 Number of Scans 1024
8 Relaxation Delay 2.0000
9 Pulse Width 12.7600
10 Acquisition Time 0.9088
11 Acquisition Date 2019-12-15T04:38:00
12 Modification Date 2019-12-15T04:38:44
OEt
N
HO
f1 (ppm)

4q

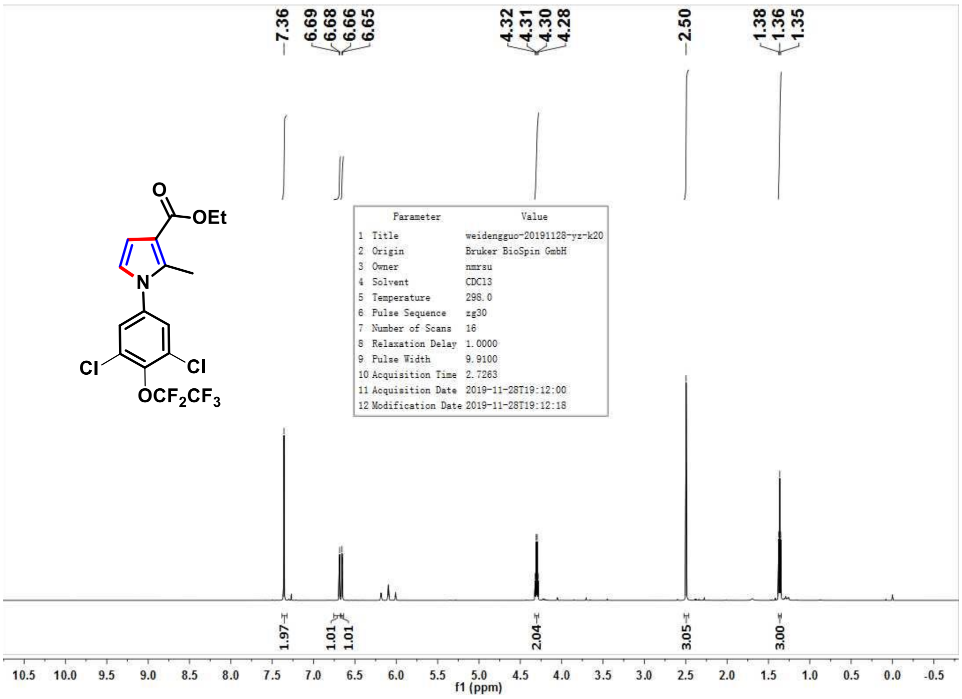

7.36
6.69
6.68
6.66
6.65
4.32
4.31
4.30
4.28
2.50
1.38
1.36
1.35
OEt
O
N
Cl
Cl
OCF2CF3
Parameter Value
1 Title weidengguo-20191128-yz-k20
2 Origin Bruker BioSpin GmbH
3 Owner nmrsu
4 Solvent CDCl3
5 Temperature 298.0
6 Pulse Sequence zg30
7 Number of Scans 16
8 Relaxation Delay 1.0000
9 Pulse Width 9.9100
10 Acquisition Time 2.7263
11 Acquisition Date 2019-11-28T19:12:00
12 Modification Date 2019-11-28T19:12:18
1.97
1.01
1.01
2.04
3.05
3.00
f1 (ppm)

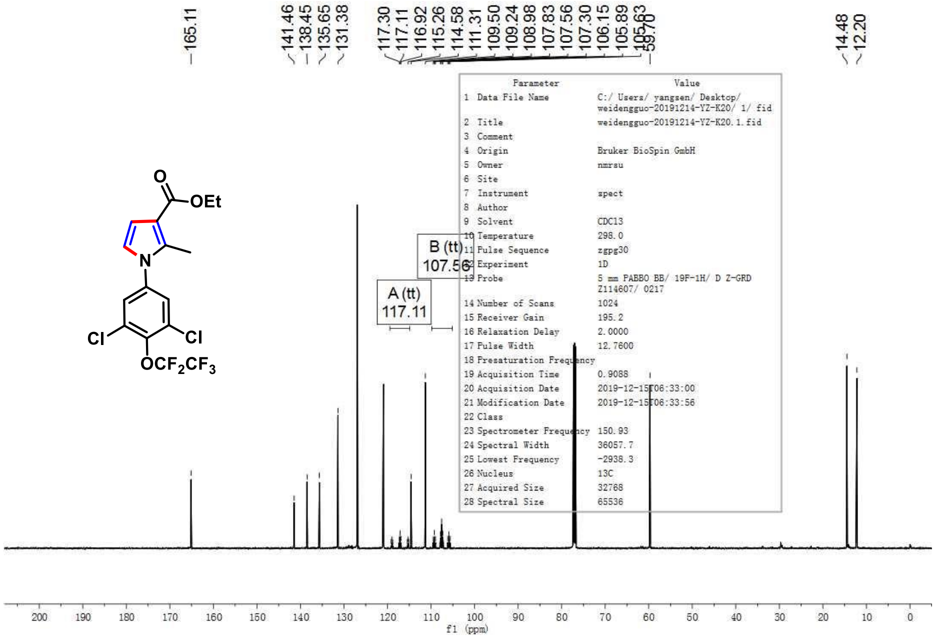

165.11
141.46
138.45
135.65
131.38
117.30
117.11
116.92
115.26
114.58
111.31
109.50
109.24
108.98
107.83
107.56
107.30
106.15
105.89
105.63
59.70
14.48
12.20
B (tt)
107.56
A (tt)
117.11
Parameter Value
1 Data File Name C:/ Users/ yangsen/ Desktop/ weidengguo-20191214-YZ-K20/ 1/ fid
2 Title weidengguo-20191214-YZ-K20.1.fid
3 Comment
4 Origin Bruker BioSpin GmbH
5 Owner nmrsu
6 Site
7 Instrument spect
8 Author
9 Solvent CDCl3
10 Temperature 298.0
11 Pulse Sequence zgpg30
12 Experiment 1D
13 Probe 5 mm PABBO BB/ 19F-1H/ D Z-GRD Z114607/ 0217
14 Number of Scans 1024
15 Receiver Gain 195.2
16 Relaxation Delay 2.0000
17 Pulse Width 12.7600
18 Presaturation Frequency
19 Acquisition Time 0.9088
20 Acquisition Date 2019-12-15T06:33:00
21 Modification Date 2019-12-15T06:33:56
22 Class
23 Spectrometer Frequency 150.93
24 Spectral Width 36057.7
25 Lowest Frequency -2938.3
26 Nucleus 13C
27 Acquired Size 32768
28 Spectral Size 65536
f1 (ppm)

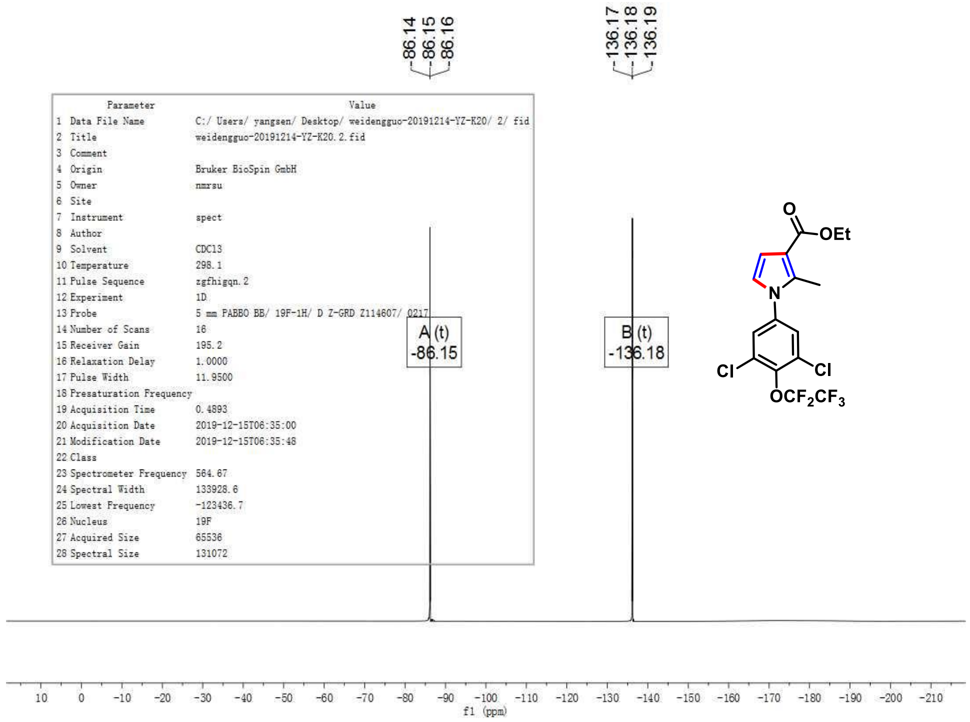

-86.14
-86.15
-86.16
-136.17
-136.18
-136.19
Parameter
Value
1 Data File Name
C:/ Users/ yangsen/ Desktop/ weidengguo-20191214-YZ-K20/ 2/ fid
2 Title
weidengguo-20191214-YZ-K20.2.fid
3 Comment
4 Origin
Bruker BioSpin GmbH
5 Owner
nmrsu
6 Site
7 Instrument
spect
8 Author
9 Solvent
CDCl3
10 Temperature
298.1
11 Pulse Sequence
zgfhigqn.2
12 Experiment
1D
13 Probe
5 mm PABBO BB/ 19F-1H/ D Z-GRD Z114607/ 0217
14 Number of Scans
16
15 Receiver Gain
195.2
16 Relaxation Delay
1.0000
17 Pulse Width
11.9500
18 Presaturation Frequency
19 Acquisition Time
0.4893
20 Acquisition Date
2019-12-15T06:35:00
21 Modification Date
2019-12-15T06:35:48
22 Class
23 Spectrometer Frequency
564.67
24 Spectral Width
133928.6
25 Lowest Frequency
-123436.7
26 Nucleus
19F
27 Acquired Size
65536
28 Spectral Size
131072
A (t)
-86.15
B (t)
-136.18
O
OEt
N
Cl
Cl
OCF2CF3
10 0 -10 -20 -30 -40 -50 -60 -70 -80 -90 -100 -110 -120 -130 -140 -150 -160 -170 -180 -190 -200 -210
f1 (ppm)

4r

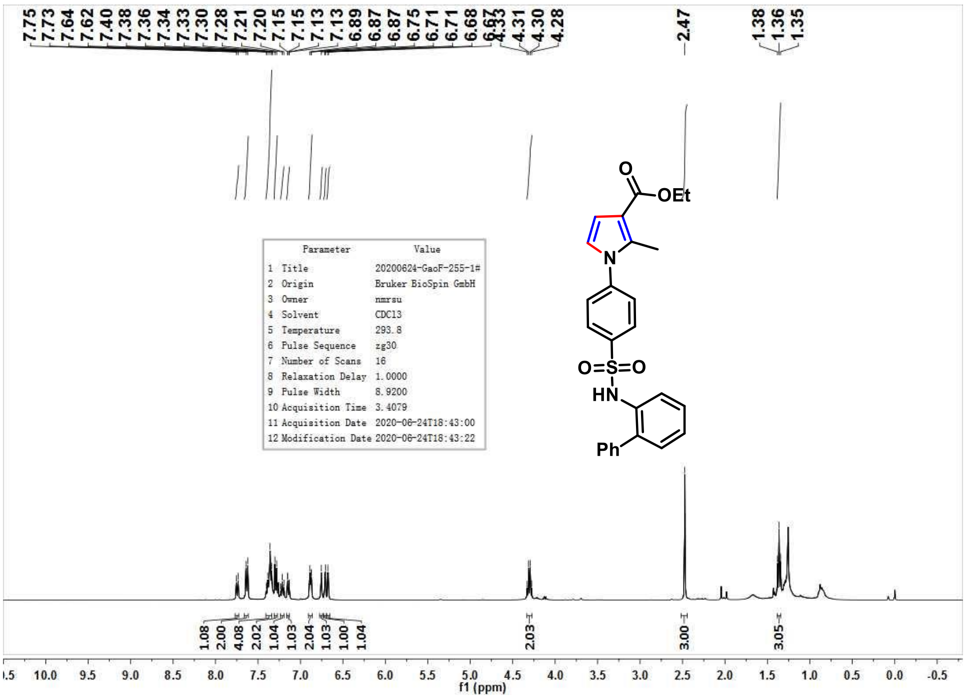

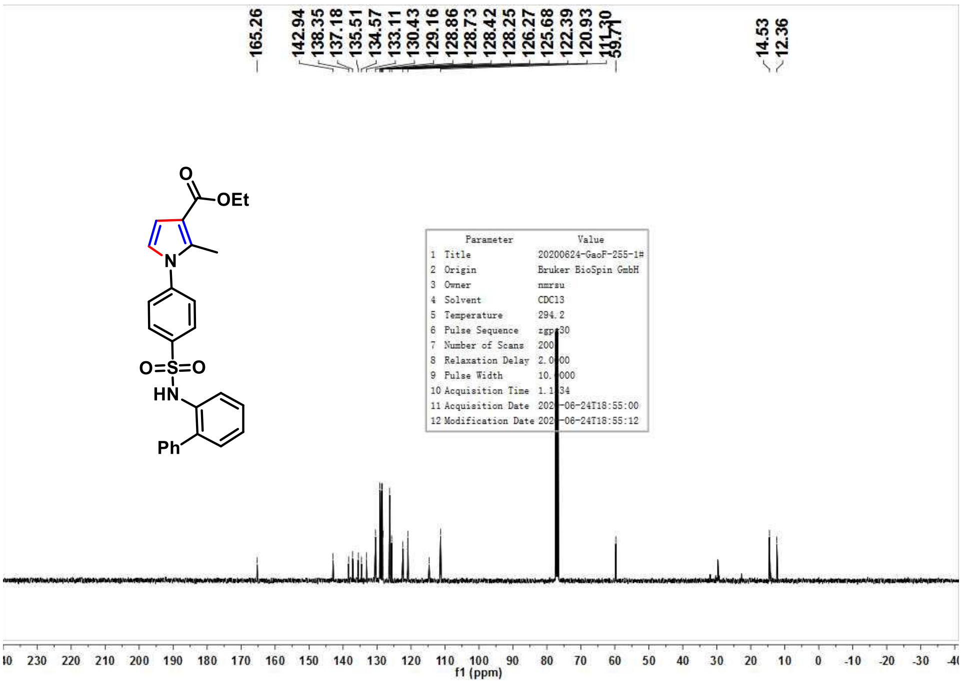

4s

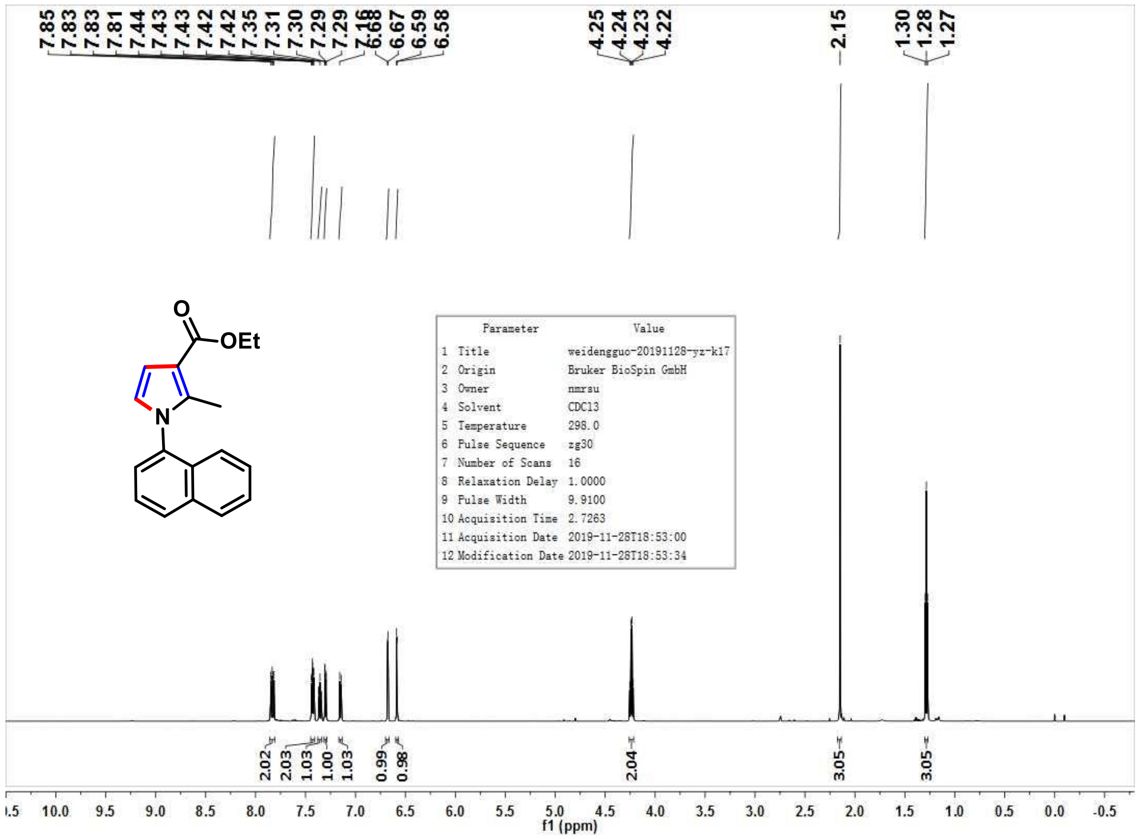
7.85 7.83 7.83 7.81 7.44 7.43 7.43 7.42 7.42 7.35 7.31 7.30 7.29 7.29 7.16 6.68 6.67 6.59 6.58
4.25 4.24 4.23 4.22
2.15
1.30 1.28 1.27
O
OEt
N
Parameter Value
1 Title weidengguo-20191128-yz-k17
2 Origin Bruker BioSpin GmbH
3 Owner nmrsu
4 Solvent CDCl3
5 Temperature 298.0
6 Pulse Sequence zg30
7 Number of Scans 16
8 Relaxation Delay 1.0000
9 Pulse Width 9.9100
10 Acquisition Time 2.7263
11 Acquisition Date 2019-11-28T18:53:00
12 Modification Date 2019-11-28T18:53:34
2.02 2.03 1.03 1.00 1.03 0.99 0.98 2.04 3.05 3.05
f1 (ppm)

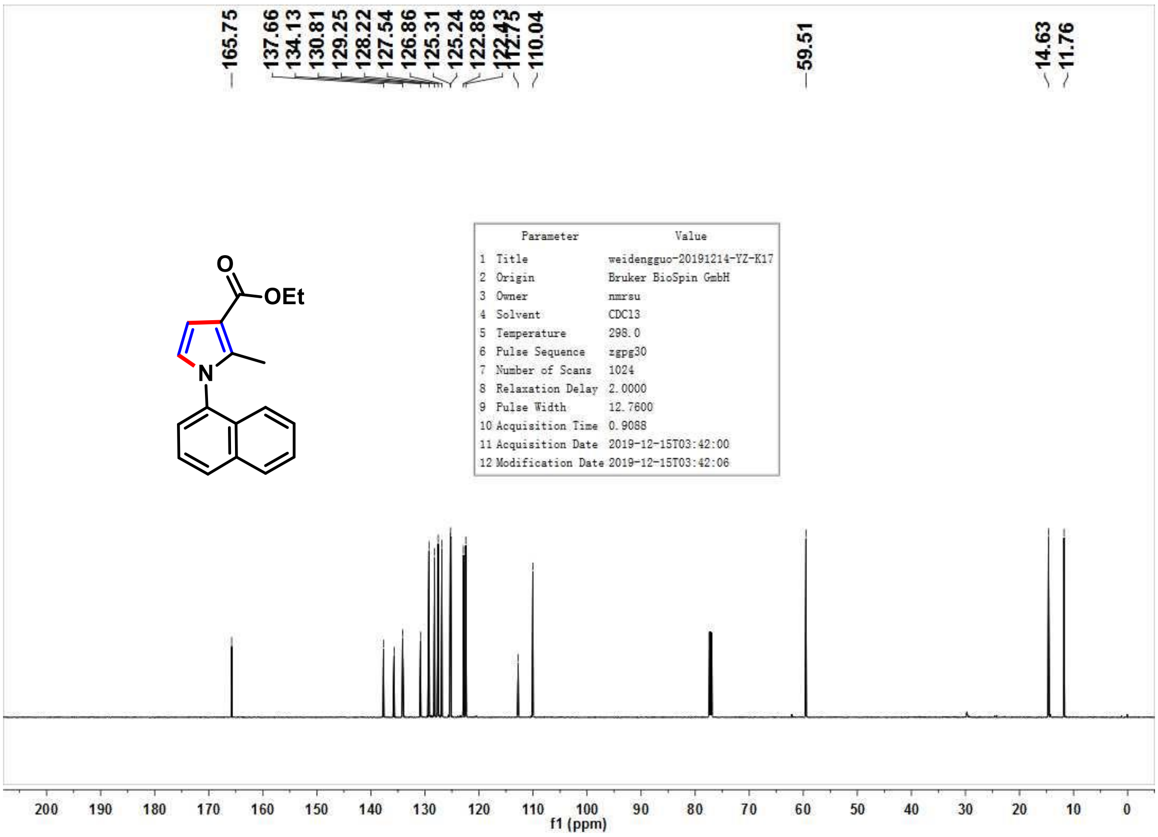
165.75
137.66 134.13 130.81 129.25 128.22 127.54 126.86 125.31 125.24 122.88 122.43 112.75 110.04
59.51
14.63 11.76
O
OEt
N
Parameter Value
1 Title weidengguo-20191214-YZ-K17
2 Origin Bruker BioSpin GmbH
3 Owner nmrsu
4 Solvent CDCl3
5 Temperature 298.0
6 Pulse Sequence zgpg30
7 Number of Scans 1024
8 Relaxation Delay 2.0000
9 Pulse Width 12.7600
10 Acquisition Time 0.9088
11 Acquisition Date 2019-12-15T03:42:00
12 Modification Date 2019-12-15T03:42:06
f1 (ppm)

4t

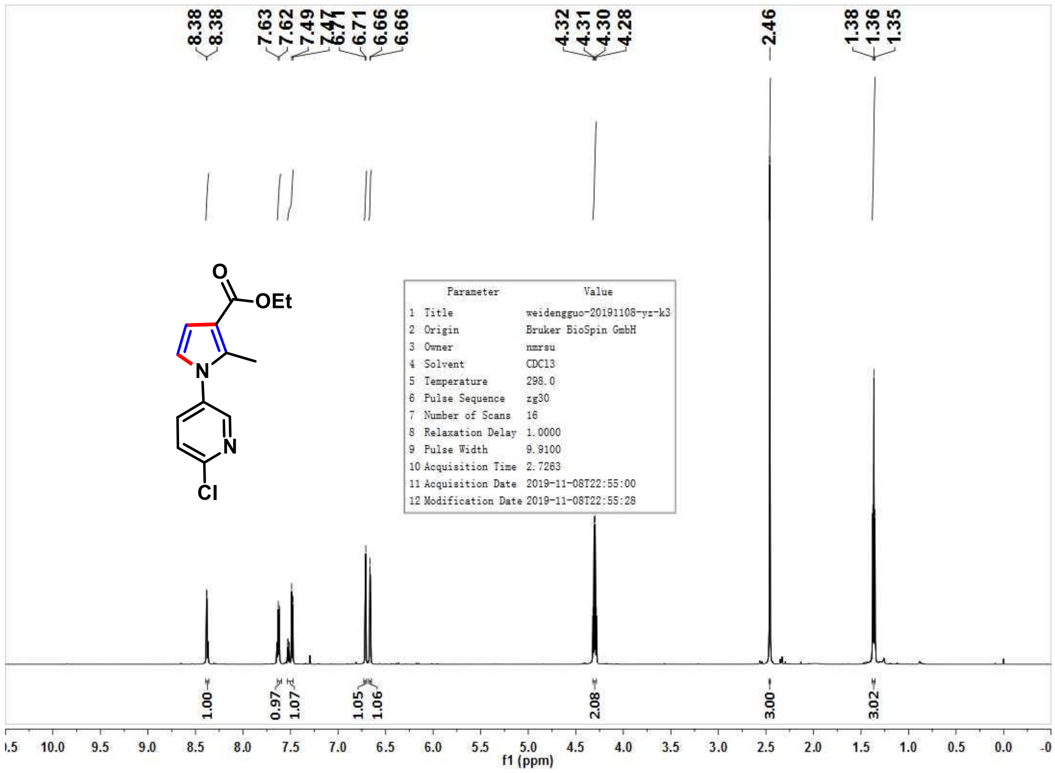

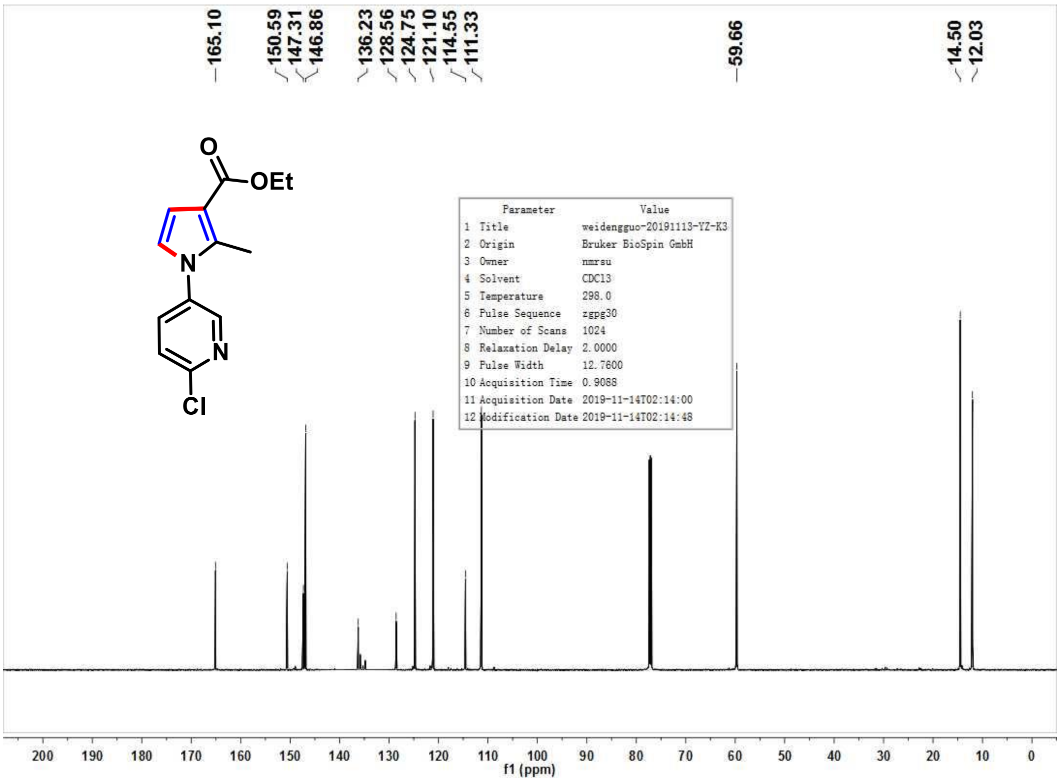

4u

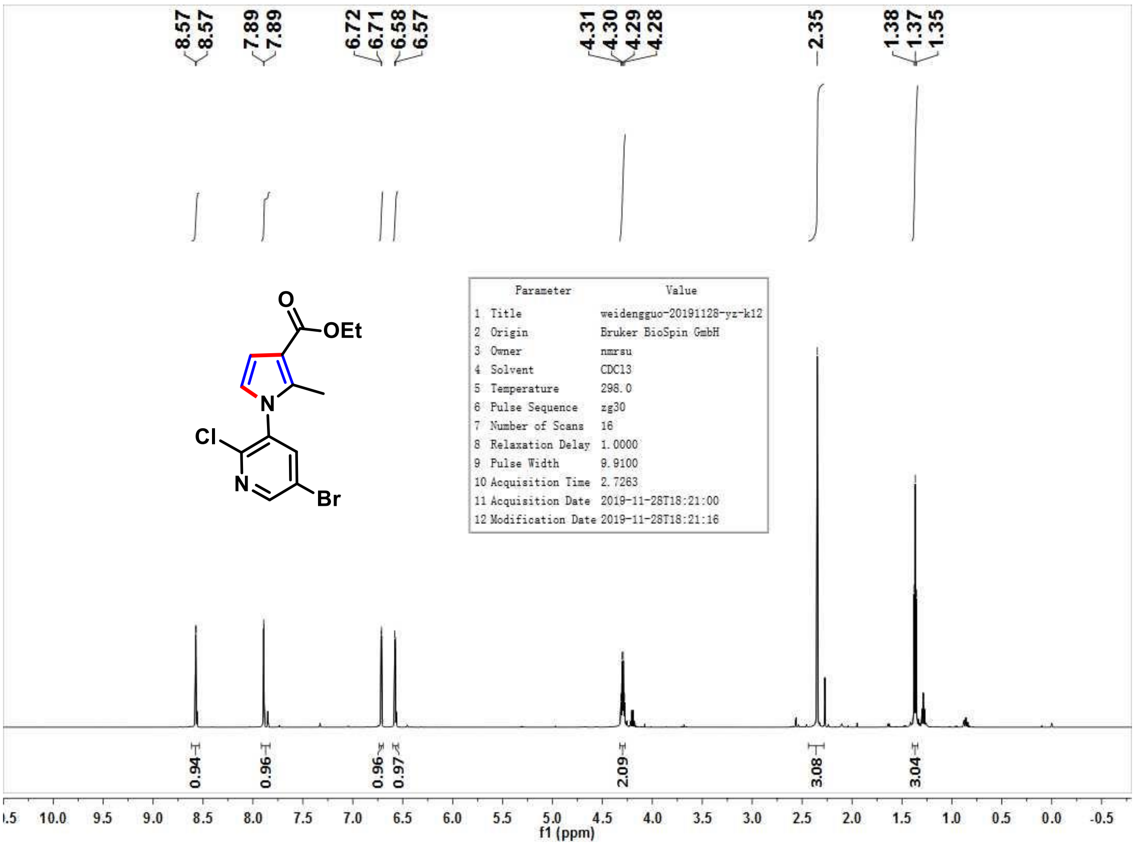
8.57
8.57
7.89
7.89
6.72
6.71
6.58
6.57
4.31
4.30
4.29
4.28
2.35
1.38
1.37
1.35
O
OEt
N
Cl
N
Br
Parameter Value
1 Title weidengguo-20191128-yz-k12
2 Origin Bruker BioSpin GmbH
3 Owner nmrsu
4 Solvent CDCl3
5 Temperature 298.0
6 Pulse Sequence zg30
7 Number of Scans 16
8 Relaxation Delay 1.0000
9 Pulse Width 9.9100
10 Acquisition Time 2.7263
11 Acquisition Date 2019-11-28T18:21:00
12 Modification Date 2019-11-28T18:21:16
0.94
0.96
0.96
0.97
2.09
3.08
3.04
f1 (ppm)

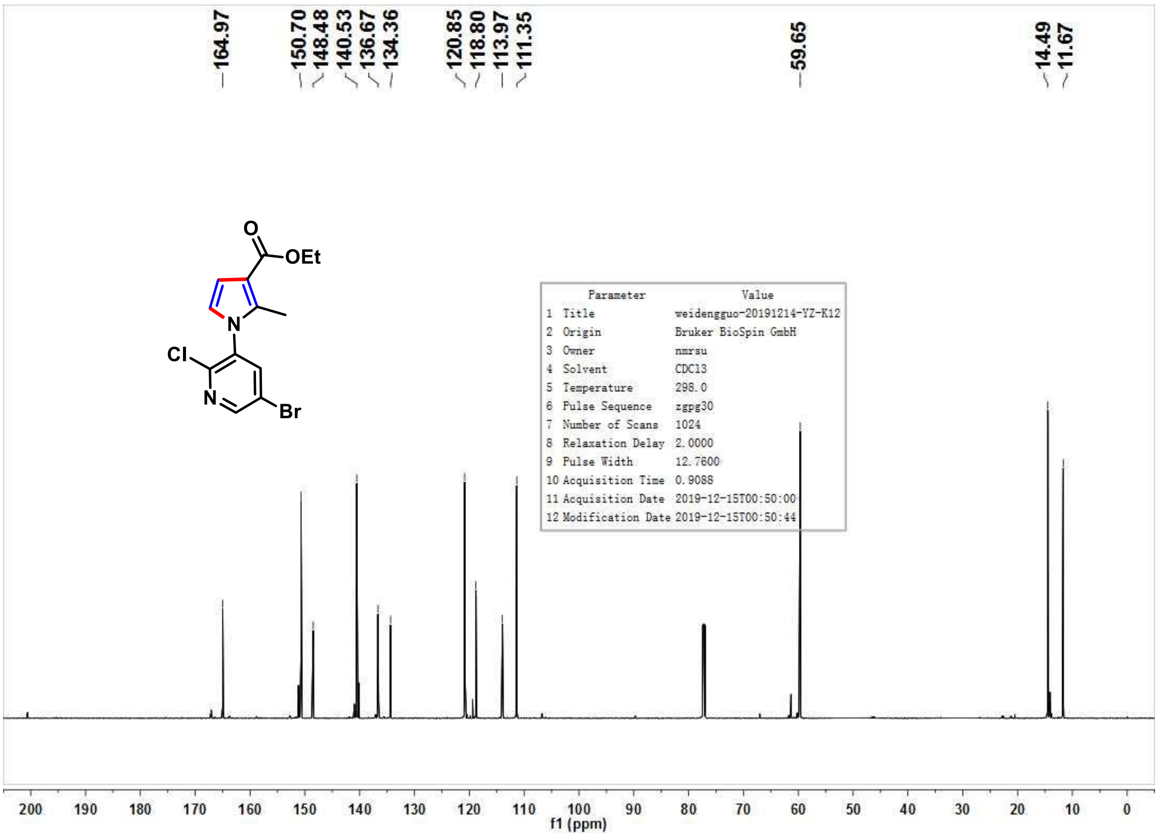
164.97
150.70
148.48
140.53
136.67
134.36
120.85
118.80
113.97
111.35
59.65
14.49
11.67
O
OEt
N
Cl
N
Br
Parameter Value
1 Title weidengguo-20191214-YZ-K12
2 Origin Bruker BioSpin GmbH
3 Owner nmrsu
4 Solvent CDCl3
5 Temperature 298.0
6 Pulse Sequence zgpg30
7 Number of Scans 1024
8 Relaxation Delay 2.0000
9 Pulse Width 12.7600
10 Acquisition Time 0.9088
11 Acquisition Date 2019-12-15T00:50:00
12 Modification Date 2019-12-15T00:50:44
f1 (ppm)

4v

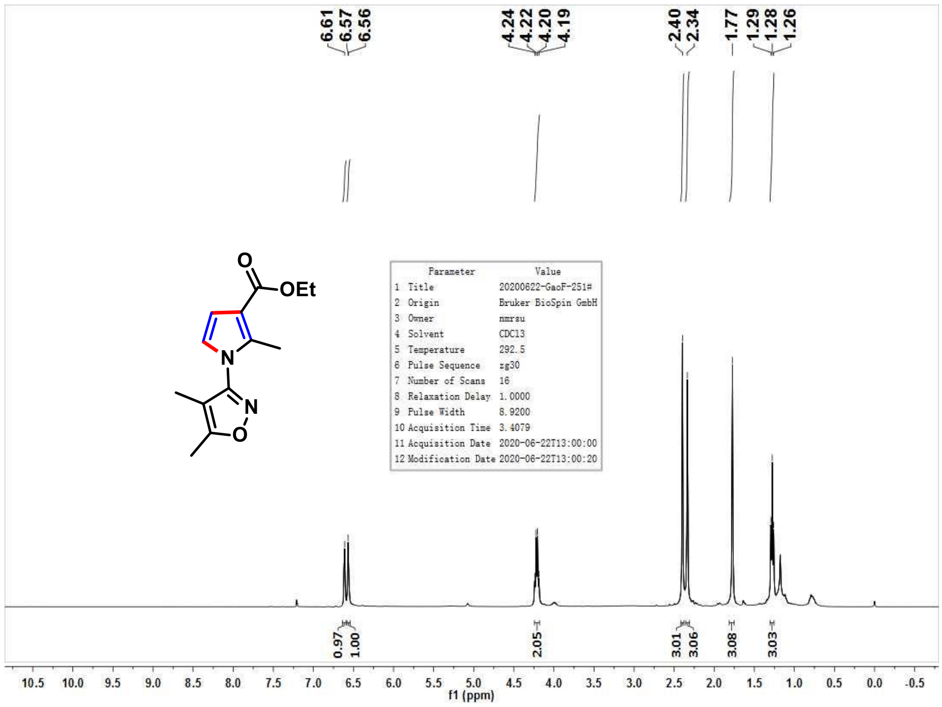
6.61
6.57
6.56
4.24
4.22
4.20
4.19
2.40
2.34
1.77
1.29
1.28
1.26
O
OEt
N
N
O
Parameter Value
1 Title 20200622-GaoF-251#
2 Origin Bruker BioSpin GmbH
3 Owner nmrsu
4 Solvent CDCl3
5 Temperature 292.5
6 Pulse Sequence zg30
7 Number of Scans 16
8 Relaxation Delay 1.0000
9 Pulse Width 8.9200
10 Acquisition Time 3.4079
11 Acquisition Date 2020-06-22T13:00:00
12 Modification Date 2020-06-22T13:00:20
0.97
1.00
2.05
3.01
3.06
3.08
3.03
f1 (ppm)

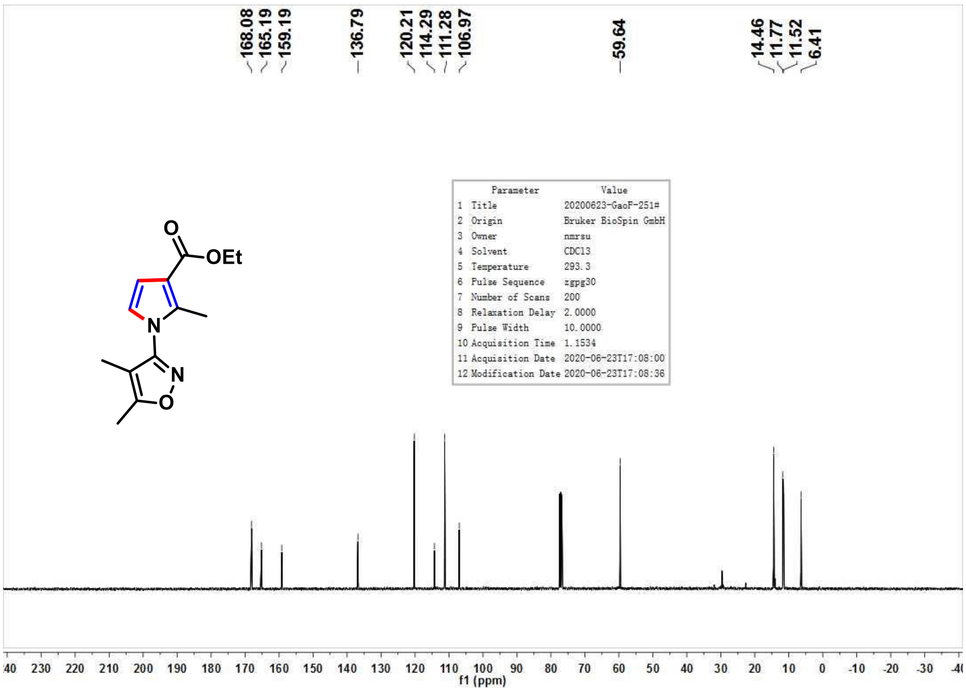
168.08
165.19
159.19
136.79
120.21
114.29
111.28
106.97
59.64
14.46
11.77
11.52
6.41
O
OEt
N
N
O
Parameter Value
1 Title 20200623-GaoF-251#
2 Origin Bruker BioSpin GmbH
3 Owner nmrsu
4 Solvent CDCl3
5 Temperature 293.3
6 Pulse Sequence zgpg30
7 Number of Scans 200
8 Relaxation Delay 2.0000
9 Pulse Width 10.0000
10 Acquisition Time 1.1534
11 Acquisition Date 2020-06-23T17:08:00
12 Modification Date 2020-06-23T17:08:36
f1 (ppm)

4x

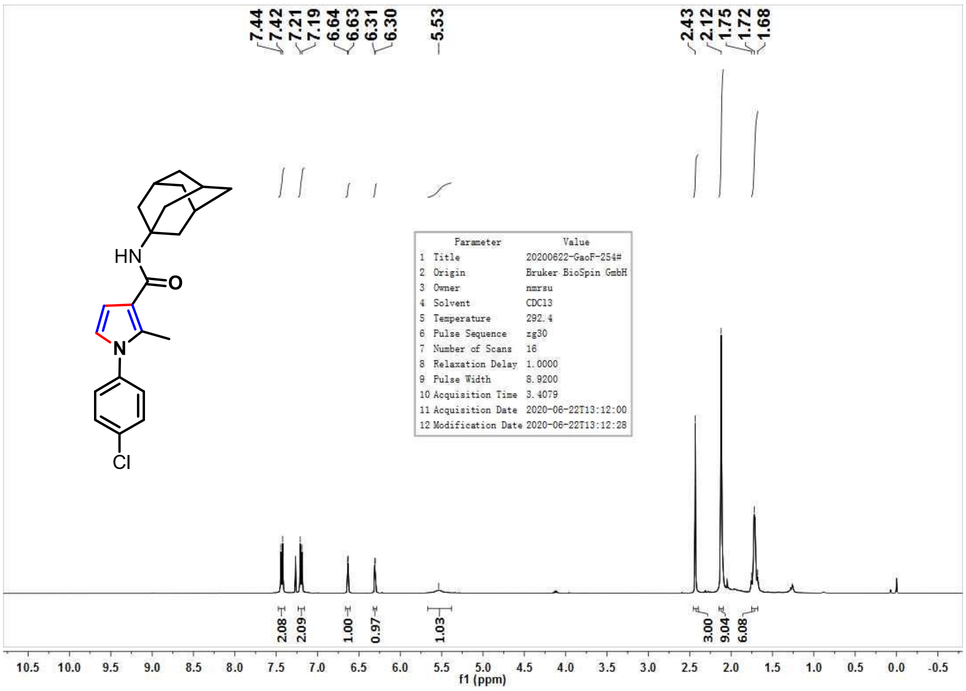
7.44
7.42
7.21
7.19
6.64
6.63
6.31
6.30
5.53
2.43
2.12
1.75
1.72
1.68
HN
O
N
Cl
Parameter Value
1 Title 20200622-GaoF-254#
2 Origin Bruker BioSpin GmbH
3 Owner nmrsu
4 Solvent CDCl3
5 Temperature 292.4
6 Pulse Sequence zg30
7 Number of Scans 16
8 Relaxation Delay 1.0000
9 Pulse Width 8.9200
10 Acquisition Time 3.4079
11 Acquisition Date 2020-06-22T13:12:00
12 Modification Date 2020-06-22T13:12:28
2.08
2.09
1.00
0.97
1.03
3.00
9.04
6.08
f1 (ppm)

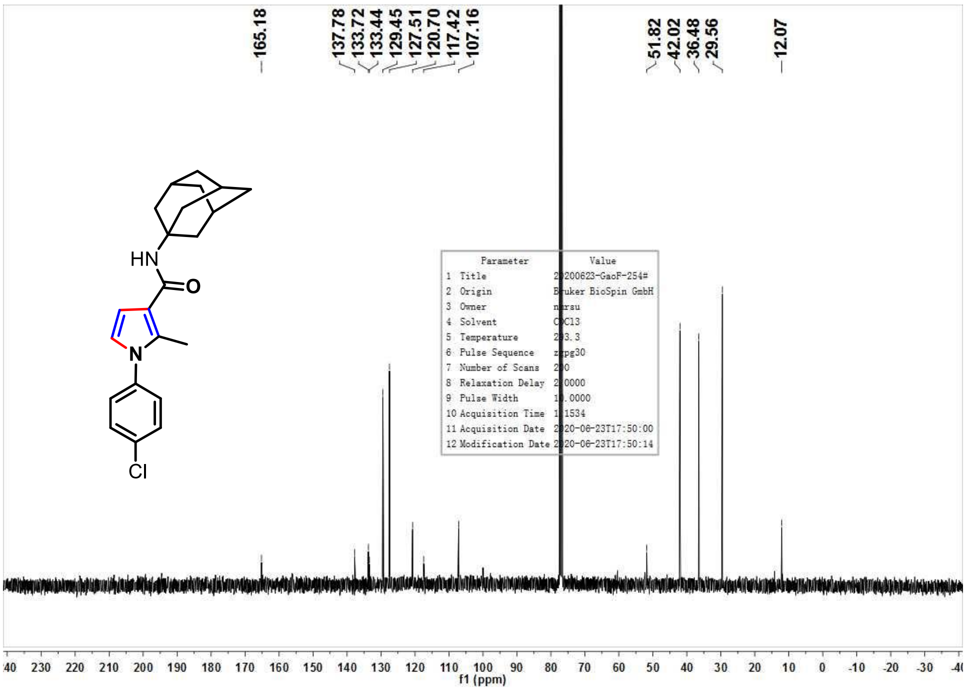
165.18
137.78
133.72
133.44
129.45
127.51
120.70
117.42
107.16
51.82
42.02
36.48
29.56
12.07
HN
O
N
Cl
Parameter Value
1 Title 20200623-GaoF-254#
2 Origin Bruker BioSpin GmbH
3 Owner nmrsu
4 Solvent CDCl3
f1 (ppm)

4y

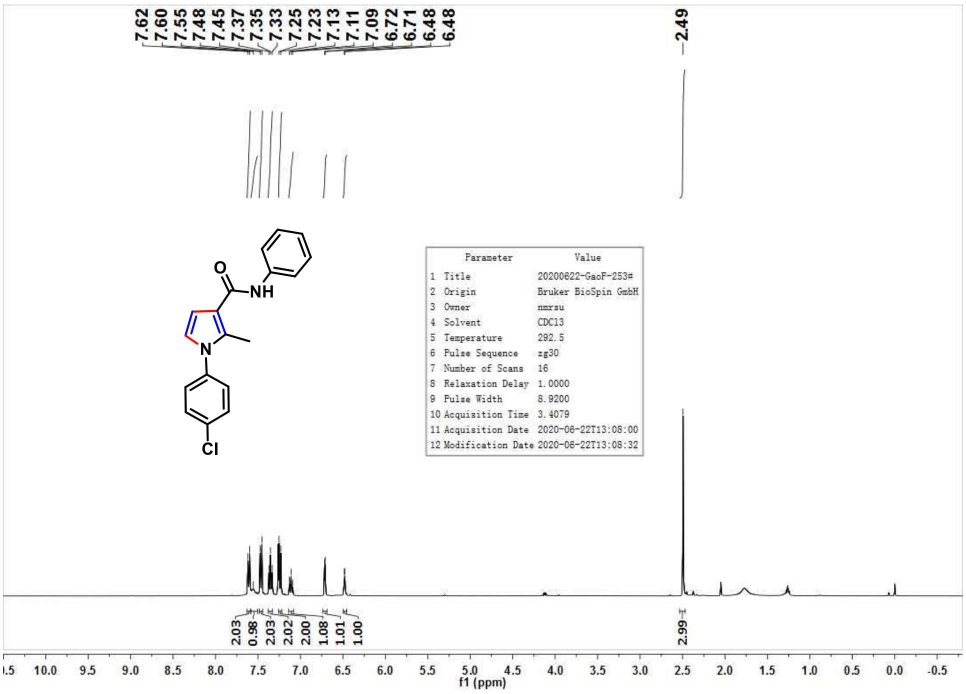
7.62
7.60
7.55
7.48
7.45
7.37
7.35
7.33
7.25
7.23
7.13
7.11
7.09
6.72
6.71
6.48
6.48
2.49
O
NH
N
Cl
Parameter Value
1 Title 20200622-GaoF-253#
2 Origin Bruker BioSpin GmbH
3 Owner nmrsu
4 Solvent CDCl3
5 Temperature 292.5
6 Pulse Sequence zg30
7 Number of Scans 16
8 Relaxation Delay 1.0000
9 Pulse Width 8.9200
10 Acquisition Time 3.4079
11 Acquisition Date 2020-06-22T13:08:00
12 Modification Date 2020-06-22T13:08:32
2.03
0.98
2.03
2.02
2.00
1.08
1.01
1.00
2.99
10.0 9.5 9.0 8.5 8.0 7.5 7.0 6.5 6.0 5.5 5.0 4.5 4.0 3.5 3.0 2.5 2.0 1.5 1.0 0.5 0.0 -0.5
f1 (ppm)

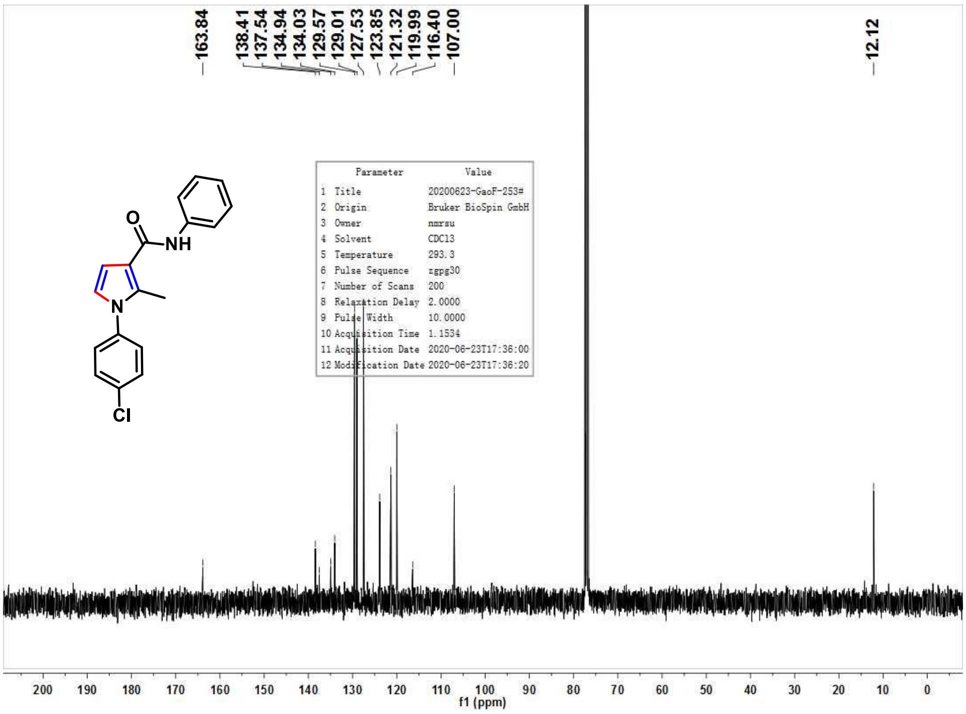
163.84
138.41
137.54
134.94
134.03
129.57
129.01
127.53
123.85
121.32
119.99
116.40
107.00
12.12
O
NH
N
Cl
Parameter Value
1 Title 20200623-GaoF-253#
2 Origin Bruker BioSpin GmbH
3 Owner nmrsu
4 Solvent CDCl3
5 Temperature 293.3
6 Pulse Sequence zgpg30
7 Number of Scans 200
8 Relaxation Delay 2.0000
9 Pulse Width 10.0000
10 Acquisition Time 1.1534
11 Acquisition Date 2020-06-23T17:36:00
12 Modification Date 2020-06-23T17:36:20
200 190 180 170 160 150 140 130 120 110 100 90 80 70 60 50 40 30 20 10 0
f1 (ppm)

5a

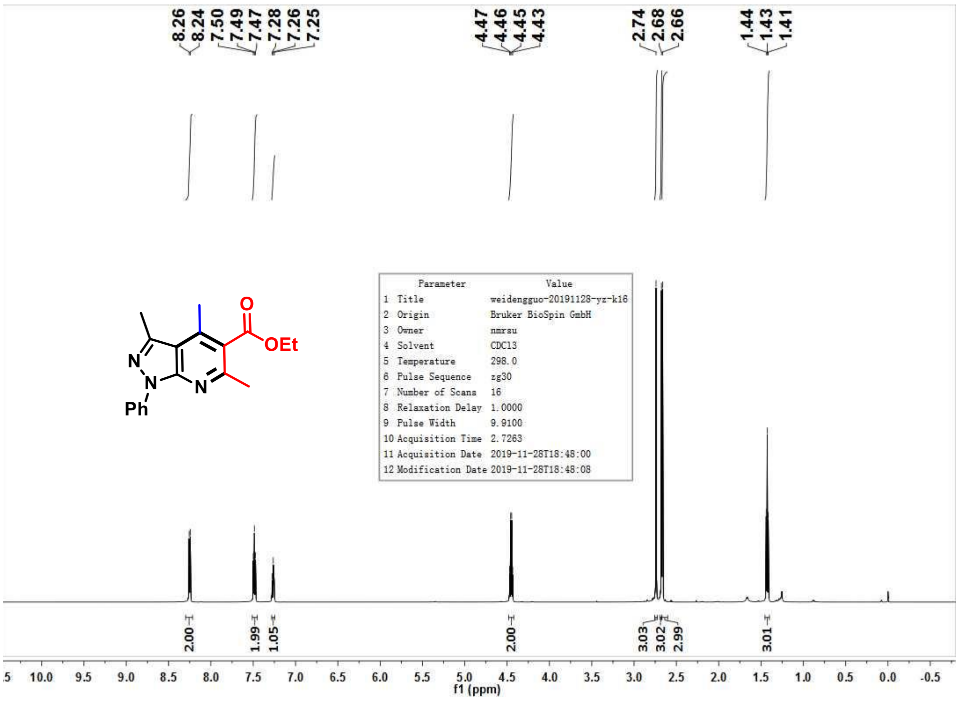
8.26
8.24
7.50
7.49
7.47
7.28
7.26
7.25
4.47
4.46
4.45
4.43
2.74
2.68
2.66
1.44
1.43
1.41
OEt
N
N
N
Ph
O
Parameter Value
1 Title weidengguo-20191128-yz-k16
2 Origin Bruker BioSpin GmbH
3 Owner nmrsu
4 Solvent CDCl3
5 Temperature 298.0
6 Pulse Sequence zg30
7 Number of Scans 16
8 Relaxation Delay 1.0000
9 Pulse Width 9.9100
10 Acquisition Time 2.7263
11 Acquisition Date 2019-11-28T18:48:00
12 Modification Date 2019-11-28T18:48:08
2.00
1.99
1.05
2.00
3.03
3.02
2.99
3.01
f1 (ppm)

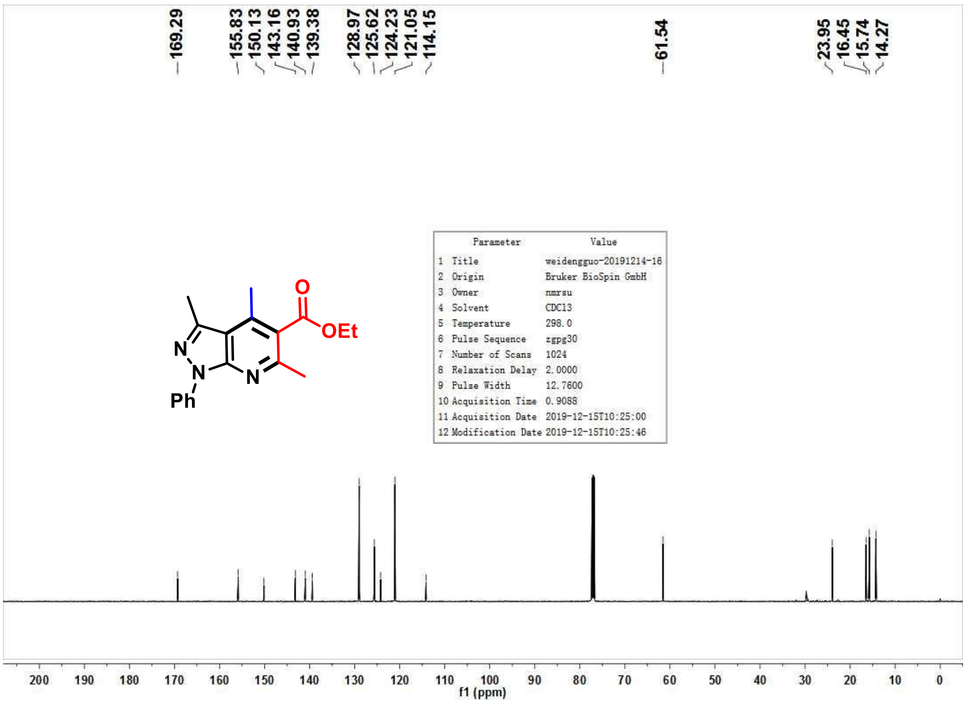
169.29
155.83
150.13
143.16
140.93
139.38
128.97
125.62
124.23
121.05
114.15
61.54
23.95
16.45
15.74
14.27
OEt
N
N
N
Ph
O
Parameter Value
1 Title weidengguo-20191214-16
2 Origin Bruker BioSpin GmbH
3 Owner nmrsu
4 Solvent CDCl3
5 Temperature 298.0
6 Pulse Sequence zgpg30
7 Number of Scans 1024
8 Relaxation Delay 2.0000
9 Pulse Width 12.7600
10 Acquisition Time 0.9088
11 Acquisition Date 2019-12-15T10:25:00
12 Modification Date 2019-12-15T10:25:46
f1 (ppm)
